# Strain‐Release Pentafluorosulfanylation and Tetrafluoro(aryl)sulfanylation of [1.1.1]Propellane: Reactivity and Structural Insight**

**DOI:** 10.1002/anie.202211892

**Published:** 2022-10-25

**Authors:** Yannick Kraemer, Clément Ghiazza, Abbey N. Ragan, Shengyang Ni, Sigrid Lutz, Elizabeth K. Neumann, James C. Fettinger, Nils Nöthling, Richard Goddard, Josep Cornella, Cody Ross Pitts

**Affiliations:** ^1^ Department of Chemistry University of California, Davis 1 Shields Avenue Davis CA 95616 USA; ^2^ Max-Planck-Institut für Kohlenforschung Kaiser-Wilhelm-Platz 1 45470 Mülheim an der Ruhr Germany

**Keywords:** Main Group Chemistry, Pentafluorosulfanyl, Propellane, Strained Molecules, X-Ray Diffraction

## Abstract

We leveraged the recent increase in synthetic accessibility of SF_5_Cl and Ar−SF_4_Cl compounds to combine chemistry of the SF_5_ and SF_4_Ar groups with strain‐release functionalization. By effectively adding SF_5_ and SF_4_Ar radicals across [1.1.1]propellane, we accessed structurally unique bicyclopentanes, bearing two distinct elements of bioisosterism. Upon evaluating these “hybrid isostere” motifs in the solid state, we measured exceptionally short transannular distances; in one case, *the distance rivals the shortest nonbonding C*⋅⋅⋅*C contact reported to date*. This prompted SC‐XRD and DFT analyses that support the notion that a donor‐acceptor interaction involving the “wing” C−C bonds is playing an important role in stabilization. Thus, these heretofore unknown structures expand the palette for highly coveted three‐dimensional fluorinated building blocks and provide insight to a more general effect observed in bicyclopentanes.

## Graphical abstract

Merging SF_5_ and Ar−SF_4_ chemistry with strain‐release functionalization of [1.1.1]propellane enables us to explore uncharted chemical space associated with C(sp^3^)−SF_5_ and C(sp^3^)−SF_4_Ar motifs. X‐ray analyses of the resultant “hybrid isosteres” reveal remarkably short transannular nonbonding C⋅⋅⋅C contacts—among the shortest distances reported to date. DFT and SC‐XRD data provide evidence that the wing C−C bonds play a key role in stabilization.

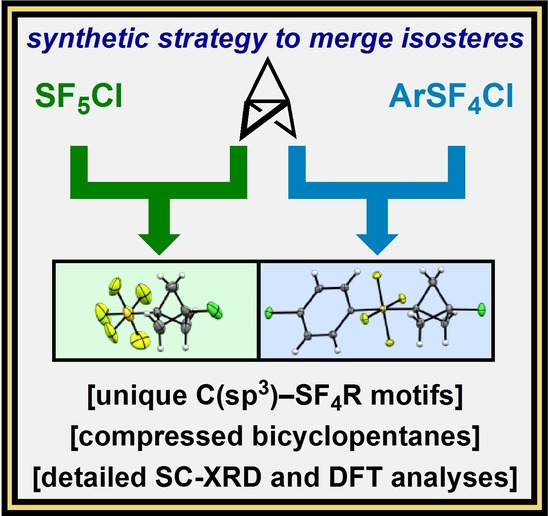

## Introduction

The pentafluorosulfanyl (SF_5_) group is situated on an exclusive list of fluorinated functional groups with documented utility that remain underemployed.^[1]^ The reason for this dissonance is attributed to limited pentafluorosulfanylation methods and reagents, particularly with respect to introducing the SF_5_ group to aliphatic frameworks.^[2]^ For instance, SF_5_Cl gas—one of the few reliable sources of SF_5_ radicals with applications in C(sp^3^)−SF_5_ synthesis^[3]^—has been historically difficult to synthesize and handle,^[4]^ thus preventing widespread adoption of the chemistry and impeding further innovation. This contrasts with the relative accessibility of its Ar−SF_4_Cl congeners,^[5]^ which are analogous sources of Ar−SF_4_ radicals with applications in C(sp^3^)−SF_4_Ar synthesis.^[6]^

However, as part of a larger effort to make polyfluorinated groups more accessible in 2019,^[7]^ one of us disclosed the first user‐friendly, gas‐reagent free synthesis of SF_5_Cl in the patent literature through oxidative fluorination of S_8_ using inexpensive trichloroisocyanuric acid (TCICA) and potassium fluoride.^[8]^ In 2021, Qing and co‐workers reported an optimized workup protocol for this TCICA/KF approach whereby SF_5_Cl can be extracted directly into hexanes to produce a storable stock solution, *thus obviating the need to handle SF_5_Cl as a gas*.^[9]^ Together, these recent advancements not only enable the broader chemical community to synthesize their own SF_5_Cl solution more safely, but facilitates the invention of pentafluorosulfanylation reactions and the study of heretofore unknown SF_5_‐containing motifs.^[10]^

**Figure 1:**
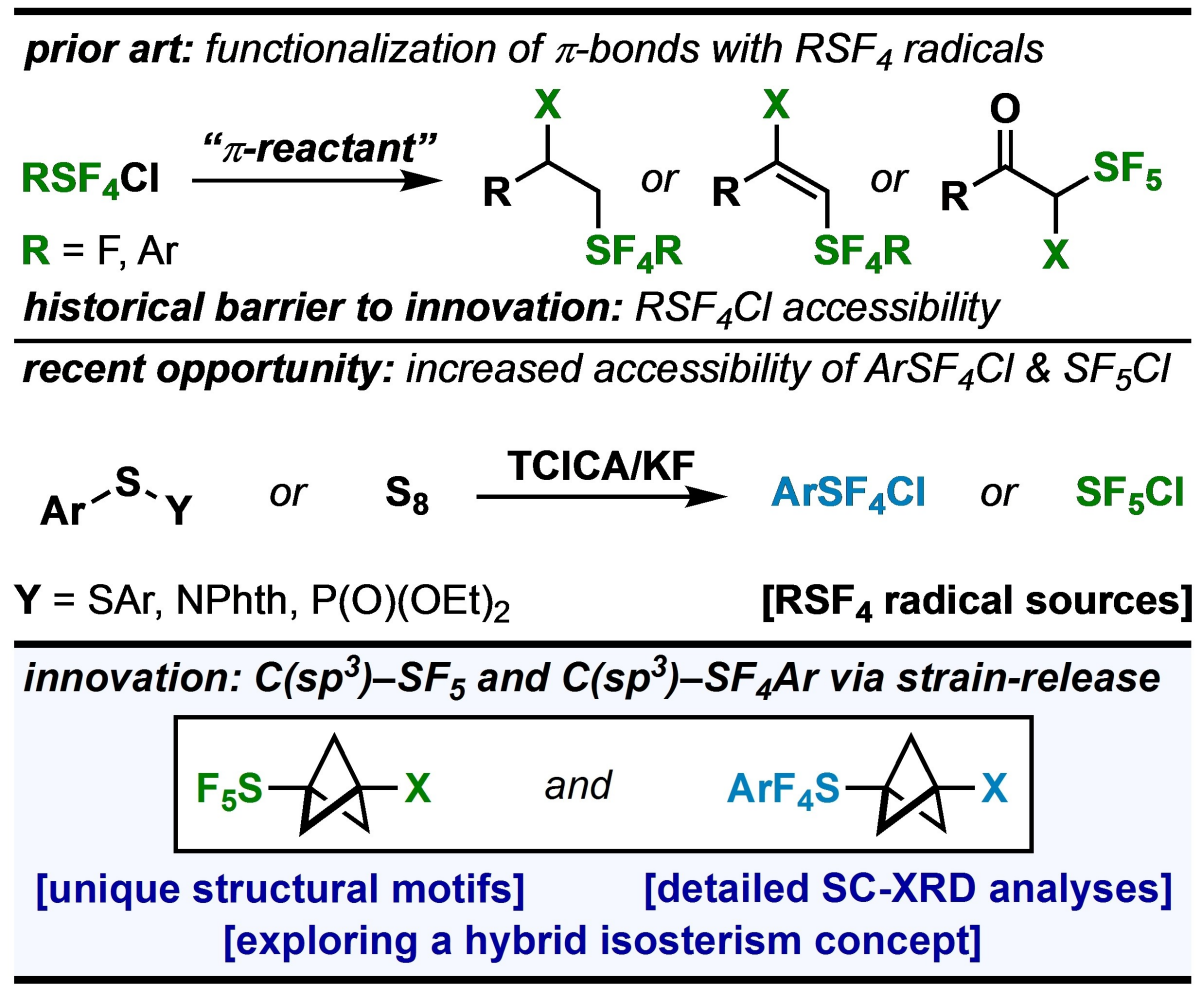
Strain‐release pentafluoro‐ and tetrafluoro(aryl)sulfanylation in context. (Top panel) Known reactivity of SF_4_R radicals and barrier to innovation. (Middle panel) Recent increased accessibility of key reagents. (Bottom panel) This work.

To date, the known methods of forming C(sp^3^)−SF_5_ and C(sp^3^)−SF_4_Ar bonds from either SF_5_ or Ar−SF_4_ radicals involve formal addition to π‐systems (Figure 1, *top*). Alternatively, with the key reagents more accessible (Figure 1, *middle*), we sought opportunities to add SF_5_Cl and Ar−SF_4_Cl compounds across other types of bonds such as the central bond in [1.1.1]propellane.^[11]^ Conceptually, the result of combining SF_5_ radical chemistry with strain‐release functionalization^[12]^ would enable investigation of the structural consequences of merging a bioisosteric replacement for phenyl groups or alkynes (i.e., bicyclopentane, or “BCP”)^[13]^ with a bioisosteric surrogate for trifluoromethyl or *tert*‐butyl groups (i.e., SF_5_).^[14]^ Additionally, appending an Ar−SF_4_ moiety with a BCP ring would provide access to another type of hybrid isostere that may be of interest in the design of materials, such as liquid crystals.^[15]^

Herein, we report a mild, radical pentafluorosulfanylation and tetrafluoro(aryl)sulfanylation of [1.1.1]propellane (Figure 1, *bottom*). Based on a detailed structural analysis of this unusual SF_5_−BCP motif, we also found that the resulting transannular C⋅⋅⋅C distance in SF_5_−BCP−Cl is one of the shortest allegedly nonbonding C⋅⋅⋅C distances reported in the CSD (see Supporting Information for search details). This prompted synthesis of a suite of tetrafluoro(aryl)sulfanyl derivatives and extensive single‐crystal X‐ray diffraction (SC‐XRD) and NMR comparative analyses. Furthermore, the nature of the dramatic impact of the SF_5_ group on the BCP ring scaffold was studied further using density functional theory (DFT).

## Results and Discussion

**Figure 2:**
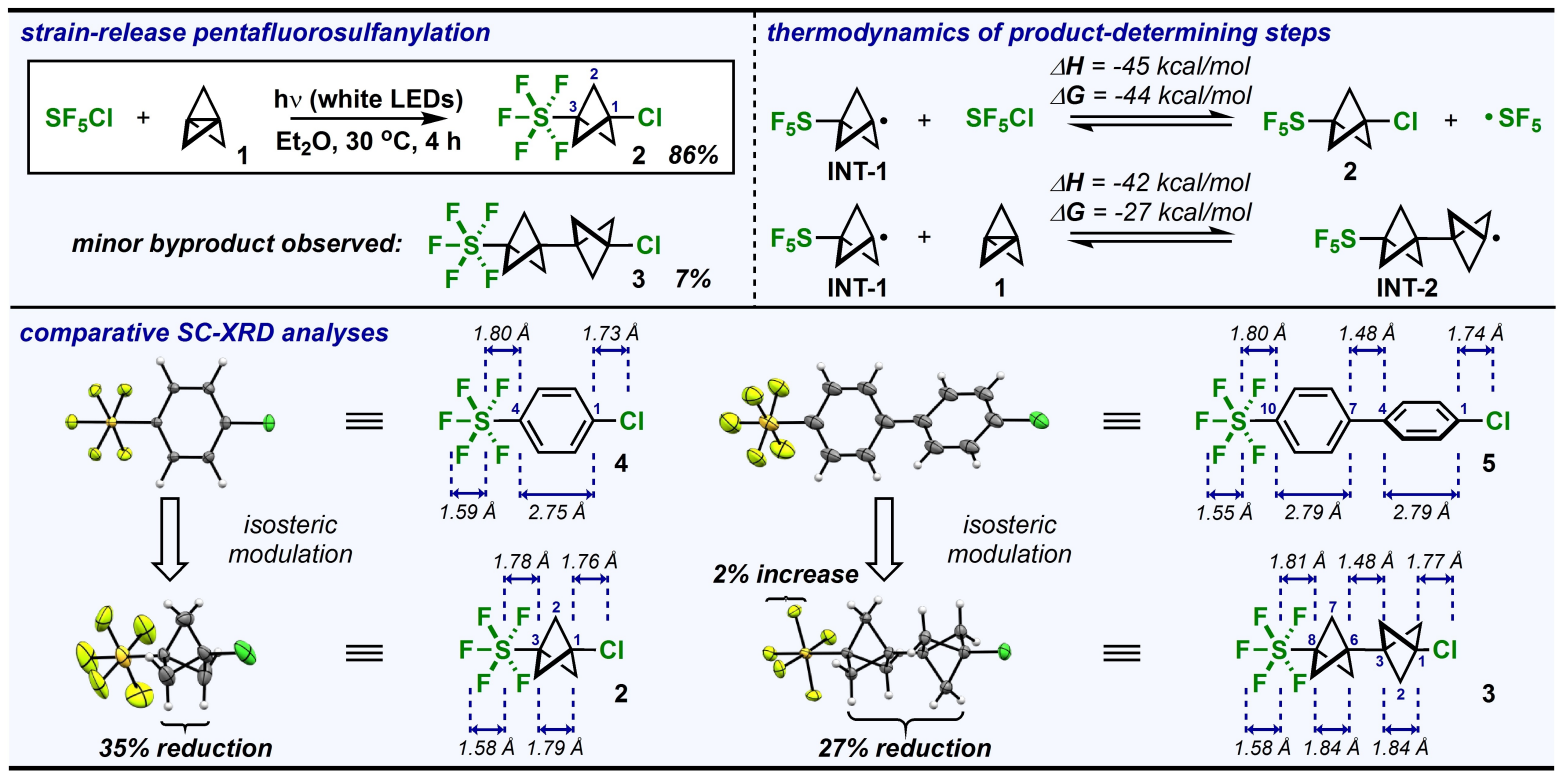
(Top left panel) Major product **2** (and minor byproduct **3**) observed under optimized pentafluorosulfanylation conditions, using 1.5 equiv SF_5_Cl and 1.0 equiv **1**. Isolated yields reported. (Top right panel) Thermodynamic parameters associated with the product‐determining steps, calculated at ωB97XD/6‐311++G** using the default Et_2_O solvent continuum. (Bottom panel) Structural differences in the linear array of atoms upon net replacement of benzene rings in **4** and **5** with bicyclopentane (BCP) rings to make “hybrid” SF_5_−BCP isosteres **2** and **3**. Structures determined by single‐crystal X‐ray diffraction (displacement ellipsoids depicted at 50 % probability). Only non‐disordered parts are shown for clarity.

We began our investigation by applying a well‐established strategy to generate SF_5_ radicals from SF_5_Cl (i.e., using catalytic BEt_3_/O_2_) in the presence of [1.1.1]propellane (**1**).^[16]^ The desired SF_5_−BCP−Cl product (**2**) was observed by ^19^F NMR in a promising 48 % yield.^[17]^ This prompted an optimization campaign that led to the discovery that irradiating SF_5_Cl and **1** with white LEDs for 4 h would ultimately allow isolation of volatile **2** in 86 % yield (Figure 2, *top left*).

Based on previous literature on both SF_5_Cl and **1**, a radical chain propagation mechanism is most likely at play following initial formation of SF_5_ radicals (see Supporting Information for calculated free energy profiles).^[18]^ Control experiments indicate that SF_5_Cl does not undergo decomposition under irradiation with white LEDs in the absence of **1**, suggesting **1** is involved in the initiation step.^[19]^ It is plausible that SF_5_ radicals are formed initially through visible light‐excitation of an electron donor‐acceptor complex between SF_5_Cl and **1** and then propagate a radical chain reaction, similar to the mechanism proposed by Paquin and co‐workers.^[3b]^ Additionally, we isolated a minor byproduct (**3**) in 7 % yield that incorporates two bicyclopentane motifs; similar types of dimerization products (i.e., “[2]staffanes”) have been observed previously in radical chain processes involving **1**.^[20]^ The difference in free energies calculated at ωB97XD/6‐311++G** (using the default Et_2_O solvent continuum) for the product determining steps are also consistent with our observed selectivity, as the path from putative **INT‐1** to **2** is 17 kcal mol^−1^ more exergonic than to **INT‐2** (Figure 2, *top right*).

After much effort, we also managed to grow single crystals of **2** suitable for X‐ray diffraction to examine the SF_5_−BCP motif in the solid state at 100 K (Figure 2, *bottom*). The most striking feature is the exceptionally short transannular C_1_⋅⋅⋅C_3_ distance of 1.789(6) Å (libration corrected value: 1.800 Å).^[21]^ It is notably within error of the transannular C_1_⋅⋅⋅C_3_ distance reported for Adcock and co‐workers’ pyridinium‐substituted bicyclopentane—1.80(2) Å—which they claimed at the time was, “*not only the shortest nonbonding contact for bicyclo[1.1.1]pentanes but also for any known organic compound*.”^[22]^ To put it in perspective, the transannular distance in **2** is significantly shorter than in an unsubstituted [1.1.1]bicyclopentane (with a transannular distance of ≈1.85–1.87 Å)^[23]^ and is approaching that of one of the longest C−C bonds reported (i.e., 1.780(7) Å).^[24]^

To put the structural features of the linear array of atoms about the C_1_‐C_3_ axis of **2** into context, we obtained SC‐XRD data for SF_5_−Ph−Cl (**4**). This allowed us to assess the consequences of Ph→BCP isosteric replacement in the presence of an SF_5_ group (Figure 2, *bottom*). While maintaining a relatively linear central axis, the C_1_⋅⋅⋅C_3_ transannular distance in **2** is attenuated by ≈35 % relative to the C_1_⋅⋅⋅C_4_ distance in **4** (2.745(1) Å). The C−S bond in **2** is marginally shorter than in **4** (1.779(4) Å^[21]^ vs. 1.7952(6) Å, respectively), while the C−Cl bond in **2** is slightly longer than in **4** (1.759(5) Å^[21]^ vs. 1.7302(6) Å, respectively). The equatorial fluorine (F_eq_) atoms of the SF_5_ group on both **2** and **4** sport average S−F_eq_ distances of ≈1.57–1.59 Å, and *θ*_*C3‐S‐Feq*_ for both **2** and **4** deviates only slightly from perpendicularity on average (i.e., ≈92.3° vs. ≈92.6°). We also noted that the axial S−F_a*x*_ bond length in **2** at 1.579(4) Å^[21]^ is within range of **4** (1.5869(5) Å).

Additionally, we obtained SC‐XRD data for the [2]staffane byproduct—SF_5_−BCP−BCP−Cl (**3**)—to compare alongside **2** as well as the previously published structure of its SF_5_−Ph−Ph−Cl congener, **5** (Figure 2, *bottom*).^[25]^ In this case, both transannular C⋅⋅⋅C distances in the bicyclopentane rings of **3** (1.835(4) Å and 1.839(4) Å) are notably longer than that of **2**. Alternatively, the conduit C−C bonds connecting the ring structures in **3** and **5** are similar in length; this makes the composite effect of replacing both Ph rings in the biphenyl array with BCP rings a ≈27 % reduction in distance between the terminal carbon atoms. Also, similar to **2** vs. **4**, the C−Cl bond in **3** is longer than in **5**, and average S−F_eq_ distances are ≈1.58–1.60 Å in each case. However, the S−F_a*x*_ bond in **3** is ≈2 % longer than in **5**.

At this juncture, we envisioned that incorporation of an Ar−SF_4_ moiety on the BCP ring instead of SF_5_ would allow us to explore another concept in “hybrid isosterism,” as the *trans*‐substituted ‐SF_4_‐ linear architecture has been entertained as a replacement for an alkyne (or even a BCP ring).^[26]^ This would also prove advantageous for further crystallographic analyses, as modification of the aryl group allows us to examine effects of remote substitution on the BCP ring. Under identical conditions to the reaction of SF_5_Cl with **1**, we discovered that Ar−SF_4_Cl compounds can indeed be converted into the desired Ar−SF_4_−BCP−Cl products, albeit in modest yields. Thus, the reaction conditions were re‐optimized explicitly for Ar−SF_4_Cl addition across **1**.^[17]^ Note that, in this case, the reaction proceeds spontaneously in the dark at −45 °C. The propensity of Ar−SF_4_Cl compounds to react more readily with **1** than SF_5_Cl is consistent with the fact that the S−Cl bond has been determined to be weaker in Ar−SF_4_Cl compounds (i.e., computed BDEs are on the order of 6–7 kcal mol^−1^ lower than the S−Cl BDE in SF_5_Cl).^[6a]^ It is also known that both **1** ^[27]^ and Ar−SF_4_Cl compounds,^[28]^ independently, can initiate radical chain propagations spontaneously in some cases in the absence of a designated radical initiator or other initiation source, such as light.

**Figure 3:**
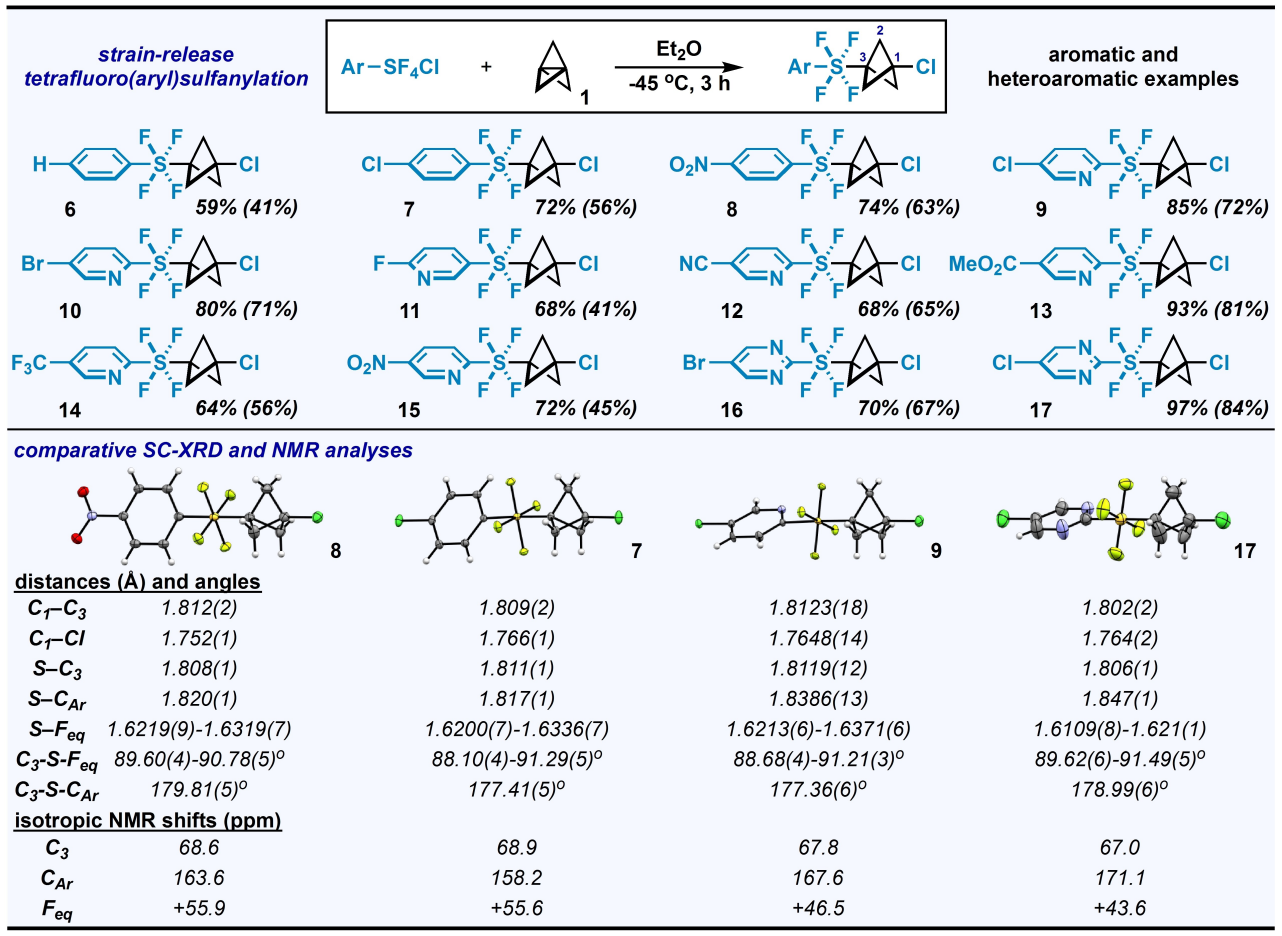
(Top panel) Reaction scope under optimized tetrafluoro(aryl)sulfanylation conditions, using 1.0 equiv ArSF_4_Cl and 1.2 equiv **1**. ^19^F NMR yields are reported; isolated yields are in parentheses. (Bottom panel) Structural differences and select ^13^C and ^19^F NMR data associated with the linear array of atoms upon systematic alteration of electronic properties of the (hetero)arene. Structures determined by single‐crystal X‐ray diffraction (displacement ellipsoids depicted at 50 % probability).

We then explored the scope of accessible arene‐ and heteroarene‐containing compounds under optimized conditions (Figure 3, *top*). For one, Ar−SF_4_Cl starting materials containing benzene rings—either unsubstituted or substituted with electron‐withdrawing groups—provided the desired addition products **6**–**8** in good yields. Moreover, starting materials containing pyridine rings with various halogen (**9**–**11**), cyano (**12**), ester (**13**), trifluoromethyl (**14**), and nitro (**15**) substituents performed generally well under specified conditions. Additionally, the scope includes examples of pyrimidine‐containing products, such as **16** and **17** ‐ the latter formed in 97 % yield by ^19^F NMR and isolated in 84 %. During these studies, we noted that pyridine‐ and pyrimidine‐based products of all substitution patterns were *generally* less prone to decomposition during attempted purification than benzene‐based products without a strong electron withdrawing substituent present (e.g., a nitro group), though we noted some exceptions (**6** and **7**). This phenomenon is in accord with what has been observed previously in compounds containing the Ar−SF_4_ moiety,^[6]^ and we have indicated a few examples of compounds in our Supporting Information where decomposition on silica gel (or basic alumina) was problematic, in contrast to the entries shown in Figure 3.

In the process of examining the reaction scope, we managed to obtain SC‐XRD data on compounds **7**, **8**, **9**, and **17** (Figure 3, *bottom*). In this suite, the C_1_⋅⋅⋅C_3_ transannular distances are still unusually short but all slightly longer than that of **2**,^[21]^ decreasing in the order of: **8** (1.812(2) Å) ≈**9** (1.8123(18) Å)>**7** (1.809(2) Å)>**17** (1.802(2) Å). The C_3_−S bonds (ranging from 1.806(1) Å in **17** to 1.8119(12) Å in **9**) and the S−F_eq_ bonds (average distances of ≈1.62–1.64 Å in the series) are also both notably longer in these Ar−SF_4_−BCP−Cl compounds than in **2**. The C_1_−Cl bond length in these four compounds varies but is centered around that of **2**, ranging from 1.752(1) Å in **8** to 1.766(1) Å in **7**. Another noticeable difference is that the *θ*_*C3‐S‐Feq*_ in these Ar−SF_4_−BCP−Cl compounds, on average, is closer to 90° than in **2**, where the equatorial fluorine atoms of the SF_5_ group seem to pucker away from the BCP ring. This could be attributed to an increased repulsive interaction between the equatorial fluorine atoms and the BCP ring in **2**, as **2** exhibits a shorter C_3_−S bond than the Ar−SF_4_−BCP−Cl compounds.

A trend can be found in the linear array of atoms in the four Ar−SF_4_−BCP−Cl compounds in Figure 3: the C_Ar_−S bond lengths increase as the (hetero)arenes become increasingly electron deficient, i.e., in the order of **7** (1.817(1) Å)<**8** (1.820(1) Å)<**9** (1.8386(13) Å)<**17** (1.847(1) Å). This type of trend has been observed previously in other compounds containing an Ar−SF_4_ motif and is associated with an increase in ionic character in the C_Ar_−S bond.[^6^, ^29^] The dramatic “downfield” shift in ^13^C NMR shift of the *ipso* carbon atom (C_Ar_) from **7** to **17** is also consistent with this idea (note that the cross‐ring C_3_ atom ^13^C shift is less impacted by remote arene functionalization but experiences the opposite trend).

**Figure 4:**
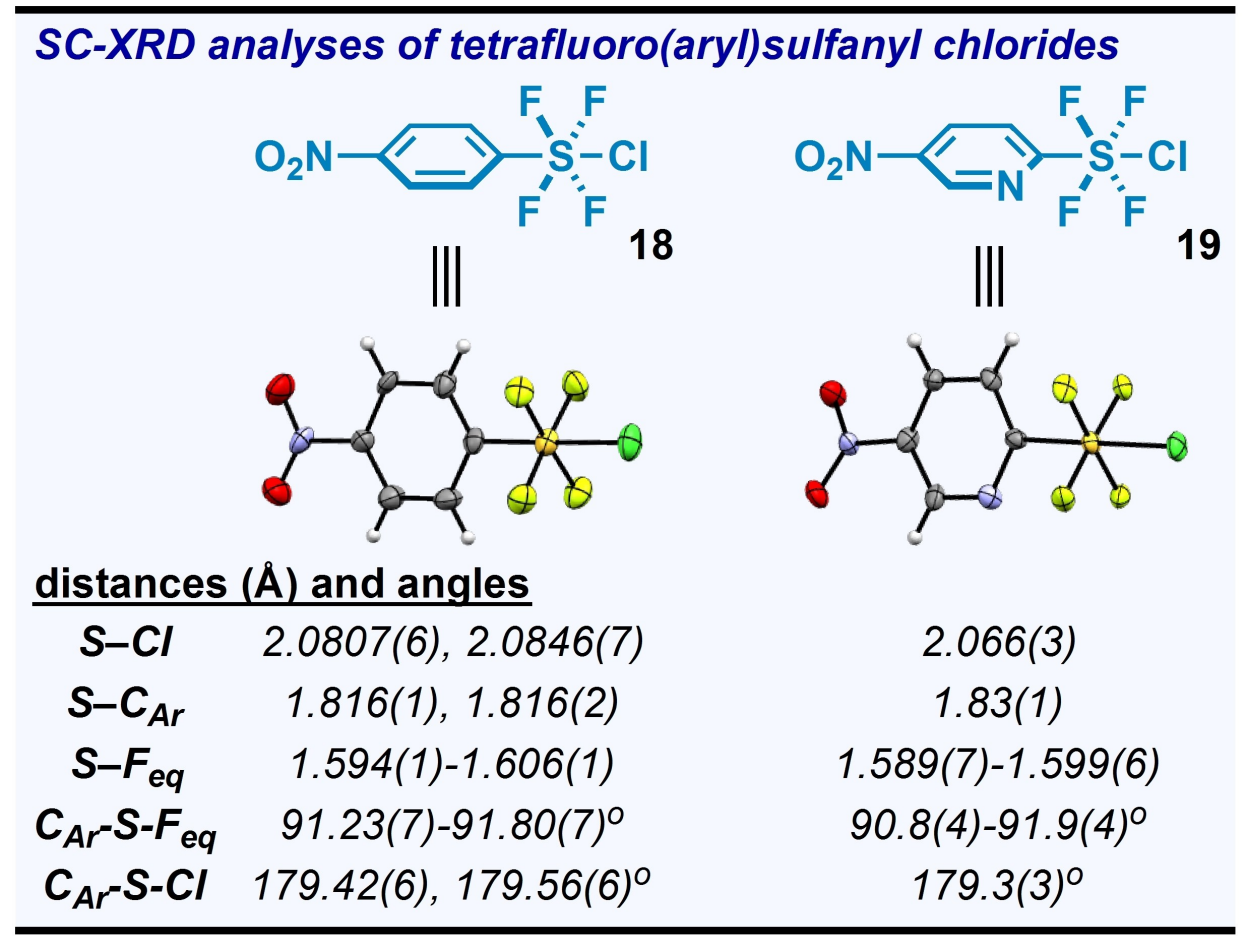
Structures of Ar−SF_4_Cl starting materials determined by single‐crystal X‐ray diffraction (displacement ellipsoids depicted at 50 % probability). The unit cell for **18** contains two symmetry‐independent moieties (only one shown).

Additionally, we managed to obtain and analyze SC‐XRD data for meta‐stable Ar−SF_4_Cl compounds **18** and **19**—the starting materials for **8** and **15**, respectively (Figure 4). When compared alongside the only other two Ar−SF_4_Cl compounds reported in the solid state,^[24]^ they seem to also follow this trend of increasing S−C_Ar_ distance with electron‐withdrawing effect of the arene (concomitant with a decrease in S−Cl distance).

**Figure 5:**
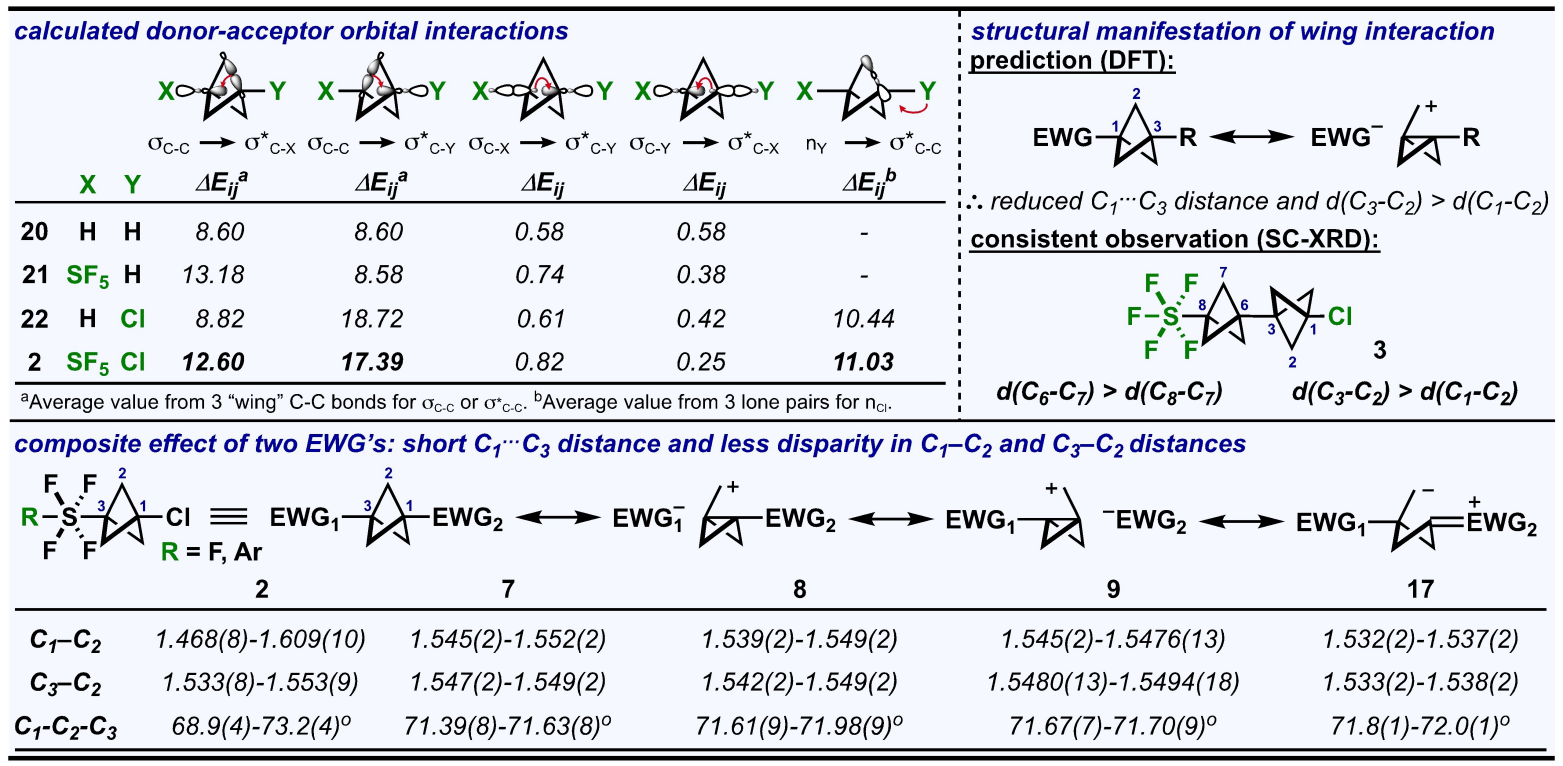
(Top left panel) Comparative analysis of significant donor‐acceptor interactions. Geometry optimizations and NBO analyses conducted at ωB97XD/cc‐pVQZ. Δ*E*_ij_ (in kcal mol^−1^) is the two‐electron stabilization energy associated with delocalization between donor (i) and acceptor (j) NBOs. (Top right panel) Observations in SC‐XRD data of **3** consistent with predicted structural effects. (Bottom panel) Depiction of composite effects from donor‐acceptor interactions consistent with observed short C_1_⋅⋅⋅C_3_ contacts and less disparity in wing C−C distances in SC‐XRD data for **2**,^[21]^ **7**–**9**, and **17**. Distances reported in angstroms (Å).

Finally, we sought more insight as to *why* the C_1_⋅⋅⋅C_3_ contact is so short in the structures included in this study, especially in the instance of **2**. Broadly, the tendency for σ‐electron withdrawing groups to decrease the C_1_⋅⋅⋅C_3_ distance in BCP rings has been established in the literature and is often attributed to some type of through‐space interaction involving the linear arrangement of atoms about the C_1_−C_3_ axis (e.g., both an attractive “percaudal” interaction^[30]^ and a decrease in e^–^–e^−^ repulsion between rear orbital lobes contribute to this effect).[^11a^, ^31^] It has also come to light that a form of hyperconjugation involving the “wing” C−C bonds may contribute significantly to stabilization (e.g., in studies pertaining to the bicyclo[1.1.1]pent‐1‐yl cation, among others).[^32^, ^33^] To study this systematically, we performed geometry optimization and natural bond orbital (NBO) second‐order perturbation analysis of SF_5_−BCP−Cl (**2**) at ωB97XD/cc‐pVQZ, as well as BCP (**20**), SF_5_−BCP−H (**21**), and Cl–BCP−H (**22**) for comparisons (Figure 5, *top left*).^[34]^

**Figure 6:**
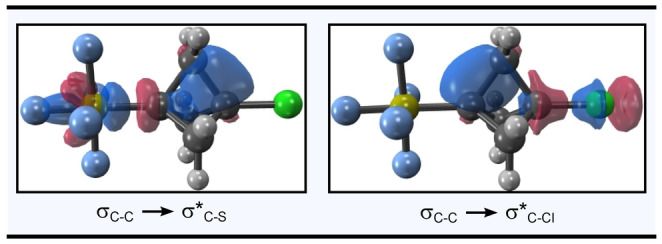
Molecular orbital depictions of donor‐acceptor interactions involving wing C−C bonds in compound **2**. NBOs were generated at ωB97XD/cc‐pVQZ.

The trend in computed C_1_⋅⋅⋅C_3_ distances (as well as *θ*_*C1‐C2‐C3*_ angles) is as expected, following the increase in electron‐withdrawing effect: **2**>**22**≈**21**>**20**. The Wiberg bond index between C_1_ and C_3_ also seems to follow a similar trend, increasing from 0.035 in **20** to 0.085 in **2** (while this trend is noteworthy, these values are too small to argue incipient bond formation).^[35]^ Additionally, by NBO analysis, *the wing C−C bonds contribute a far greater two‐electron stabilization energy* via *a side‐on interaction than donor‐acceptor interactions in the linear array* (Figure 5, *top left*). While computed NBO energies should be carefully considered as only an “upper bound” for actual stabilization energies due to overestimation of electron delocalization effects,^[32]^ the computed wing stabilization energies are over an order of magnitude larger than the percaudal interaction energies in each case. Moreover, the stabilization energies increase when wing C−C bonds are situated across the ring from electron‐withdrawing substituents (**21**, **22**, and **2**), consistent with increased orbital overlap.^[36]^ Representative depictions of these key donor‐acceptor interactions in **2**, resembling an agostic‐like donation from the wing C−C σ‐bond to the σ*_C‐EWG_ orbital, can be found in Figure 6.

Intuitively, a simplified resonance‐like depiction of this type of wing interaction (Figure 5, *top right*) accounts for more “bonding character” between C_1_ and C_3_, and it also suggests there should be a lengthening in the C_3_−C_2_ bond opposite the EWG relative to the C_1_−C_2_ bond.^[37]^ For experimental support, we turned to the X‐ray structure of compound **3**. Here, we see a break in symmetry in both BCP rings and elongation of all wing bonds opposite the EWG's versus the adjacent ones (i.e., d(C_3_−C_2_) and d(C_6_−C_7_) ≈1.56 Å vs. d(C_1_−C_2_) and d(C_8_−C_7_) ≈1.54 Å). While the depiction also suggests elongation of the C–EWG bond, this is complicated for the C−Cl bond, as the stabilization energy derived from Cl lone‐pair donation into wing σ*_C−C_ orbitals is not negligible, and this would conceptually manifest in shortening of the bond (i.e., effects may cancel each other out). However, in the case of the SF_5_ group (devoid of a lone pair), the predicted structural manifestation of this interaction is clearer, as the C−S bond in **3** is longer than in **2** by ≈1.5 % (Figure 2, *bottom*).

It is also apparent that the C_1_⋅⋅⋅C_3_ distance in the X‐ray structure of **3** is approximately equal to the C_6_⋅⋅⋅C_8_ distance (≈1.84 Å), both of which are shorter than the C_1_⋅⋅⋅C_3_ distance in unsubstituted [1.1.1]bicyclopentane.^[23]^ This indicates that a Cl atom and an SF_5_ group have a similar composite effect on shortening the transannular distance in a BCP ring. One can attribute this in large part to the strong electron withdrawing effects of these substituents through the σ‐framework.^[38]^

Lastly, when the bicyclopentane ring is substituted with two σ‐EWG's, a unifying depiction of dominant donor‐acceptor interactions by NBO analysis (Figure 5, *bottom*) also accounts for the further decrease in C_1_⋅⋅⋅C_3_ distance and would suggest that there are competing forces dictating bond elongation/contraction and charge distribution on the wings.^[31b]^ Theoretically, these *push‐pull* effects would not result in the same structural trends observed for compounds like **3**; in fact, this was found to be the case experimentally in structures **2**, **7**, **8**, **9**, and **17** (Figure 5, *bottom*).

## Conclusion

**Figure 7:**
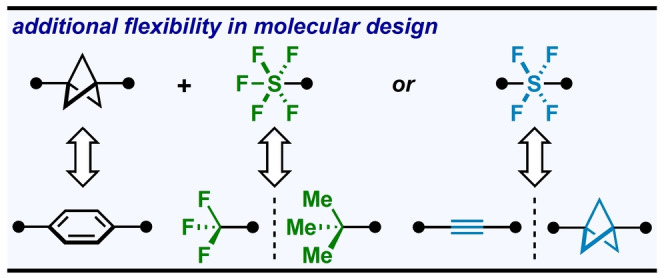
Highlighting the mix‐and‐match design potential associated with hybrid isosteres.

This work was originally motivated by the methodological advancement of forging novel, tertiary C(sp^3^)−SF_5_ and C(sp^3^)−SF_4_Ar bonds through strain‐release functionalization of [1.1.1]propellane. As SF_5_−BCP and –SF_4_−BCP motifs were recognized in this study as types of hybrid isosteres (for [CF_3_/*t*‐Bu+Ph] and [alkyne/BCP+Ph], respectively; see Figure 7), this prompted detailed SC‐XRD studies that led to the discovery and additional investigations of short transannular C⋅⋅⋅C distances. Lastly, DFT calculations and previous literature support the notion that donor‐acceptor interactions involving the wing C−C bonds in BCP rings play a significant role (alongside linear through‐space interactions) in this shortening phenomenon. The theoretical model was corroborated by X‐ray data,^[39]^ whereby manifestations of these “wing donation” effects were observed in solid‐state structures.

We believe these “hybrid isosteres” afford an unusual and subtle type of flexibility in molecular design that may prove useful (Figure 7). Following this critical proof of concept and detailed structural study, future work will pertain to installing SF_5_−BCP and ArSF_4_−BCP motifs onto more complex molecules and evaluating applications.

## Conflict of interest

The authors declare no conflict of interest.

1

## Supporting information

As a service to our authors and readers, this journal provides supporting information supplied by the authors. Such materials are peer reviewed and may be re‐organized for online delivery, but are not copy‐edited or typeset. Technical support issues arising from supporting information (other than missing files) should be addressed to the authors.

Supporting Information

Supporting Information

Supporting Information

Supporting Information

Supporting Information

Supporting Information

Supporting Information

Supporting Information

Supporting Information

Supporting Information

## Data Availability

The data that support the findings of this study are available in the supplementary material of this article.
