# Supplementary material for: Strain‐Release Pentafluorosulfanylation and Tetrafluoro(aryl)sulfanylation of [1.1.1]Propellane: Reactivity and Structural Insight
Source: Angew Chem Int Ed Engl. 2022 Oct 25;61(48):e202211892. doi: 10.1002/anie.202211892 (PMC9828730; doi:10.1002/anie.202211892)
Supplement: Supplementary file 10 — Supporting Information [file ANIE-61-0-s008.pdf]

Angewandte Chemie
Eine Zeitschrift der Gesellschaft Deutscher Chemiker

Supporting Information

Strain-Release Pentafluorosulfanylation and Tetrafluoro(aryl)sulfanylation of [1.1.1]Propellane: Reactivity and Structural Insight

Y. Kraemer, C. Ghiazza, A. N. Ragan, S. Ni, S. Lutz, E. K. Neumann, J. C. Fettinger, N. Nöthling, R. Goddard, J. Cornella, C. R. Pitts**

Contents

General Information **2**

Procedure for the Synthesis of [1.1.1]propellane (*Compound 1*) **2**

Procedure for the Synthesis of SF_5Cl **3**

Procedure for the Synthesis of SF_5-BCP-Cl (*Compound 2*) **4**

General Procedure for the Synthesis of Ar-SF_4-BCP-Cl **5**

Procedures for the Syntheses of Starting Materials **6**

Optimization Tables **8**

Characterization Data **10**

Initial Reactivity Studies of SF_5-BCP-Cl **17**

NMR Spectra **19**

Computational Methods **62**

SC-XRD Data **88**

References **143**

General Information

Unless otherwise stated, all reactions were carried out strictly under anhydrous conditions and under N_2 or Ar atmosphere. Reaction solvents were distilled over CaH_2 or obtained from a solvent purification system directly before use. All NMR data were collected on either 300, 400, or 600 MHz spectrometer. For ^{19}F NMR yield determination, α,α,α-trifluorotoluene (or fluorobenzene) in $CDCl_3$ was introduced after each reaction as an internal standard, and the d1 relaxation delay was increased to 20 s during data collection. The ^{1}H, ^{13}C, and ^{19}F NMR chemical shifts are given in parts per million (δ) and calibrated to either residual solvent signal (^{1}H and ^{13}C), α,α,α-trifluorotoluene (δ = -62.61 ppm in $CDCl_3$), or fluorobenzene (δ = -112.96 in $CDCl_3$).[1a,b] NMR data are reported in the following fashion: chemical shift (integration, multiplicity (s = singlet, d = doublet, quint = quintet, dt = doublet of triplets, dd = doublet of doublets, m = multiplet), coupling constants (Hz)). IR data were collected on a Bruker Tensor 27 FT-IR spectrometer with ATR-IR attachment. HRMS data was collected on a Thermo Fisher Scientific Q Exactive GC Orbitrap (DIP measurement performed with Thermo direct inlet controller with isobutene for the chemical ionization). Additional mass spectrometry experiments were performed on a Bruker timsTOF flex matrix-assisted laser desorption/ionization system. Isotopic distributions were modeled using Bruker Compass Isotope Pattern software. Data was acquired in negative ion mode using 1,5-diaminonaphthalene. Additional matrices and polarities were tried but unsuccessful. Other mass spectrometry experiments included GC-MS and ESI-MS but were unsuccessful, preventing HRMS from being performed on all samples. In general, mass spectrometry-based experiments were difficult to perform/the molecular species was reactive within the gas phase, but all acquired results were consistent the presented structure. The disulfide starting materials for compounds **6**, **8**, and **15** were obtained from Sigma-Aldrich or Oakwood Chemicals. The thiol and disulfide starting materials for compounds **7**, **9**, **10**, **11**, **12**, **13**, **14**, **16**, and **17** were synthesized according to literature protocols,[2a-d] which are provided herein for convenience. The $ArSF_4Cl$ starting materials were synthesized according to literature protocols. PFA vessels for the isolation of $ArSF_4Cl$ compounds were purchased from Savillex.

Procedure for the Synthesis of [1.1.1]propellane (*Compound 1*)[3]

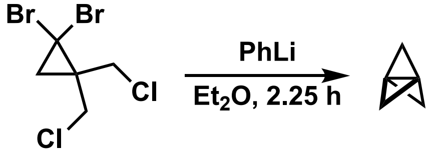

A heat-gun dried 100-mL two-neck round bottom flask was charged with a stir bar and 1,1-dibromo-2,2-bis(chloromethyl)cyclopropane (5.94 g, 10 mmol, 1.0 equiv.). The flask was evacuated and backfilled with N_2. Next, 20 mL of anhydrous Et_2O was added via syringe. The solution was cooled to –45 °C. Phenyllithium (PhLi) (1.8 M in *n*-Bu_2O, 22 mL, 40 mmol, 2.0 equiv.) was added dropwise. Upon addition of PhLi, the reaction mixture turned brown, and a precipitate formed. The reaction was allowed to stir at –45 °C for 20 min and was then transferred to an ice bath and stirred at 0 °C for 2 h. After 2 h, a short path condenser connected to a 100 mL 2-neck receiver flask was introduced. The system was placed under vacuum for 2 min, then static vacuum was maintained. (*Care should be taken not to pull the crude solution over through the distillation head - vigorous bubbling was observed*) Vacuum may be reapplied periodically to

increase the rate of the distillation. After the distillation is complete, the resulting solution of [1.1.1]propellane was transferred via cannula to a Teflon-capped 25 mL Schlenk tube. (*It should be noted that the solution of propellane could be stored over 3 Å molecular sieves in a –20 °C freezer for multiple weeks without noticeable decomposition)*.

For the synthesis of **2** specifically, MeLi (1.6 M in Et_2O) was preferred to PhLi to prepare [1.1.1]propellane. Indeed, Bu_2O appeared to be impossible to remove from the crude mixture given the volatility of the fluorinated product. The rest of the procedure remains the same.

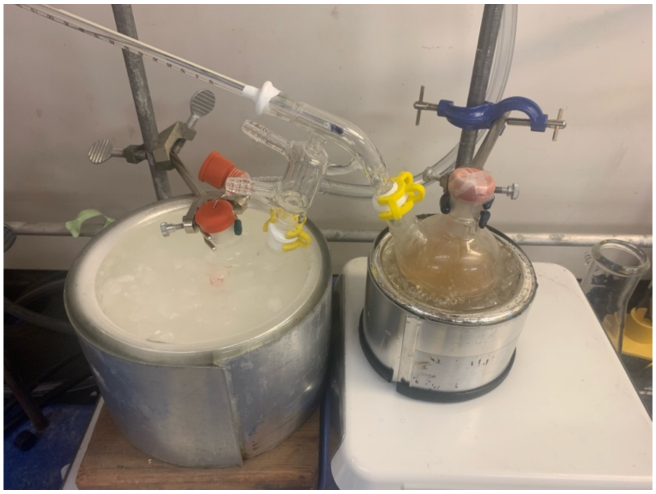

Set up for distillation of propellane

Concentration determination:

A standard solution was prepared by dissolving 1,3,5-trimethoxybenzene (10 – 20 mg) in 0.5 mL anhydrous $CDCl_3$.

Then, 0.1 mL of a standard solution was added to a 3 mm NMR tube. Then, 0.3 mL anhydrous $CDCl_3$ was added, followed by a 0.2 mL aliquot of the [1.1.1]propellane solution. During data collection the d1 relaxation delay was set to 10 s, with 8 scans and 0 dummy scans.

Procedure for the Synthesis of SF_5Cl[4a,b]

Trichloroisocyanuric acid (9.60 g, 4.5 equiv.) was added to a flame-dried 100 mL pressure flask equipped with a stir bar under Ar. Then, spray-dried potassium fluoride (4.84 g, 9.0 equiv.) was added, followed by sulfur (297 mg, 1.0 equiv.). MeCN (46 mL) was added via syringe and the reaction vessel was tightly capped, wrapped in aluminum foil, and transported to the fume hood where it was allowed to stir behind a blast shield at room temperature for 48 h. After completion, the reaction vessel was submerged in a –78 °C dry ice acetone bath. When the solution reached –78 °C, anhydrous hexanes (or *n*-pentane, 29 mL) was added quickly via syringe, while avoiding outgassing. The reaction vessel was then tightly sealed and allowed to warm to room temperature while stirring. Once the mixture reached room temperature, the solution was transferred to an oven-dried separatory funnel. The MeCN layer was discarded and the hexanes (or *n*-pentane) solution containing SF_5Cl was stored in a Schlenk flask under Argon and wrapped in aluminum foil.

Concentration determination:

A standard solution was prepared by dissolving α,α,α-trifluorotoluene (10-20 mg) in 0.5 mL anhydrous $CDCl_3$ under N_2 or Ar atmosphere.

A 3 mm NMR tube was sealed with a septum, wrapped in parafilm, and dried with a heat-gun under vacuum. The tube was backfilled with Ar, then 0.1 mL of a standard solution was added to the tube. Then, 0.3 mL dry $CDCl_3$ was added followed by a 0.2 mL aliquot of the SF_5Cl solution. During data collection, the d1 relaxation delay was set to 20 s, with 8 scans and 0 dummy scans.

Procedure for the Synthesis of SF_5-BCP-Cl (*Compound 2*)

A flame-dried 8 mL screw-capped tube was charged with [1.1.1]propellane in Et_2O (1.0 equiv.). A solution of SF_5Cl in *n*-pentane (1.5 equiv.) was added and the mixture was stirred under white LED irradiation at approx. 30 °C (estimated temperature based on proximity to the light source, ca. 2 cm) for 4 h. Purification of the final product was achieved via distillation:

Stage 1:

The crude reaction mixture was loaded into a 10 mL round bottom flask (bulb 1), and Et_2O and *n*-pentane were removed at room temperature under atmospheric pressure.

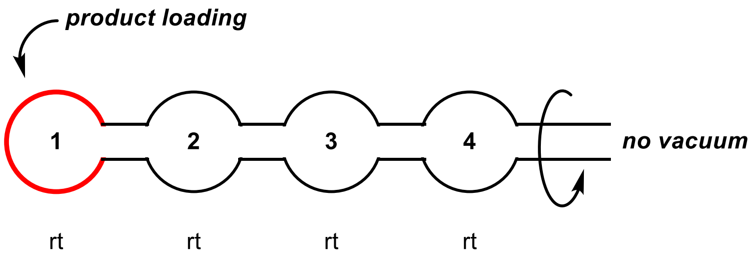

Stage 2:

After the residual solvent was removed, bulbs 3 and 4 were cooled to –78 °C and light vacuum was applied. The vacuum strength was gradually increased until product was carried over to bulb 3. The distillation was then carried out for an additional 15-20 min. The system was carefully warmed to room temperature; the product was dissolved and collected with dry $CDCl_3$.

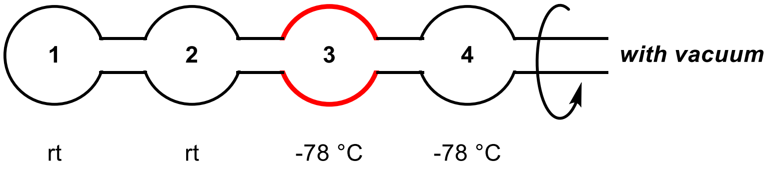

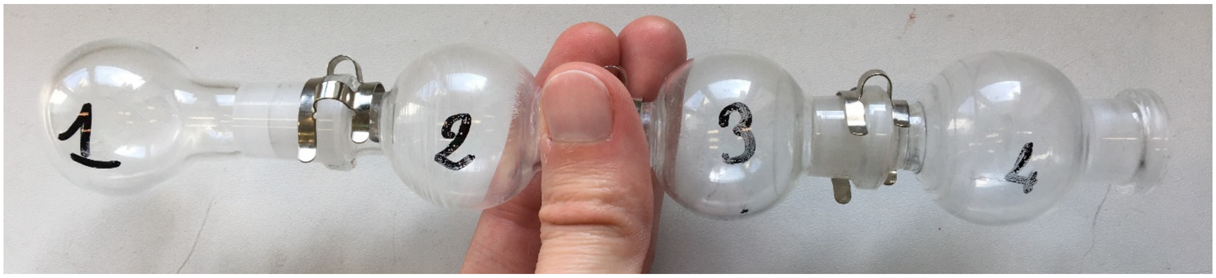

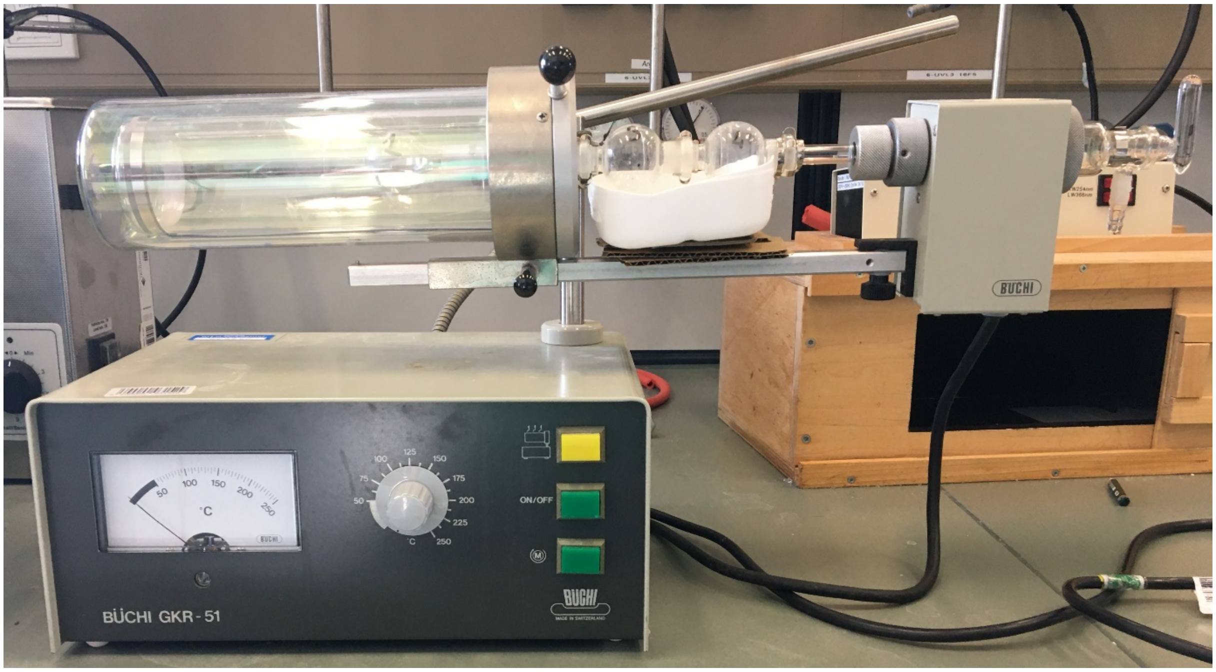

Distillation set up for the purification of compound 2

General Procedure for the Synthesis of Ar-SF$_4$-BCP-Cl

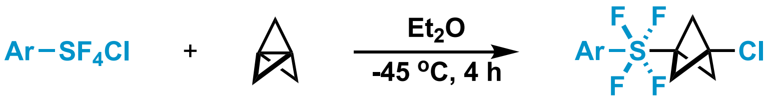

An oven-dried microwave vial equipped with a stir bar was introduced to the glovebox, and the (chlorotetrafluoro-λ^6-sulfanyl)arene was added (0.1 mmol. 1.0 equiv.); the vial was sealed with a crimper. The reaction vessel was removed from the glovebox, wrapped in aluminum foil, and anhydrous Et_2O (0.76 mL) was added. The solution was cooled to –45 °C in an acetonitrile dry ice bath; a solution of [1.1.1]propellane (0.5 M in Et_2O, 0.24 mL, 1.2 equiv.) was added in one portion down the side of the reaction vessel. The reaction was then transferred to an ice bath and stirred for 3 h at 0 °C. The crude reaction mixture was then concentrated in vacuo. The final product was isolated by column chromatography with hexanes and ethyl acetate. (Solvent systems for individual compounds are given below). *Note: the concentration of the [1.1.1]propellane solution was variable between batches; the necessary volume of this solution was calculated and Et_2O was added to give a concentration of 0.1 M with respect to the limiting reagent.*

Procedures for the Syntheses of Starting Materials

*General Method A for Thiol Starting Material Synthesis (compounds **12, 14, 16, 17**)*[2a]

To a heat-gun dried two-neck round bottom flask equipped with a stir bar was added the aryl halide (5.2 mmol, 1.0 equiv.) and thiourea (10.4 mmol, 2.0 equiv.). Subsequently, 12 mL of 200 proof EtOH was added. The reaction was stirred at reflux for 18 h. Then, aqueous sodium hydroxide (13.8mmol, 2.7 equiv.) was added and the reaction was allowed to reflux for an additional 2 h. After completion of the reaction, EtOH was removed under a stream of nitrogen. A 1.0 M HCl solution was added dropwise until precipitation of the yellow product ceased. The resulting solid was filtered and washed with H_2O. The product was allowed to dry and was carried forward without further purification.

*General Method B for Thiol Starting Material Synthesis (compounds **9, 10**)*[2b]

The aryl halide substrate (8.9 mmol, 1.0 equiv.) and sodium hydrosulfide hydrate (10.7 mmol. 1.2 equiv.) were added to a 2-neck 50 mL round bottom flask equipped with a stir bar, followed by 13 mL of DMF. This mixture was then stirred for 18 h at 70 °C. Upon completion, the reaction was diluted with water (10 mL) and extracted into EtOAc (5 × 10 mL). The combined organic layers were dried over $MgSO_4$, filtered through Celite, and concentrated in vacuo. The resulting material was carried forward without any further purification.

*General Method A for Disulfide Starting Material Synthesis (compounds **9, 10, 12, 14, 16, 17**)*[2a]

To a round bottom equipped with a stir bar was added the thiol starting material (6.8 mmol, 1.0 equiv.) suspended in 30 mL H_2O. NaOH (0.27g, 6.8 mmol, 1.0 equiv.) was then added and the reaction was stirred for 30 min at room temperature (ca. 23 °C). $K_3[Fe(CN)_6]$ (2.7g, 8.2 mmol, 1.2 equiv.) was added and the reaction was allowed to stir at room temperature for 22 h, at which point a precipitate had formed. The crude reaction mixture was filtered. The resulting off-white solid was then dissolved in CH_2Cl_2, dried over $MgSO_4$, and concentrated in vacuo. The resulting material was carried forward without any further purification.

General Method B for Disulfide Starting Material Synthesis (compound 7)[2c]

The thiophenol starting material (1 mmol, 1.0 equiv.) was added to a round bottom flask equipped with a stir bar; a mixture of 5:1 MeCN:H_2O (3 mL) was added. Iodine (0.5 mmol, 1.0 equiv.) was added, and the reaction was stirred for 2 h at room temperature (ca. 23 °C). The reaction was quenched with 3 mL 1 % aqueous sodium thiosulfate and extracted into CH_2Cl_2. The organic layers were combined, dried with $MgSO_4$, and concentrated in vacuo. The desired disulfide products were obtained as off-white solids and were used without further purification.

*Procedure for the Synthesis of Disulfide Starting Material for Compound **11***[2d]

5-bromo-2-fluoropyridine (1.76 g, 10 mmol, 1.0 equiv.) was added to a heat-gun dried 3-neck 250 mL round bottom flask equipped with a stir bar under Ar atmosphere, followed by 25 mL THF. The mixture was cooled to –45 °C in a dry-ice acetonitrile bath; *i*-PrMgCl-LiCl (10 mL, 11 mmol, 1.1 equiv.) was added dropwise. The mixture was allowed to warm to room temperature and stirred

for 2 h (ca. 23 °C). Elemental sulfur (352 mg, 11 mmol, 1.1 equiv.) was dissolved in anhydrous toluene (35 mL); this mixture was added to the reaction mixture dropwise at –45 °C via cannula. The reaction was allowed to warm to room temperature and stirred for an additional hour. The reaction was then poured into a 250 mL round bottom flask containing a stir bar, KOH (1.16 g, 20 mmol, 2.0 equiv.), and $K_3[Fe(CN)_6]$ (3.59 g, 30 mmol, 3.0 equiv.) in H_2O (50 mL). The mixture was allowed to stir at room temperature overnight. Upon completion an off-white precipitate had formed. The reaction was filtered through a pad of Celite; the filtrate was then extracted with CH_2Cl_2 (3 × 15 mL). The combined organic layers were dried over $MgSO_4$ and concentrated in vacuo. The crude residue was purified via column chromatography (9:1, hexanes:EtOAc).

Procedure for the Synthesis of Disulfide Starting Material for Compound ***13***[2a]

6,6'-Dithionicotinic acid (2.0 g, 4.22 mmol, 1.0 equiv.) was added to a heat-gun dried round bottom flask under Ar. Anhydrous CH_2Cl_2 (21 mL) was added, followed by methanol (2.38 mL, 9.23 mmol, 2.2 equiv.), N,N′-Diisopropylcarbodiimide (1.32 mL, 8.43 mmol, 2.0 equiv.), and 4-dimethylaminopyridine (103 mg, 0.84 mmol, 0.2 equiv.). The reaction was allowed to stir at room temperature (ca. 23 °C) for 18 h. Upon completion, the precipitate was removed via filtration through Celite. The crude mixture was concentrated in vacuo and purified via column chromatography (8:2 hexanes:EtOAc). The desired disulfide was obtained as an off-white solid. NMR spectra matched literature values.

General procedure of Synthesis of Aryl Tetrafluoro- λ^6-sulfanyl Chlorides[2a]

Trichloroisocyanuric acid (18.0 equiv.) was added to an oven-dried round bottom flask charged with a stir bar under glovebox atmosphere. Then, spray-dried potassium fluoride (32.0 equiv.) was added, followed by the disulfide starting material (1.0 equiv.). Acetonitrile was added to the reaction flask followed by a solution of trifluoroacetic acid (0.1 equiv.) in acetonitrile. The reaction was stirred under glovebox atmosphere at room temperature (ca. 23 °C) for 18 h. Upon completion of the reaction, TCICA and KF were removed by filtration of the solvent through a PTFE syringe filter into a PFA vessel; the solvent was then removed in vacuo. The left-over white solids were extracted with *n*-pentane (or hexanes), filtered through a PTFE syringe filter into a PFA vessel and concentrated in vacuo.

Optimization Tables

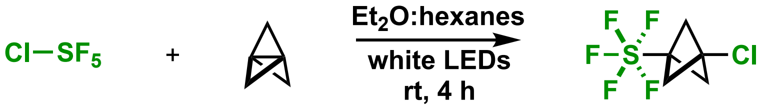

Table S1. Screening Conditions for SF_5-BCP-Cl Synthesis.

Entry	Conditions	Temperature (°C)	^{19}F yield DP **2** (%)	^{19}F yield dimer **3** (%)	Remaining $ClSF_5$ (%)
1	dark	25	35	3	16
2	Ambient light	25	32	3	23
3	dark	–45 to 0	20	3	15
4	CFL lamp, 14 W	25	50	4	13
5	**White LED, 20 W**	**25**	**85**	**9**	**5**
6	Blue LED (strips), 14.4 W	25	43	8	—
7	2 UV lamps, 390 nm, 50 W	25	24	16	—
8	BEt_3 10%, air, dark	–45 to 0	48	2	—
9	BEt_3 10%, dark	–45 to 0	16	2	—
10*	**White LED, 20 W**	**25**	**89**	**7**	**14**

* SF_5Cl (0.095 M in *n*-pentane)

General procedure: To a flame-dried 8 mL screw-capped tube under Ar were added [1.1.1]propellane (0.28 M in Et_2O, 350 µL, 0.098 mmol, 1.0 equiv.), SF_5Cl (0.075 M in *n*-pentane, 2.0 mL, 0.15 mmol, 1.5 equiv.) and a potential additive (1.0 M BEt_3 in hexanes 10 µL, 0.01 mmol, 10 mol%). The mixture was stirred for 4 h under specified conditions. For photochemical reactions, the reaction vessel was placed ca. 5 cm from the light source(s). Additionally, for UV light irradiation, a fan was used to maintain the temperature around 25 °C.

Deviation from the standard conditions: Reaction 7 in Table S1 was conducted in a 2 mL vial and the reaction was performed on 1/2 the scale. Reaction 8 was conducted under normal atmosphere.

After the specified time, $PhCF_3$ was added to the mixture. An aliquot of 0.1 mL was taken and diluted with 0.3 mL $CDCl_3$. Yields were determined by ^{19}F NMR.

See Figure S2 for the ^{19}F NMR analysis of the crude mixture from Entry 10.

Table S2. Screening Conditions for $ArSF_4$-BCP-Cl Synthesis.

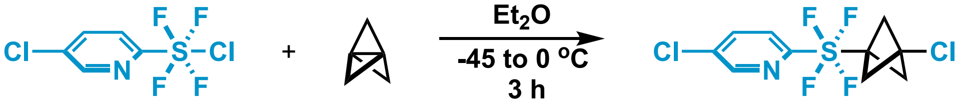

Entry	Equiv. $ArSF_4Cl$	Equiv. Propellane	mmol $ArSF_4Cl$	Initiator	Solvent	Temperature (°C)	Additive (1.5 equiv.)	^{19}F NMR yield (%)
1	1.2	1.0	0.01	—	Et_2O	–78	—	14
2	1.2	1.0	0.01	—	Et_2O	–45	—	8
3	1.2	1.0	0.01	—	Et_2O	–78 to 0	—	11
4	1.2	1.0	0.01	—	Et_2O	–45 to 0	—	17
5	1.2	1.0	0.01	—	Et_2O	0	—	11
6	1.2	1.0	0.01	—	Et_2O	rt (~23)	—	14
7	1.2	1.0	0.01	BEt_3	Et_2O	–45 to 0	—	16
8	1.2	1.0	0.01	dark	Et_2O	–45 to 0	—	45
9	1.2	1.0	0.01	White LED[a]	Et_2O	–45 to 0	—	8
10	1.2	1.0	0.01	Blue LED[b]	Et_2O	–45 to 0	—	19
11	1.2	1.0	0.01	dark	MeCN	–45 to 0	—	25
12	1.2	1.0	0.01	dark	THF	–45 to 0	—	35
13	1.2	1.0	0.01	dark	hexanes	–45 to 0	—	26
14	1.2	1.0	0.01	dark	CH_2Cl_2	–45 to 0	—	28
15	1.0	1.0	0.01	dark	Et_2O	–45 to 0	—	44
16	2.0	1.0	0.01	dark	Et_2O	–45 to 0	—	49
17	5.0	1.0	0.01	dark	Et_2O	–45 to 0	—	45
18	1.0	1.2	0.01	dark	Et_2O	–45 to 0	—	53
19	1.0	2.0	0.01	dark	Et_2O	–45 to 0	—	43
20	1.0	5.0	0.01	dark	Et_2O	–45 to 0	—	21
21	**1.0**	**1.2**	**0.1**	**dark**	**Et_2O**	**–45 to 0**	**—**	**85**
22	1.0	1.2	0.5	dark	Et_2O	–45 to 0	—	70
23	1.0	1.2	0.1	dark	Et_2O	–45 to 0	CuCl	16
24	1.0	1.2	0.1	dark	Et_2O	–45 to 0	Li_2CO_3	64
25	1.0	1.2	0.1	dark	Et_2O	–45 to 0	K_3PO_4	30
26	1.0	1.2	0.1	dark	Et_2O	–45 to 0	$NaHCO_3$	35
27	1.0	1.2	0.1	dark	Et_2O	–45 to 0	4 Å mol. sieves	55

[a] 72 W Coleman 72 LED Rechargeable Task Light ("cool white" LEDs).
[b] 40 W Kessil A160WE Tuna Blue LED Light.

Characterization Data

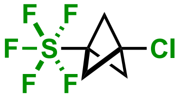

Compound 2. The reaction was run according to the general procedure. The final compound was isolated as a colorless liquid (86%). Crystals suitable for SC-XRD were obtained by progressive cooling of the neat liquid to –60 °C in a dry-ice acetone bath.

^{1}H NMR (600 MHz, CDCl$_3$): δ = 2.65 (s, 6H).
^{19}F NMR (565 MHz, CDCl$_3$): δ = 78.65-77.60 (m, 1F), 54.54 (d, 4F, $^2J_{F,F}$ = 148.1 Hz).
^{13}C{^{1}H} NMR (151 MHz, CDCl$_3$): δ = 63.58 (m, C–SF$_5$), 60.52 (quint, $^2J_{C,F}$ = 2.4 Hz, 3CH$_2$), 45.07 (bs, C–Cl).
HRMS: (DIP-CI) for [C$_5$H$_6$SClF$_5$-H]$^-$: calculated 226.971518, measured 226.971100.

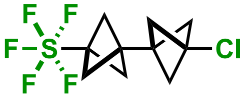

Compound 3. The final compound was isolated as a white solid (7 %) as a byproduct of the synthesis of compound 2. Crystals suitable for SC-XRD were obtained by slow solvent evaporation from the crude mixture.

^{1}H NMR (300 MHz, CDCl$_3$): δ = 2.19 (s, 6H), 2.05 (s, 6H).
^{19}F NMR (565 MHz, CDCl$_3$): δ = 80.17 – 78.10 (m, 1F), 48.49 (d, 4F, $^2J_{F,F}$ = 145.8 Hz).

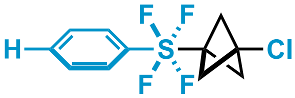

Compound 6. The reaction was run according to the general procedure. The final compound was isolated as a white crystalline solid (41 %) after elution with 9:1 to 8:1 hexanes:EtOAc.

R$_f$: 0.26 (9:1 hexanes:EtOAc)
^{1}H NMR (300 MHz, CDCl$_3$): δ = 7.84-7.57 (m, 5H), 2.38 (s, 6H).
^{19}F (282 MHz, CDCl$_3$): δ = 68.6 (s, 4F).
^{13}C{^{1}H} (101 MHz, CDCl$_3$): δ = 136.8, 134.3, 129.5, 128.5, 57.1, 49.7, 48.5.
ATR-IR: 1586, 721, 687, 668 cm^{-1}.
MS: Theoretical *m/z* 284.0, measured *m/z* 284.1 for molecular ion. Isotopic distribution consistent with a chlorinated species.

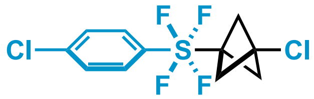

Compound 7. The reaction was run according to the general procedure. The final compound was isolated as a white crystalline solid (56 %) after elution with 9:1 hexanes:EtOAc. Crystals suitable for SC-XRD were grown by solvent evaporation from EtOAc.

R_f: 0.25 (9:1 hexanes:EtOAc)
^{1}H NMR (300 MHz, $CDCl_3$): δ = 7.67-7.62 (2H, dt, *J* = 2.96, 9.1 Hz), 7.36-7.33 (2H, d, *J* = 8.72 Hz, 2.68 (6H, s).
^{19}F (282 MHz, $CDCl_3$): δ = +55.6 (4F, s)
^{13}C{^{1}H} (101 MHz, $CDCl_3$): δ = 158.9-157.5 (quint, $^2J_{C\text{-}F}$ = 23.5 Hz), 136.4, 128.4, 127.8-127.5 (quint, $^3J_{C\text{-}F}$ = 5.3 Hz), 69.6-68.1 (quint, $^2J_{C\text{-}F}$ = 29.0 Hz), 61.3-61.2 (quint, $^4J_{C\text{-}F}$ = 2.7 Hz), 45.43-45.38 (quint, $^4J_{C\text{-}F}$ = 1.9 Hz).
ATR-IR: 1574, 751, 736, 681 cm^{-1}.
MS: Molecular ion not detected. Isotopic distribution of fragmented species consistent with a chlorinated molecular ion.

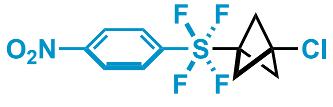

Compound 8. The reaction was run according to the general procedure. The final compound was isolated as a white crystalline solid (63 %) after elution with 95:5 hexanes:EtOAc. Crystals suitable for SC-XRD were grown by solvent evaporation from EtOAc.

R_f: 0.21 (9:1 hexanes:EtOAc)
^{1}H NMR (300 MHz, $CDCl_3$): δ = 8.24-8.22 (2H, d, *J* = 8.9 Hz), 7.90-7.88 (2H, dt, 2.3, 9.2 Hz), 2.70 (6H, s).
^{19}F (282 MHz, $CDCl_3$): δ = +55.9 (4F, s)
^{13}C{^{1}H} (151 MHz, $CDCl_3$): δ = 163.9-163.2 (quint, $^2J_{C\text{-}F}$ = 26.6 Hz), 148.3, 127.7-127.5 (quint, $^2J_{C\text{-}F}$ = 5.5 Hz), 123.7, 69.0-68.2 (quint, $^2J_{C\text{-}F}$ = 27.6 Hz), 61.2, 45.2.
ATR-IR: 1611, 1528, 1353, 756, 729, 669 cm^{-1}.
MS: Theoretical *m/z* 375.0, measured *m/z* 375.1 for molecular species adducted to solvent. Isotopic distribution of fragmented species consistent with a chlorinated molecular ion.

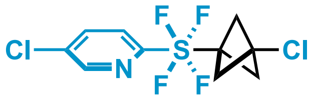

Compound 9. The reaction was run according to the general procedure. The final compound was isolated as a white crystalline solid (72 %) after elution with 9:1 hexanes:EtOAc. Crystals suitable for SC-XRD were grown by vapor diffusion. The isolated compound was dissolved in a minimum amount of EtOAc, and hexanes was added as the counter solvent.

R$_f$: 0.31 (9:1, hexanes:EtOAc).
^{1}H NMR (300 MHz, CDCl$_3$): δ = 8.44-8.43 (1H, d, J = 2.3 Hz), 7.81-7.76 (1H, d, J = 8.7 Hz), 7.66-7.63 (1H, d, J = 8.7 Hz), 2.70 (6H, s).
^{19}F (282 MHz, CDCl$_3$): δ = +46.5 (4F, s)
^{13}C{^{1}H} (101 MHz, CDCl$_3$): δ = 168.0-167.2 (quint, $^2J_{C\text{-}F}$ = 31.6 Hz), 146.12-146.06 (m, $^3J_{C\text{-}F}$ = 2.1 Hz), 137.99, 134.1, 122.6-122.3 (quint, $^3J_{C\text{-}F}$ = 4.6 Hz), 68.1-67.4 (quint, $^2J_{C\text{-}F}$ = 27.0 Hz), 61.2-61.1 (quint, $^4J_{C\text{-}F}$ = 2.7 Hz), 45.4.
ATR-IR: 1571, 1560, 751, 734, 692 cm^{-1}.
MS: Theoretical *m/z* 337.0, measured *m/z* 337.1 for hydrated molecular species. Isotopic distribution consistent with a chlorinated species.

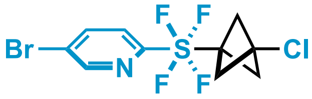

Compound 10. The reaction was run according to the general procedure. The final compound was isolated as a white crystalline solid (71 %) after elution with 8:2 hexanes:EtOAc.

R$_f$: 0.15 (9:1, hexanes:EtOAc).
^{1}H NMR (300 MHz, CDCl$_3$): δ = 8.54-8.53 (1H, d, J = 2.2 Hz), 7.95-7.92 (1H, d, J = 8.3 Hz), 7.59-7.56 (1H, d, J = 8.7 Hz), 2.69 (6H, s).
^{19}F (282 MHz, CDCl$_3$): δ = +47.1 (4F, s).
^{13}C{^{1}H} (101 MHz, CDCl$_3$): δ = 151.8, 141.0, 126.0, 124.4, 61.1, 58.0, 48.6.
ATR-IR: 1567, 1553, 779, 760, 628 cm^{-1}.
MS: Molecular ion not detected. Isotopic distribution of fragmented species consistent with a chlorinated and brominated molecular ion.

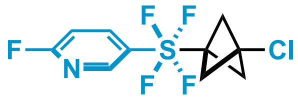

Compound 11. The reaction was run according to the general procedure. The final compound was isolated as a white crystalline solid (41 %) after elution with 95:5 to 8:2 hexanes:EtOAc.

R_f: 0.46 (9:1, hexanes:EtOAc).
^{1}H NMR (300 MHz, $CDCl_3$): δ = 8.58-8.57 (1H, d, J = 1.6 Hz), δ = 8.13-8.06 (1H, m, J = 2.2, 7.9 Hz), 6.96-6.92 (1H, dd, J = 2.9, 8.9 Hz), 2.69 (6H, s).
^{19}F (282 MHz, $CDCl_3$): δ = +47.1 (4F, s), -64.5 (1F, s).
^{13}C{^{1}H} (151 MHz, $CDCl_3$): δ = 145.9, 139.4, 109.4, 109.2, 68.8, 61.2, 45.1.
ATR-IR: 1589, 1575, 749, 688 cm^{-1}.
MS: Theoretical *m/z* 326.0, measured *m/z* 326.1 for molecular species. Isotopic distribution consistent with a chlorinated species.

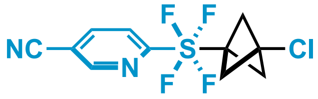

Compound 12. The reaction was run according to the general procedure. The final compound was isolated as a white crystalline solid (65 %) after elution with 9:1 hexanes:EtOAc.

R_f: 0.31 (9:1, hexanes:EtOAc).
^{1}H NMR (300 MHz, $CDCl_3$): δ = 8.79-8.78 (1H, d, J = 1.7 Hz), 8.13-8.11 (1H, d, J = 8.5 Hz), 7.84-7.82 (1H, d, J = 8.5 Hz), 2.71 (6H, s).
^{19}F (282 MHz, $CDCl_3$): δ = +46.5 (4F, s).
^{13}C{^{1}H} (151 MHz, $CDCl_3$): δ = 171.5-171.0 (quint, $^2J_{C\text{-}F}$ = 32.9 Hz), 150.5, 141.8, 122.04-121.98 (quint, $^3J_{C\text{-}F}$ = 4.4 Hz), 115.3, 112.3, 67.8-67.4 (quint, $^2J_{C\text{-}F}$ = 26.0 Hz), 61.1, 45.1.
ATR-IR: 2243, 1584, 1562, 759, 739, 707 cm^{-1}.
MS: Theoretical *m/z* 312.0, measured *m/z* 312.0 for molecular species. Isotopic distribution consistent with a chlorinated species.

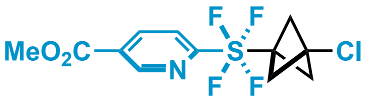

Compound 13. The reaction was run according to the general procedure. The product was isolated as a white crystalline solid (81 %) after elution with 5:1 hexanes:EtOAc. There was evidence for decomposition of the final product on silica via 2D TLC.

R_f: 0.31 (5:1 hexanes:EtOAc).
^{1}H NMR (300 MHz, $CDCl_3$): δ = 9.08 (1H, d, J = 2.2 Hz), 8.43 (1H, d, J = 8.5 Hz), 7.78 (1H, d, J = 8.5 Hz), 3.98 (3H, s), 2.72 (6H, s).
^{19}F (282 MHz, $CDCl_3$): δ = +46.2 (4F, s).
^{13}C{^{1}H} (151 MHz, $CDCl_3$): δ = 172.3-171.9 (quint, $^2J_{C-F}$ = 31.3 Hz), 164.4, 148.9, 139.8, 128.1, 121.4-121.3 (quint, $^3J_{C-F}$ = 4.6 Hz), 61.12-61.09 (quint, $^3J_{C-F}$ = 2.6 Hz), 52.9, 45.3. *Note: the quintet of the alkyl carbon atom adjacent to the -SF_4- unit could not be resolved.*
ATR-IR: 1736, 1726, 1585 (br), 744, 683 cm^{-1}.
MS: Theoretical *m/z* 377.0, measured *m/z* 376.9 for chlorinated molecular species. Isotopic distribution consistent with a chlorinated species.

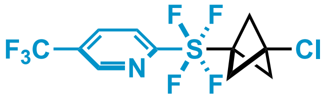

Compound 14. The reaction was run according to the general procedure. The product was isolated as a white crystalline solid (56 %) after elution with 9:1 hexanes:EtOAc.

R_f: 0.21 (9:1 hexanes:EtOAc).
^{1}H NMR (300 MHz, $CDCl_3$): 8.78 (1H, s), 8.10-8.08 (1H, d, J = 8.5 Hz), 7.85-7.83 (1H, d, J = 8.5 Hz), 2.73 (6H, s).
^{19}F (282 MHz, $CDCl_3$): δ = +46.5 (4F, s), -62.5 (3F, s).
^{13}C{^{1}H} (101 MHz, $CDCl_3$): δ = 172.1-171.2 (quint, $^2J_{C-F}$ = 32.3 Hz), 144.9-144.8 (quint, $^2J_{C-F}$ = 2.2 Hz), 135.99-135.95 (quint, $^2J_{C-F}$ = 3.3 Hz), 129.5-128.1 (quint, $^2J_{C-F}$ = 33.8 Hz), 124.5, 121.9-121.7 (quint, $^3J_{C-F}$ = 4.8 Hz), 68.3-66.9 (quint, $^2J_{C-F}$ = 26.7 Hz), 61.2-61.0 (quint, $^3J_{C-F}$ = 2.7 Hz), 45.3.
ATR-IR: 1598, 1580, 750, 735, 692 cm^{-1}.
MS: Theoretical *m/z* 353.0, measured *m/z* 353.0 for molecular species. Isotopic distribution of fragmented species consistent with a chlorinated molecular ion.

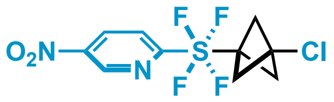

Compound 15. The reaction was run according to the general procedure. The final compound was isolated as a white crystalline solid (45 %) after elution with 9:1 hexanes:EtOAc.

R_f: 0.3 (9:1 hexanes:EtOAc)
^{1}H NMR (300 MHz, $CDCl_3$): δ = 9.54-9.53 (1H, d, *J* = 2.4 Hz), 8.78-8.75 (1H, dd, *J* = 2.4, 8.5 Hz), 8.32-8.29 (1H, d, *J* = 8.5 Hz), 2.60 (6H, s).
^{19}F (282 MHz, $CDCl_3$): δ = +47.36 (4F, s).
^{13}C{^{1}H} (101 MHz, $CDCl_3$): δ = 160.97, 145.7, 133.9, 123.6, 58.2, 48.4. *Note: the quintets of aryl and alkyl carbon atoms adjacent to the -SF_4- unit could not be resolved.*
ATR-IR: 1602, 1568, 1533, 1358, 752, 737, 694 cm^{-1}.
MS: Theoretical *m/z* 376.97, measured *m/z* 376.99 for molecular species adducted to solvent. Isotopic distribution consistent with a chlorinated species.

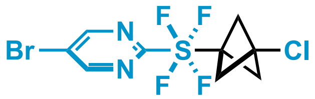

Compound 16. The reaction was run according to the general procedure. The product was isolated as a white crystalline solid (67 %) after elution with 9:1 hexanes:EtOAc.

R_f: 0.29 (9:1 hexanes:EtOAc)
^{1}H NMR (300 MHz, $CDCl_3$): δ = 8.86 (2H, s), 2.71 (6H, s).
^{19}F (282 MHz, $CDCl_3$): δ = +43.61 (4F, s).
^{13}C{^{1}H} (101 MHz, $CDCl_3$): δ = 158.9, 121.84, 61.11-61.06 (quint, $^3J_{C\text{-}F}$ = 2.6 Hz), 31.1. *Note: the quintets of aryl and alkyl carbon atoms adjacent to the -SF_4- unit could not be resolved.*
ATR-IR: 1541(br), 746, 694 cm^{-1}.
MS: Molecular ion not detected. Isotopic distribution of fragmented species consistent with a chlorinated and brominated molecular ion.

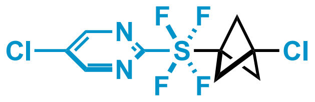

Compound 17. The reaction was run according to the general procedure. The product was isolated as a white crystalline solid (84 %) after elution with 9:1 hexanes:EtOAc. Crystals suitable for SC-XRD were grown by vapor diffusion. The isolated compound was dissolved in a minimum amount of EtOAc, and hexanes was added as the counter solvent.

R_f: 0.29 (9:1 hexanes:EtOAc)
^{1}H NMR (300 MHz, $CDCl_3$): δ = 8.78 (2H, s), 2.73 (6H, s).
^{19}F (282 MHz, $CDCl_3$): δ = +43.64 (4F, s).
^{13}C{^{1}H} (101 MHz, $CDCl_3$): δ = 171.5-170.5 (quint, $^2J_{C\text{-}F}$ = 37.7 Hz), 156.51-156.47 (quint, $^3J_{C\text{-}F}$ = 1.6 Hz), 132.8, 67.2-66.5 (quint, $^2J_{C\text{-}F}$ = 26.1 Hz), 61.0-60.9 (quint, $^3J_{C\text{-}F}$ = 2.7 Hz), 45.3.
ATR-IR: 1567, 1553, 753, 737, 690 cm^{-1}.
MS: Theoretical *m/z* 380.98, measured *m/z* 381.05 for molecular species adducted to solvent. Isotopic distribution of fragmented species consistent with a chlorinated molecular ion.

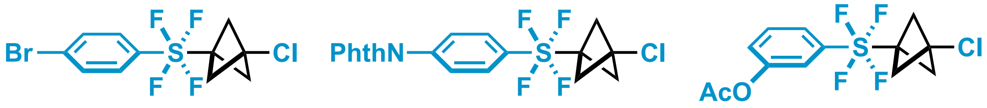

^{19}F (282 MHz, $CDCl_3$) +56.4 ppm +56.3 ppm +46.2 ppm

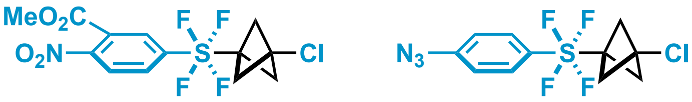

^{19}F (282 MHz, $CDCl_3$) +53.2 ppm +56.7 ppm

Figure S1: Examples of products (not appearing in the manuscript) that are prone to decomposition on silica gel. *Note: purification of (4-bromophenyl)(3-chlorobicyclo[1.1.1]pentan-1-yl)tetrafluoro-λ⁶-sulfane, shown above, was also attempted using basic alumina (7:3 CH_2Cl_2:hexanes), as well as triethylamine-treated silica (90:8:2 hexanes:EtOAc:NEt_3). In each case, evidence for decomposition was observed via 2D TLC analysis and all attempts at isolation via column chromatography resulted in complete decomposition of the $ArSF_4$-BCP-Cl product.*

Initial Reactivity Studies of SF_5-BCP-Cl

To a flame-dried 8 mL screw-capped tube under Ar were added [1.1.1]propellane (solution in Et_2O, 0.10 mmol, 1.0 equiv.) and SF_5Cl (solution in *n*-pentane, 0.15 mmol, 1.5 equiv.). The mixture was stirred for 4 h under irradiation with white LEDs as outlined above.

Note: the concentration of the [1.1.1]propellane and SF_5Cl solutions were variable between batches; the necessary volume of these solutions were calculated for each reaction.

Then, the crude mixture containing compound **2** was carefully concentrated under moderate vacuum (600 mbar, 25 °C, rotary evaporator, air) to the minimum amount of solvent. Anhydrous solvent (1.0 mL) is added to the tube and transferred to another flame-dried 8 mL screw-capped tube under the appropriate atmosphere (Ar or CO_2) containing the reactants. The reaction time as well as the temperature is indicated for each run.

$PhCF_3$ was added to the mixture after the indicated reaction times. An 0.1 mL aliquot was taken from the reaction mixture and diluted with 0.3 mL $CDCl_3$. The crude reaction mixtures were analyzed by ^{19}F NMR.

Note: for metalation reactions and abstractions with Lewis acids, the aliquot was partitioned with water (0.1-0.2 mL) to quench the residual metals.

1) *Metalation*

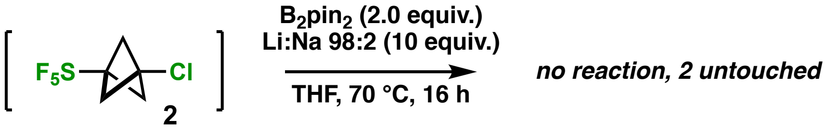

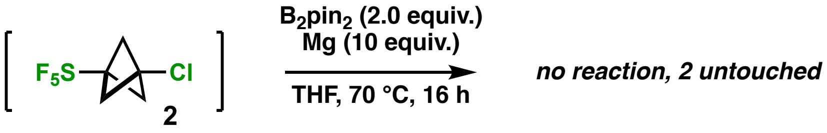

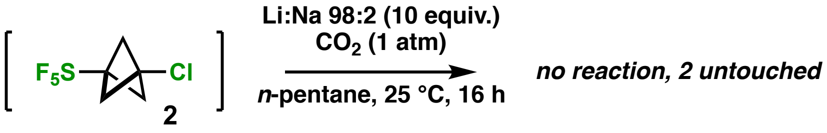

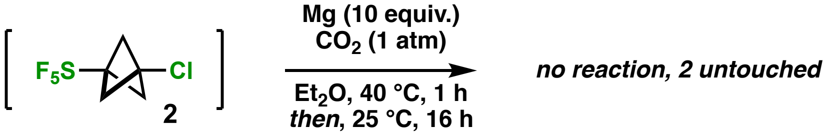

2) Cl Abstraction with Lewis Acids

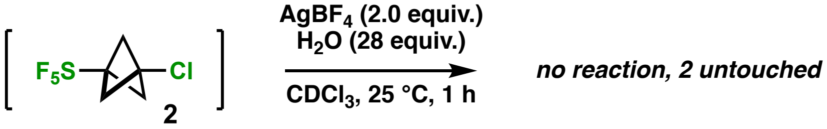

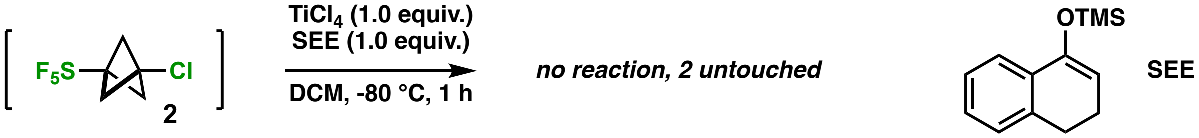

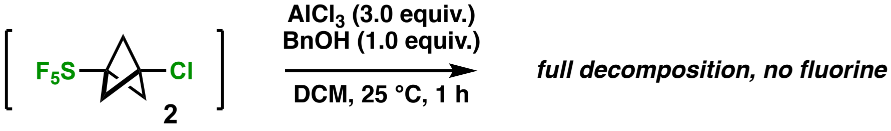

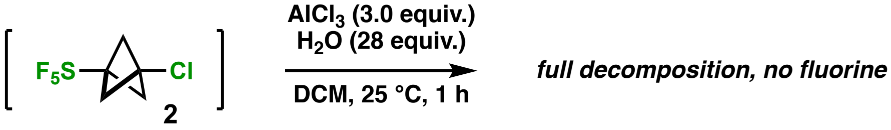

3) Radical Cl Abstraction

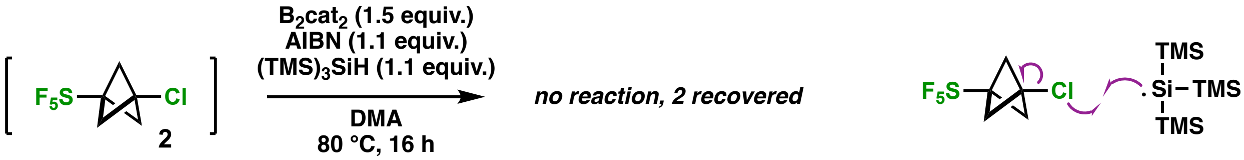

4) C–Cl Bond Reduction

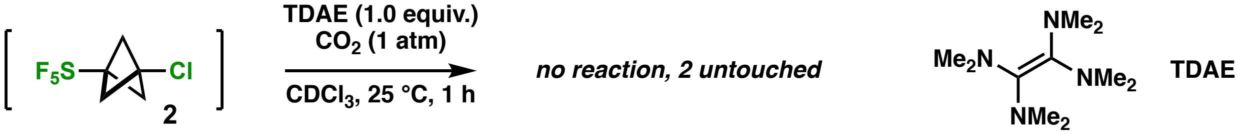

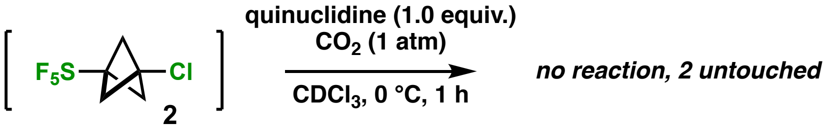

NMR Spectra

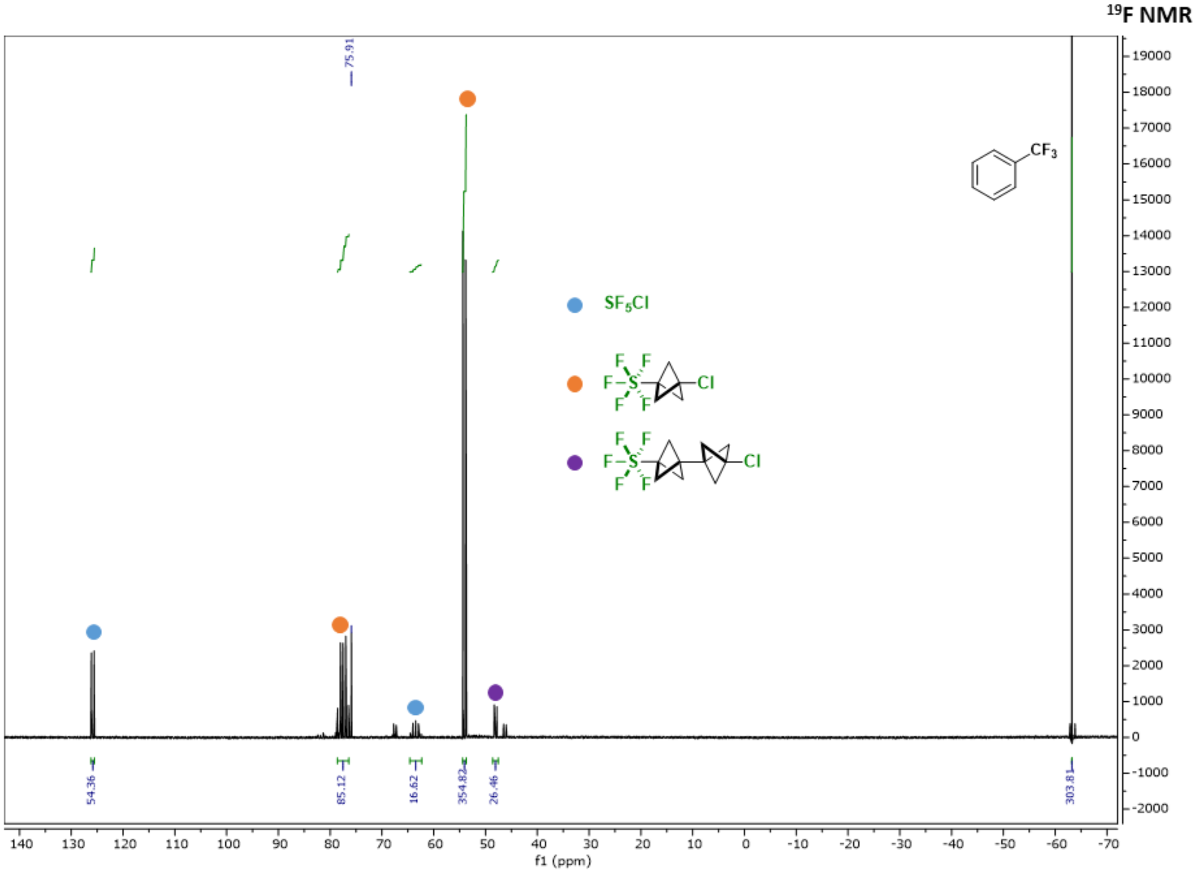

Figure S2: ^{19}F NMR spectrum of crude of synthesis of (3-chlorobicyclo[1.1.1]pentan-1-yl)pentafluoro-λ^6-sulfane (compound **2**). *Note: the ^{19}F shifts for (3'-chloro-[1,1'-bi(bicyclo[1.1.1]pentan)]-3-yl)pentafluoro-λ^6-sulfane (compound **3**) are also given.*

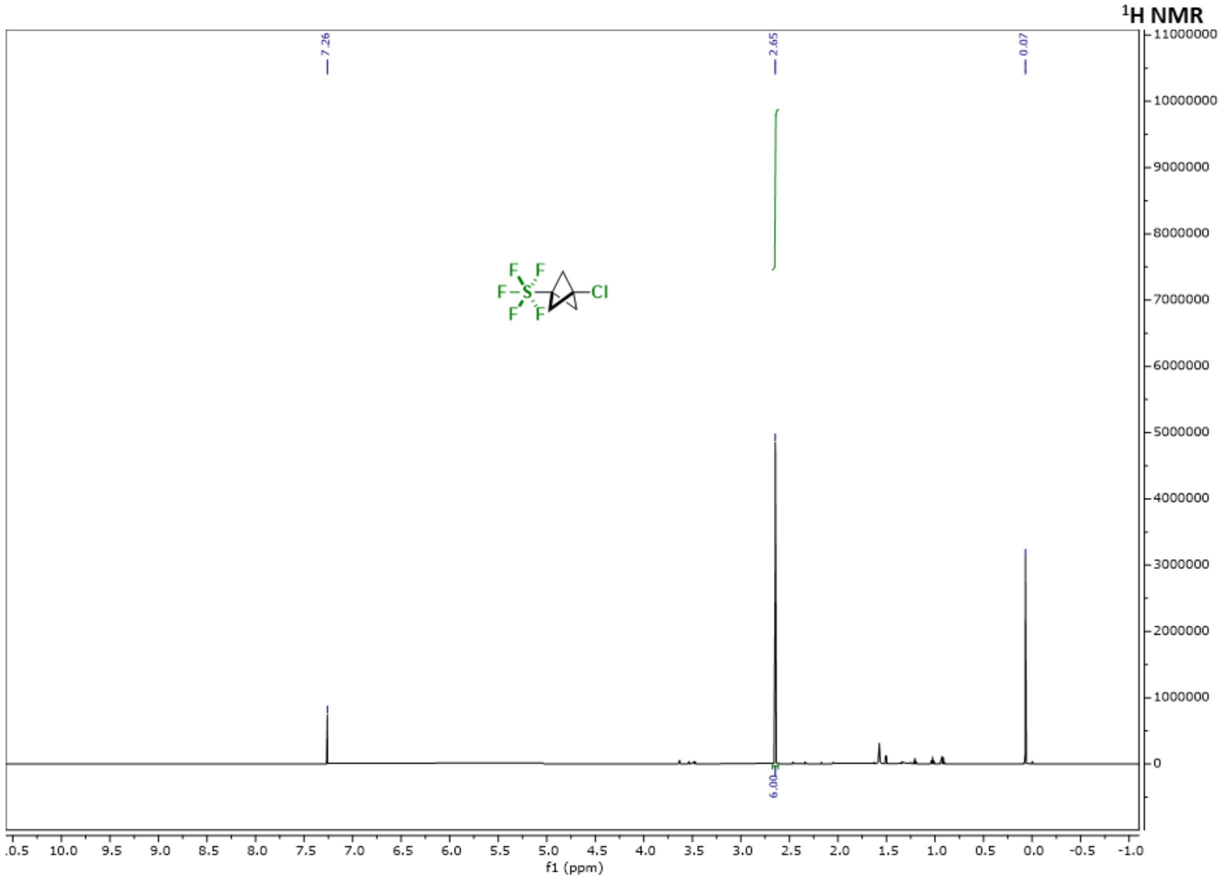

Figure S3: ^{1}H NMR spectrum of (3-chlorobicyclo[1.1.1]pentan-1-yl)pentafluoro-λ^6-sulfane (compound **2**). *Note: minor solvent impurities and byproducts are still present after distillation.*

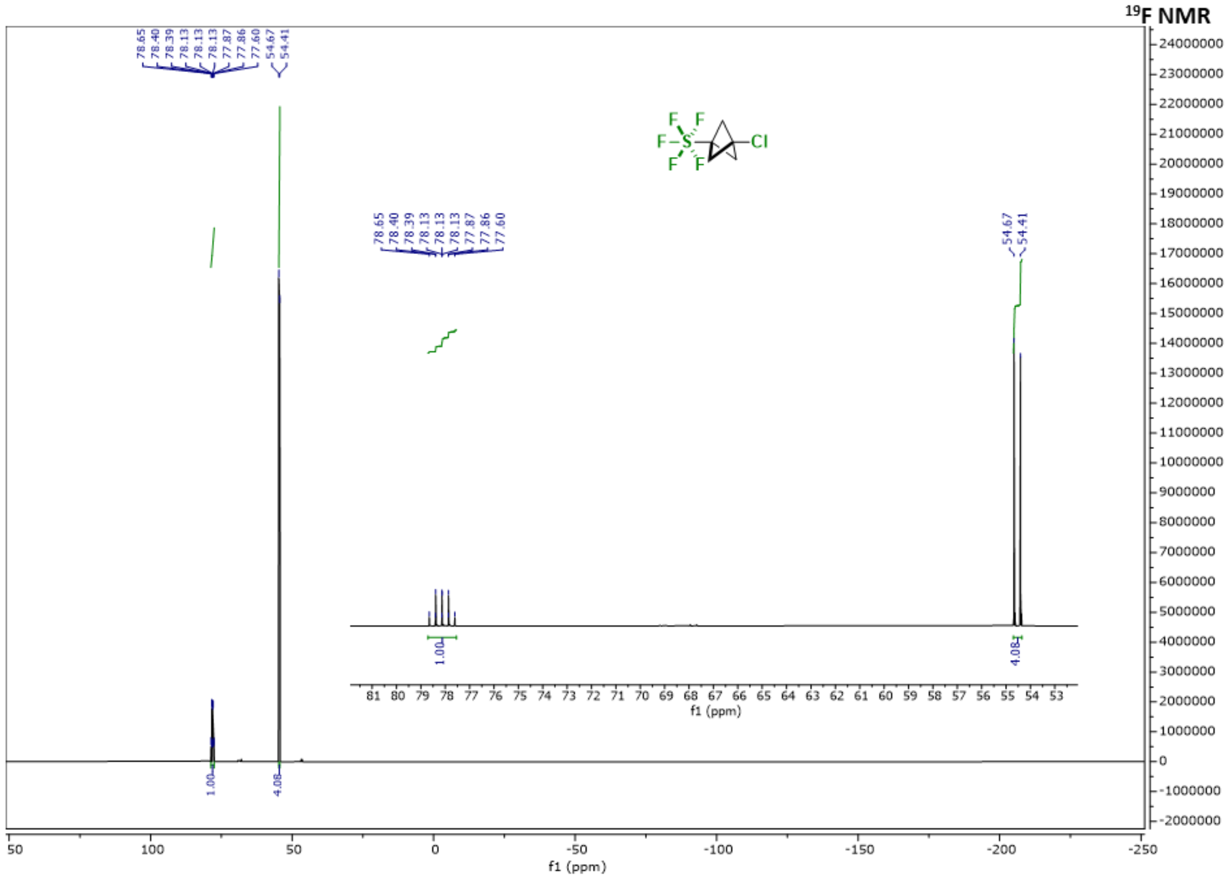

Figure S4: ^{19}F NMR spectrum of (3-chlorobicyclo[1.1.1]pentan-1-yl)pentafluoro-λ^{6}-sulfane (compound **2**).

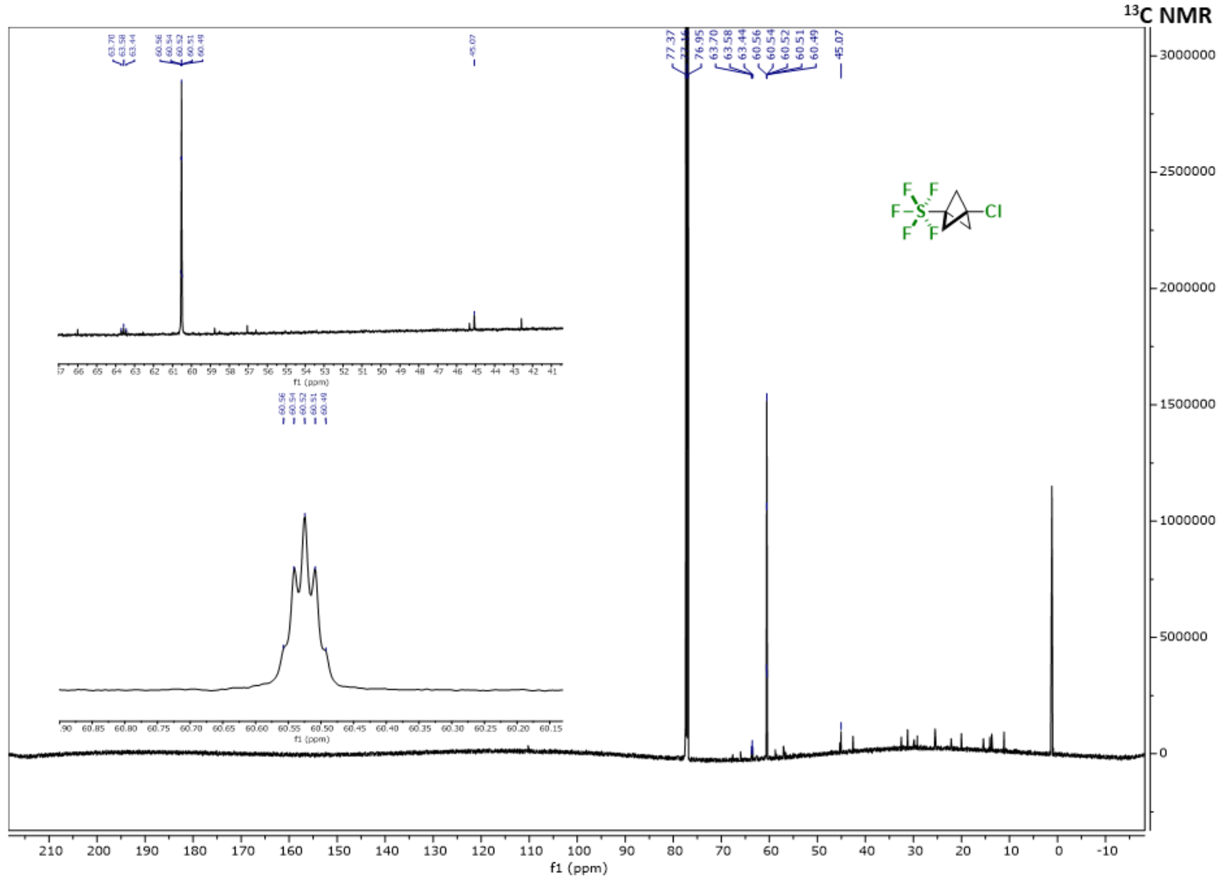

Figure S5: ^{13}C NMR spectrum of (3-chlorobicyclo[1.1.1]pentan-1-yl)pentafluoro-λ^{6}-sulfane (compound **2**). *Note: minor solvent impurities and byproducts are still present after distillation and appear more significant due to presumed poor relaxation times as well as multiplicity of the ^{13}C signals in compound* ***2****. See the following HMBC spectrum confirming assignments of chemical shifts.*

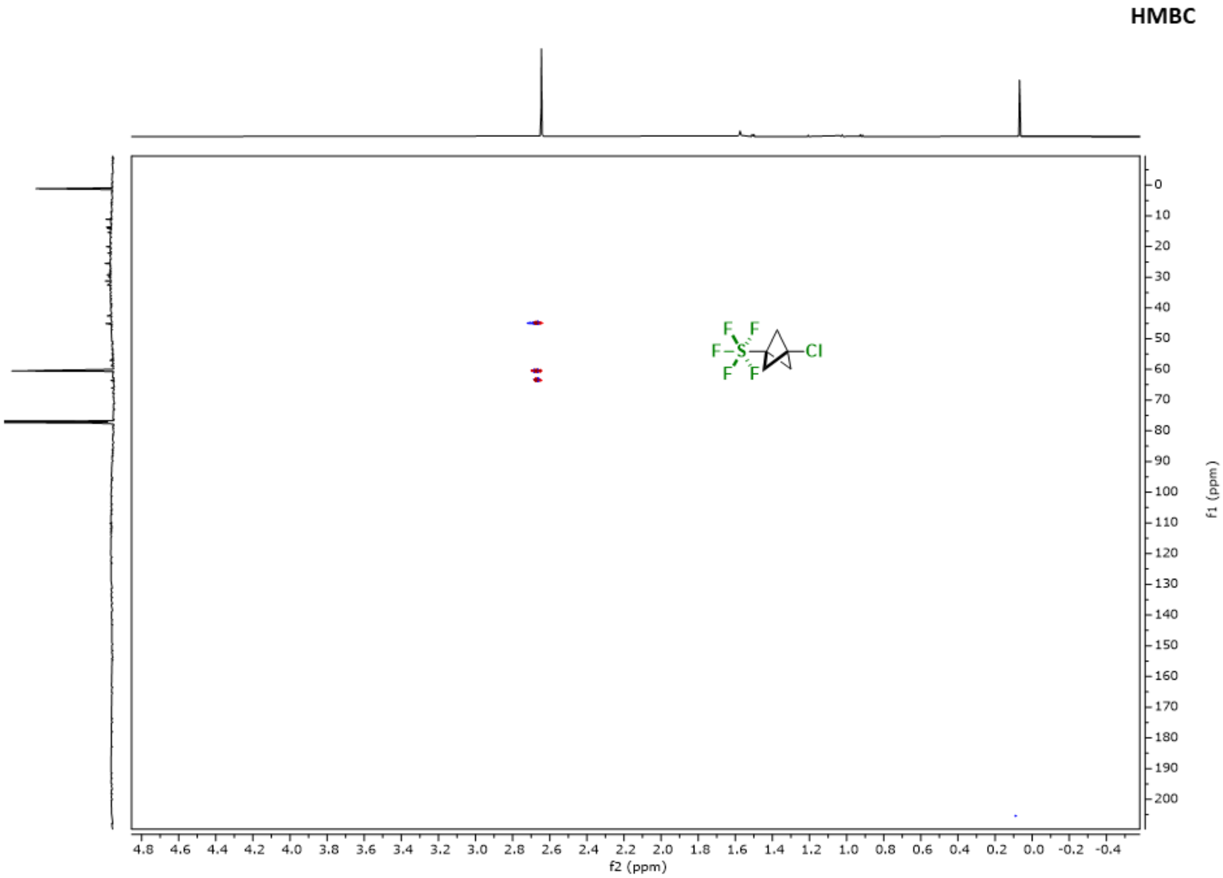

Figure S6: HMBC spectrum of (3-chlorobicyclo[1.1.1]pentan-1-yl)pentafluoro-λ^6-sulfane (compound **2**).

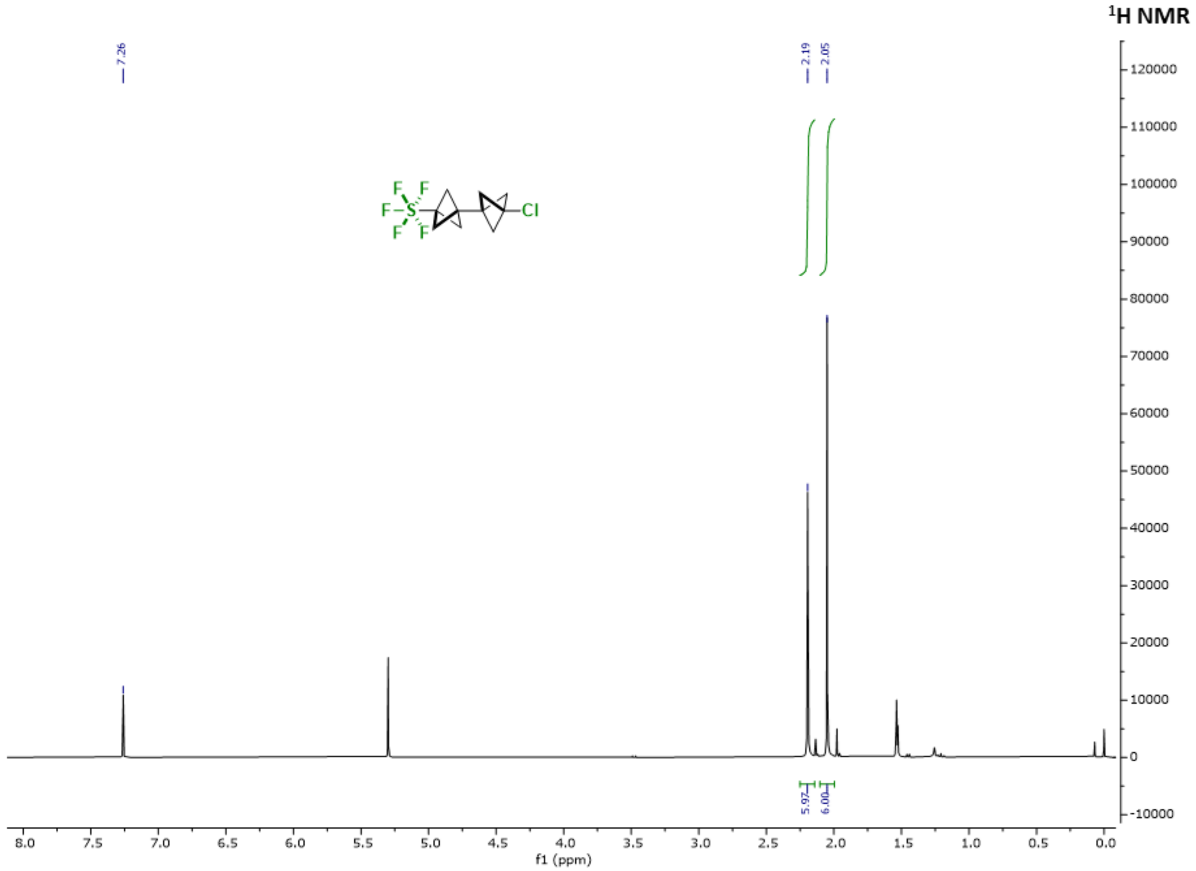

Figure S7: ^{1}H NMR spectrum of (3'-chloro-[1,1'-bi(bicyclo[1.1.1]pentan)]-3-yl)pentafluoro-λ^{6}-sulfane (compound **3**).

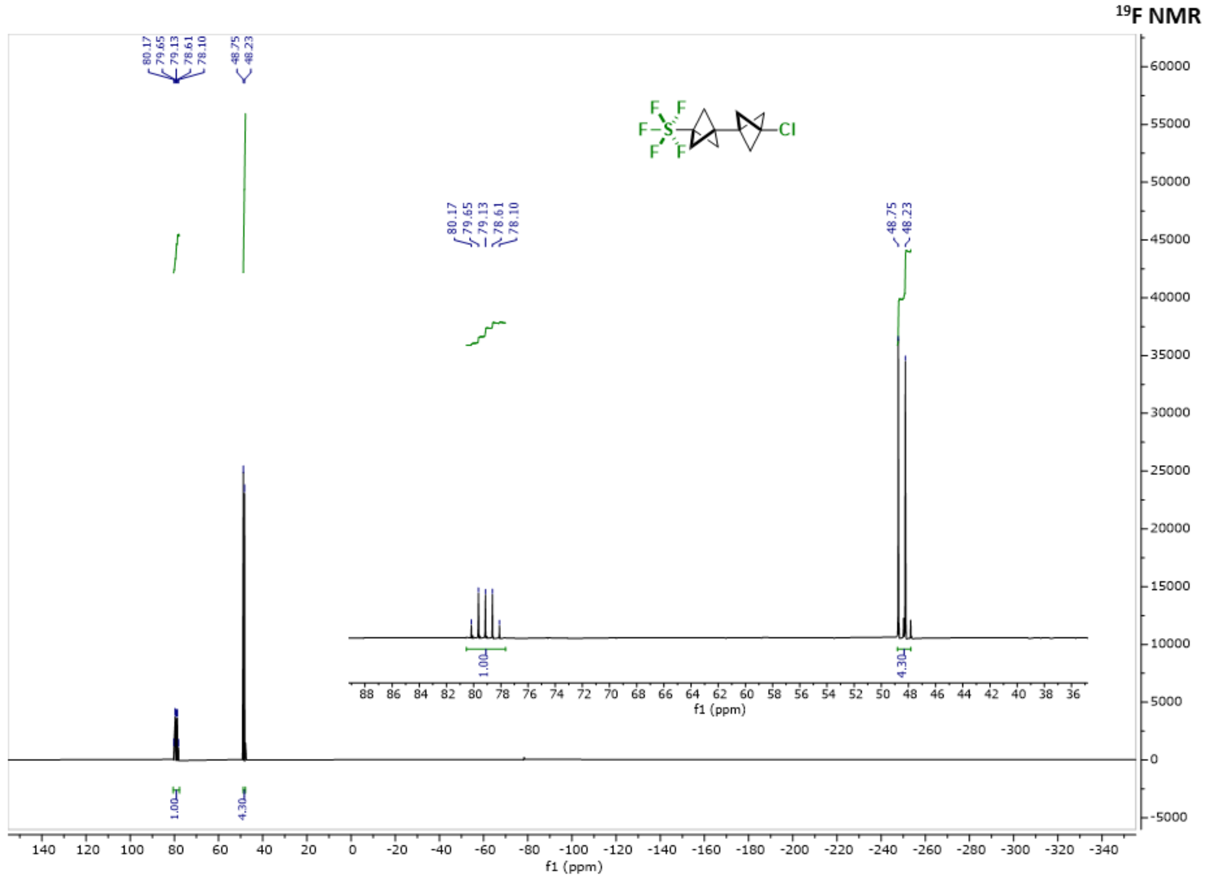

Figure S8: ^{19}F NMR spectrum of (3'-chloro-[1,1'-bi(bicyclo[1.1.1]pentan)]-3-yl)pentafluoro-λ^6-sulfane (compound **3**).

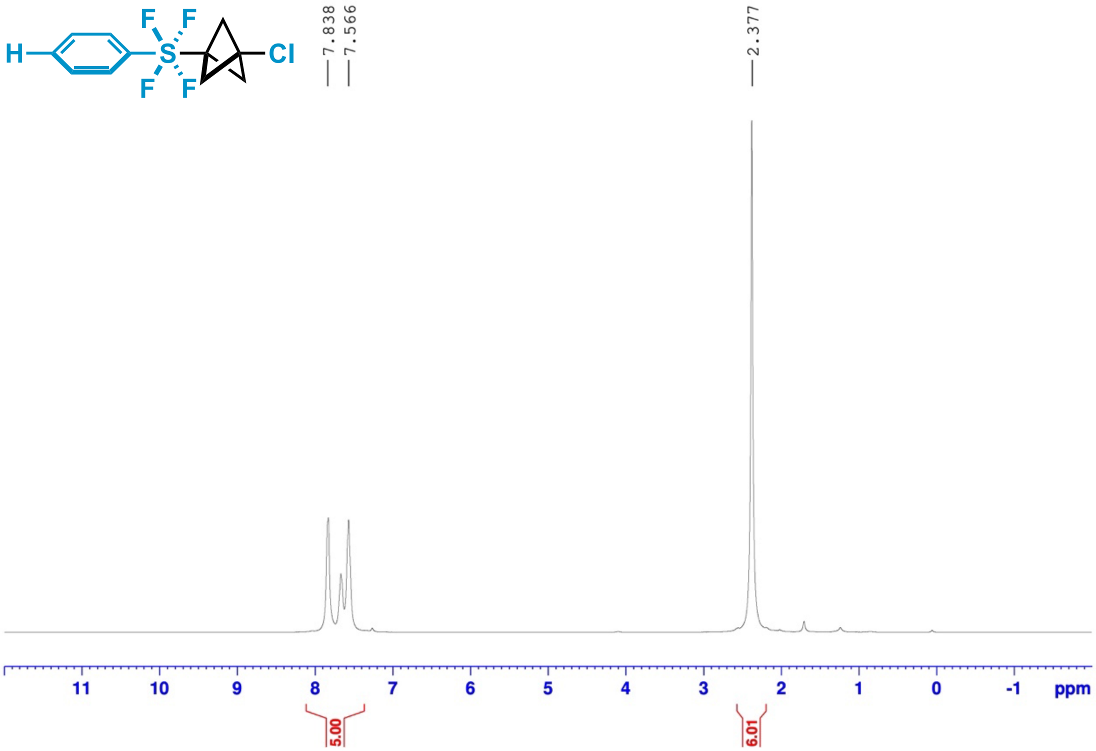

Figure S9: ^{1}H NMR spectrum of (3-chlorobicyclo[1.1.1]pentan-1-yl)tetrafluoro(phenyl)-λ^{6}-sulfane (compound **6**).

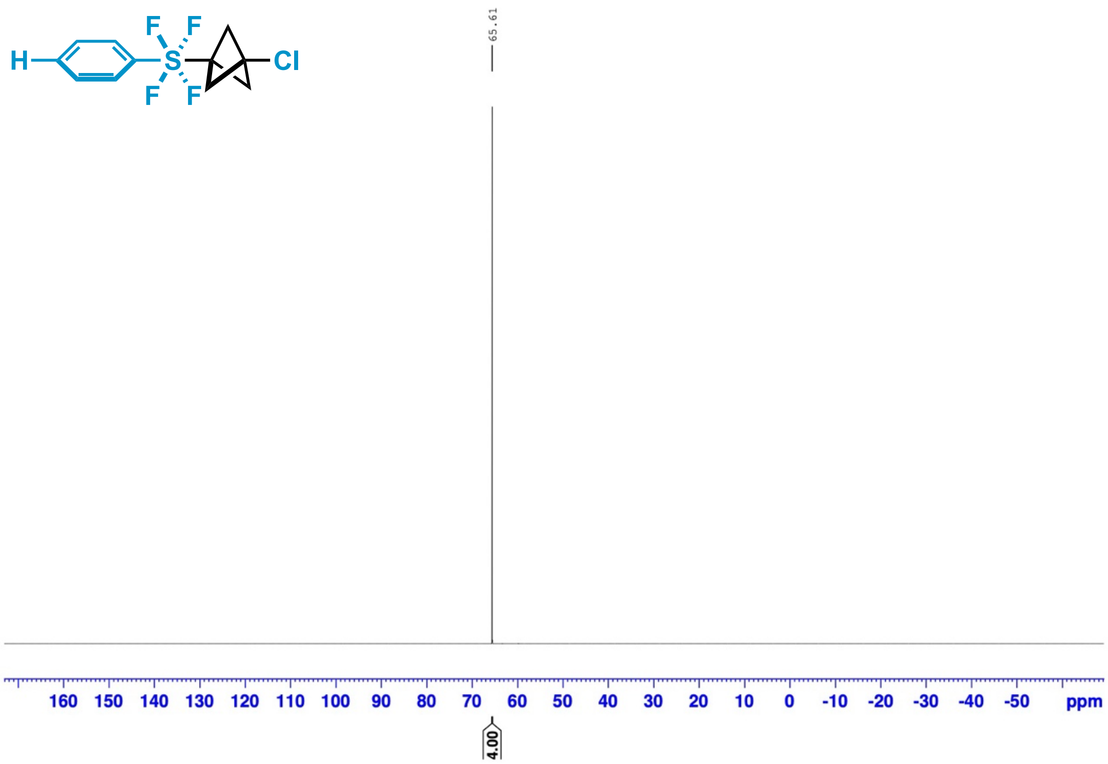

Figure S10: ^{19}F NMR spectrum of (3-chlorobicyclo[1.1.1]pentan-1-yl)tetrafluoro(phenyl)-λ^6-sulfane (compound **6**).

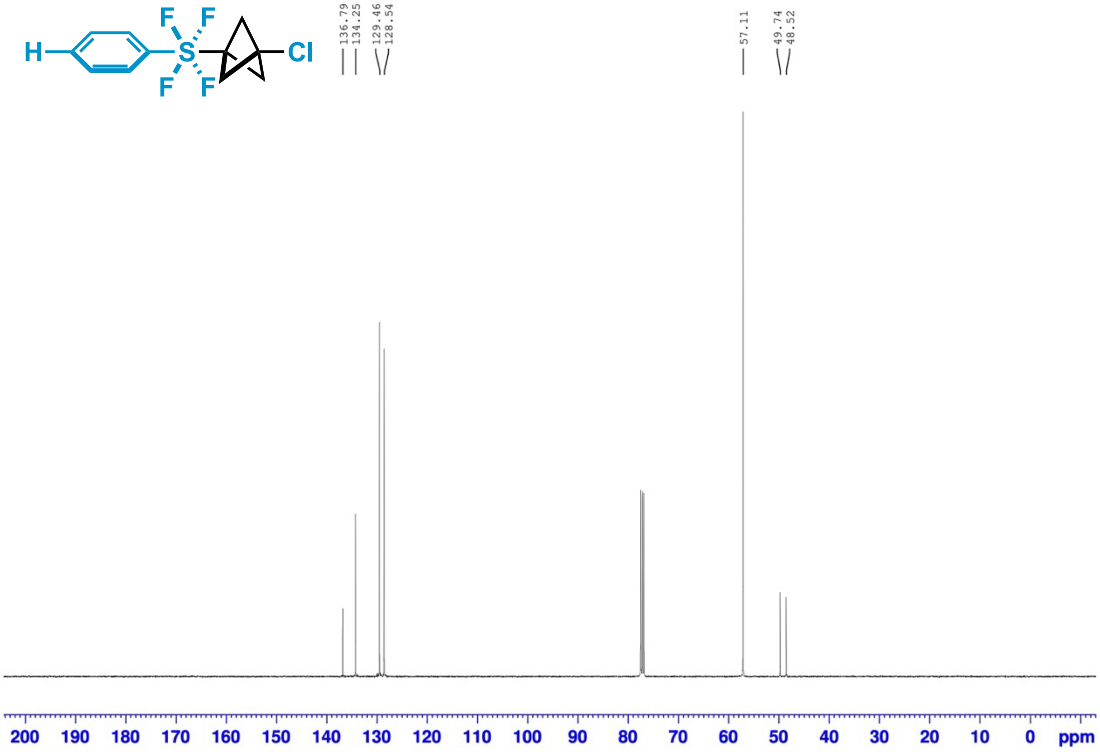

Figure S11: ^{13}C NMR spectrum of (3-chlorobicyclo[1.1.1]pentan-1-yl)tetrafluoro(phenyl)-λ^6-sulfane (compound **6**). *Note: the quintets of aryl and alkyl carbon atoms adjacent to the -SF_4- unit could not be resolved.*

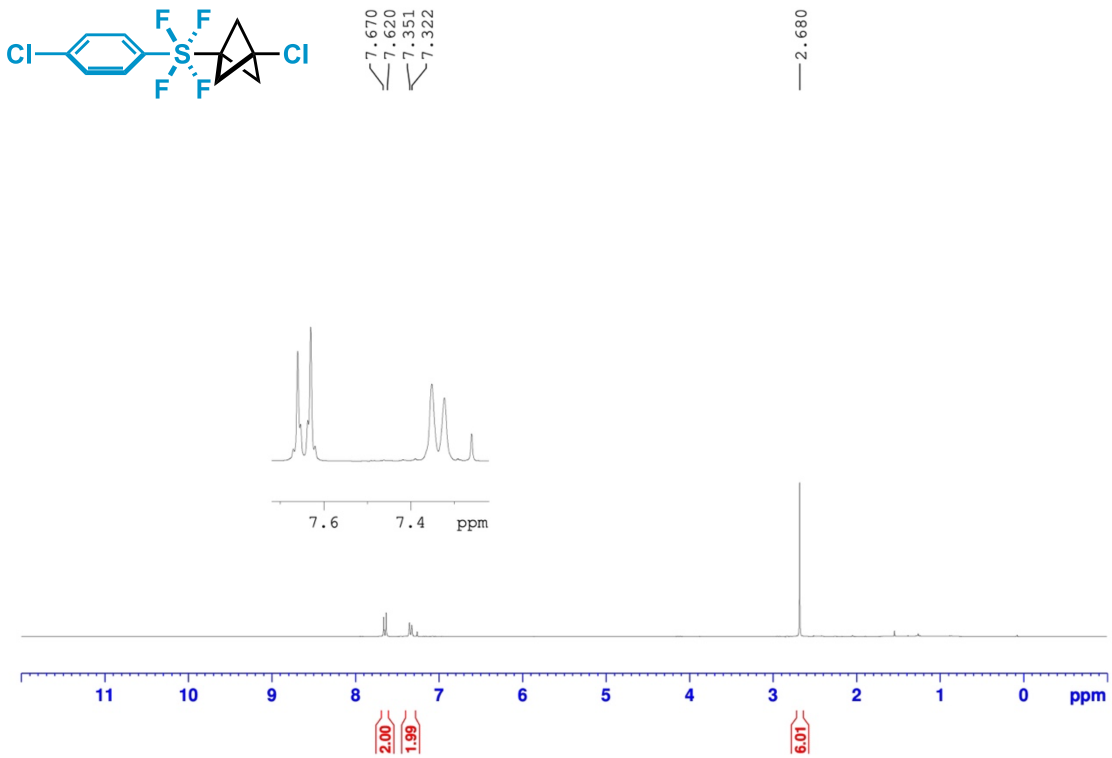

Figure S12: ^{1}H NMR spectrum of (3-chlorobicyclo[1.1.1]pentan-1-yl)(4-chlorophenyl)tetrafluoro-λ^{6}-sulfane (compound **7**).

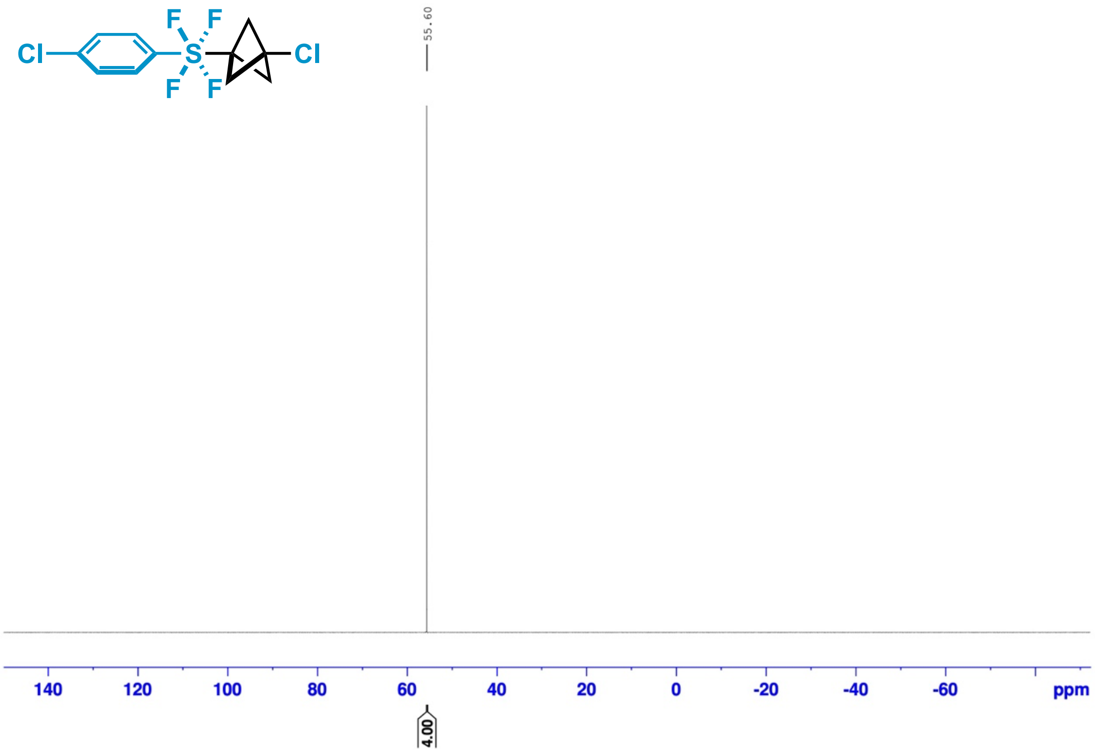

Figure S13: ^{19}F NMR spectrum of (3-chlorobicyclo[1.1.1]pentan-1-yl)(4-chlorophenyl)tetrafluoro-λ^{6}-sulfane (compound **7**).

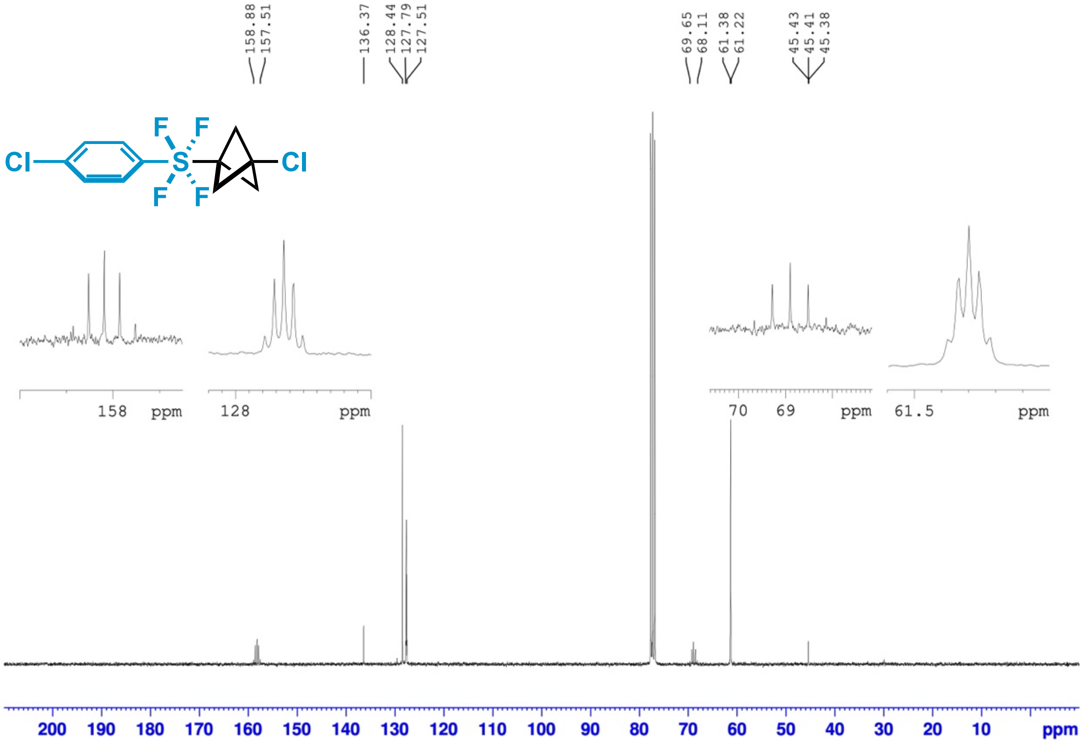

Figure S14: ^{13}C NMR spectrum of (3-chlorobicyclo[1.1.1]pentan-1-yl)(4-chlorophenyl)tetrafluoro-λ^{6}-sulfane (compound **7**).

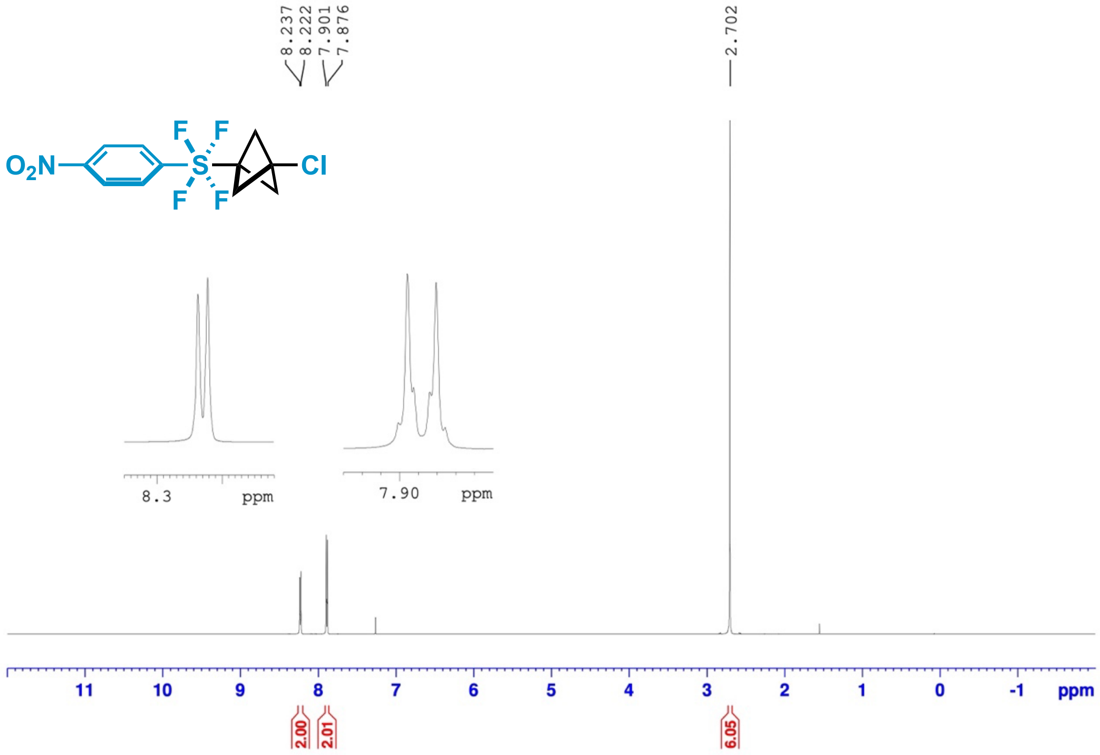

Figure S15: ^{1}H NMR spectrum of (3-chlorobicyclo[1.1.1]pentan-1-yl)tetrafluoro(4-nitrophenyl)-λ^6-sulfane (compound **8**).

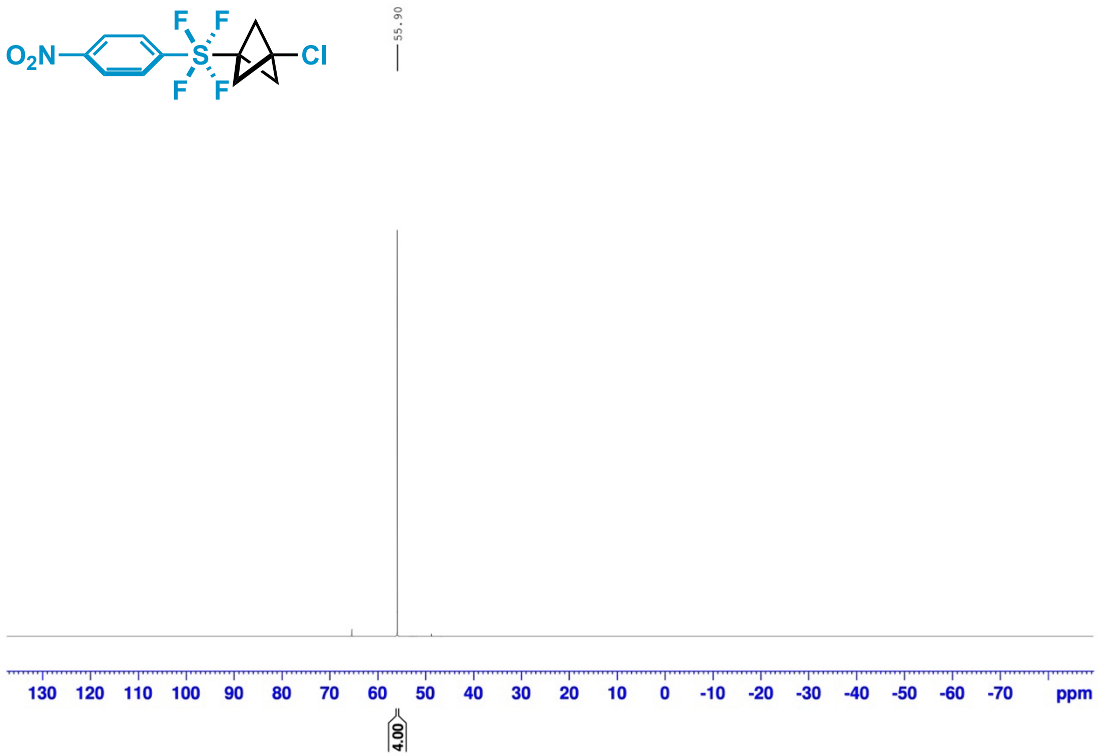

Figure S16: ^{19}F NMR spectrum of (3-chlorobicyclo[1.1.1]pentan-1-yl)tetrafluoro(4-nitrophenyl)-λ^6-sulfane (compound **8**).

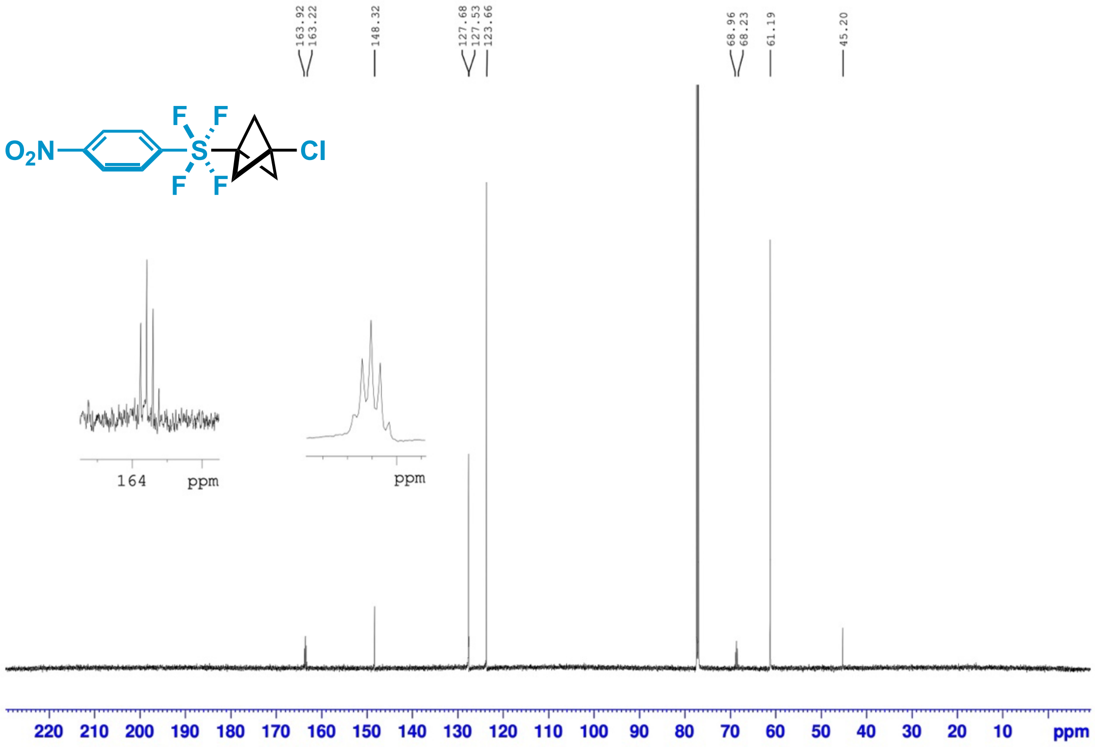

Figure S17: ^{13}C NMR spectrum of (3-chlorobicyclo[1.1.1]pentan-1-yl)tetrafluoro(4-nitrophenyl)-λ^6-sulfane (compound **8**).

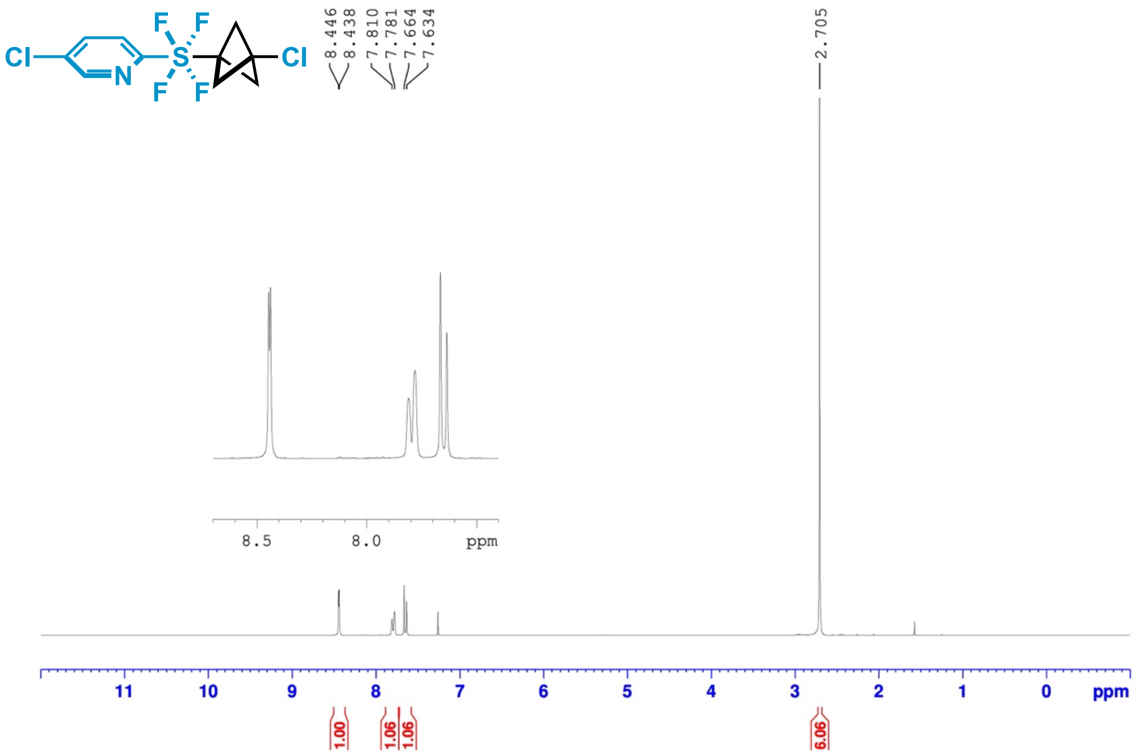

Figure S18: ^{1}H NMR spectrum of 5-chloro-2-((3-chlorobicyclo[1.1.1]pentan-1-yl)tetrafluoro-λ^{6}-sulfaneyl)pyridine (compound **9**).

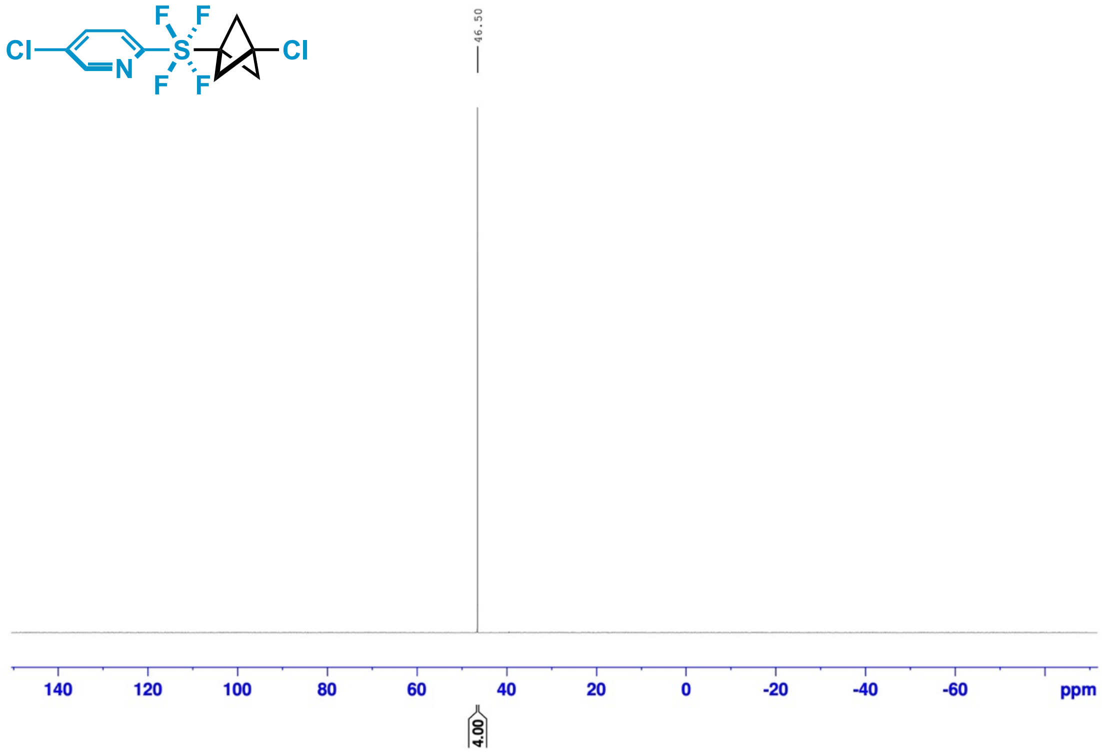

Figure S19: ^{19}F NMR spectrum of 5-chloro-2-((3-chlorobicyclo[1.1.1]pentan-1-yl)tetrafluoro-λ^{6}-sulfaneyl)pyridine (compound **9**).

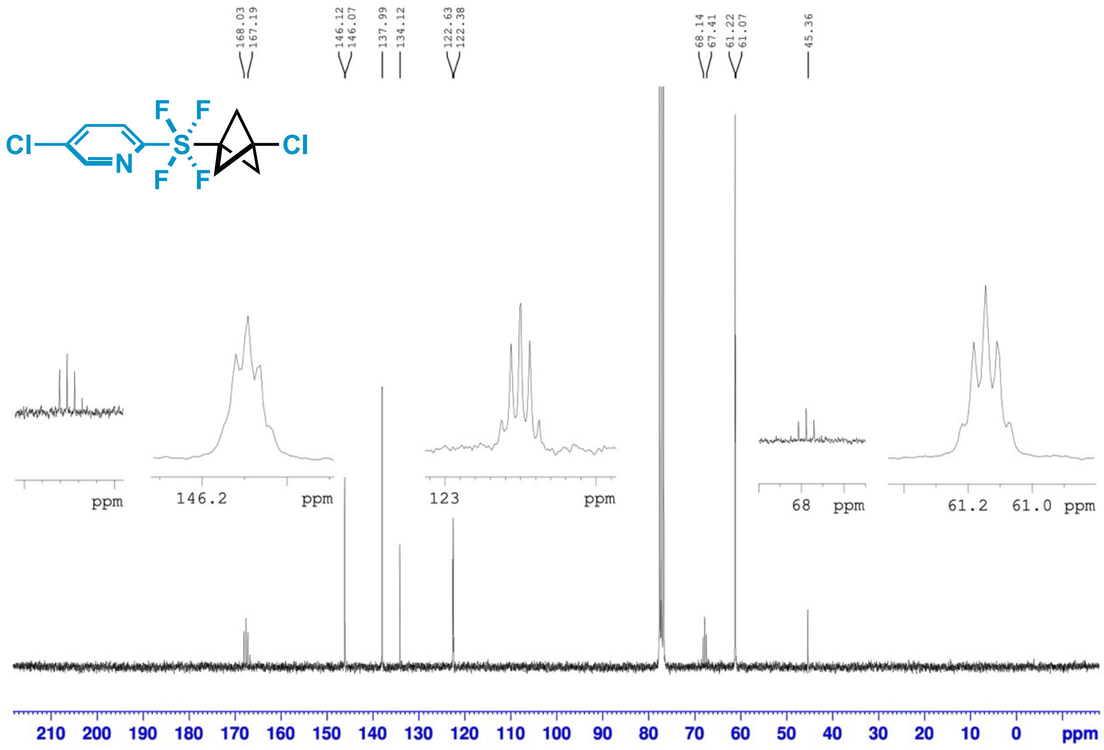

Figure S20: ^{13}C NMR spectrum of 5-chloro-2-((3-chlorobicyclo[1.1.1]pentan-1-yl)tetrafluoro-λ^6-sulfaneyl)pyridine (compound **9**).

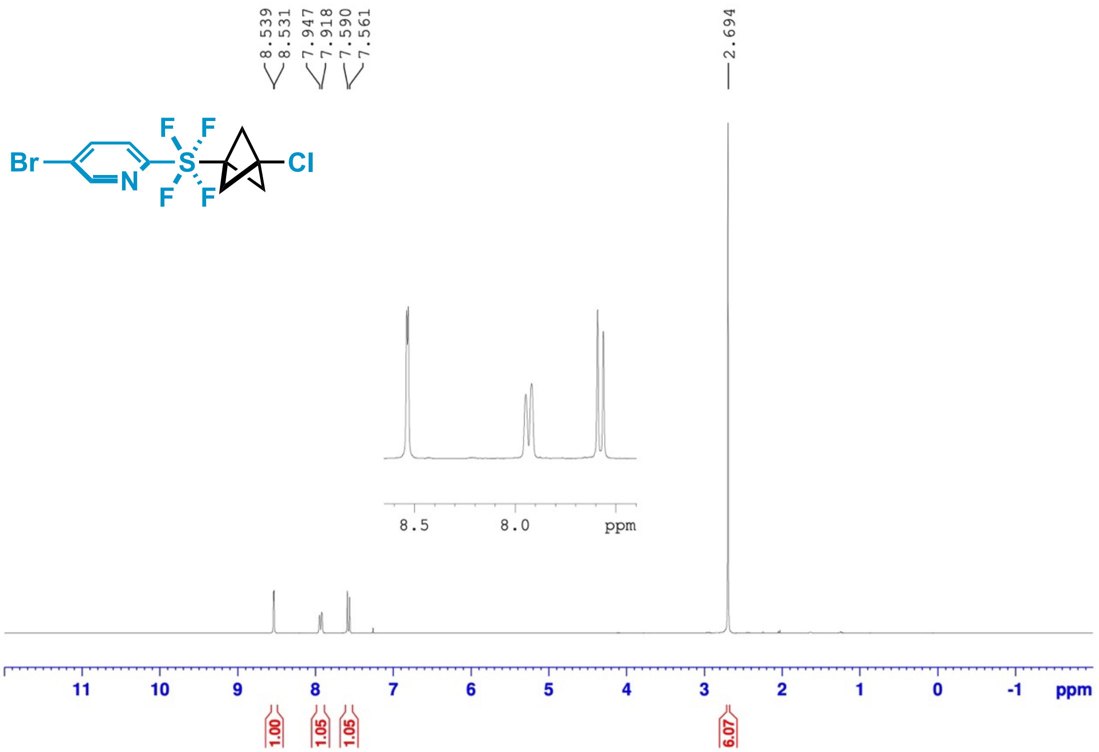

Figure S21: ^{1}H NMR spectrum of 5-bromo-2-((3-chlorobicyclo[1.1.1]pentan-1-yl)tetrafluoro-λ^6-sulfaneyl)pyridine (compound **10**).

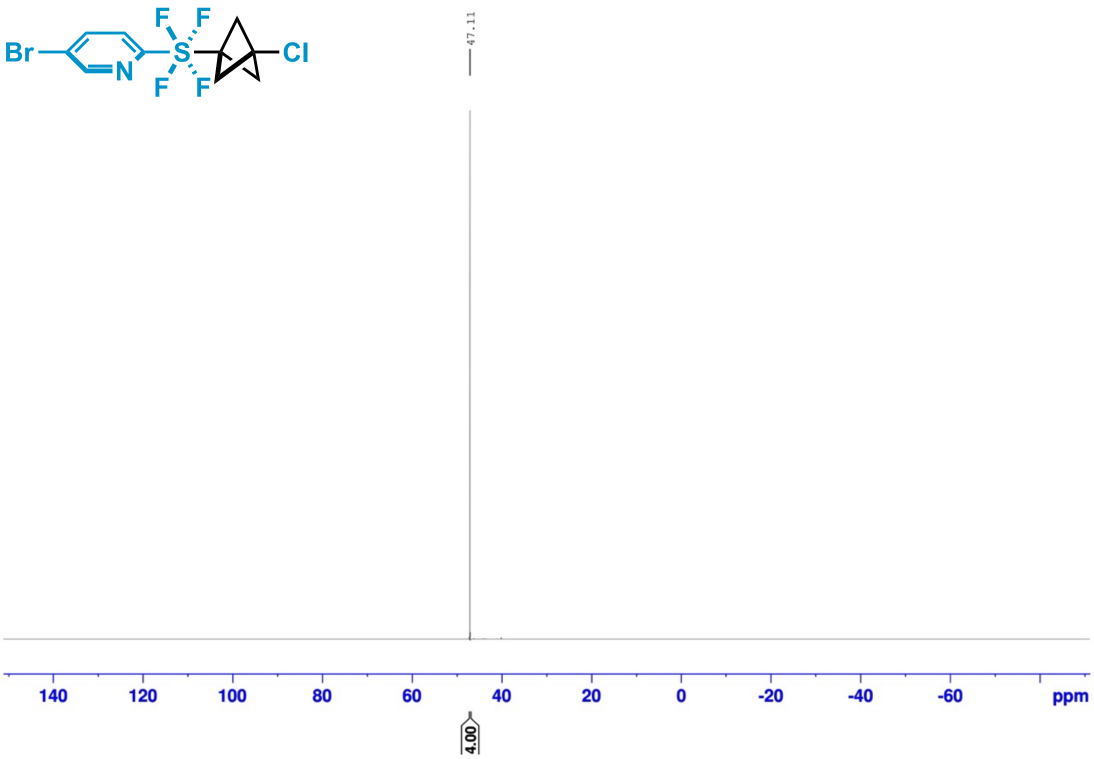

Figure S22: ^{19}F NMR spectrum of 5-bromo-2-((3-chlorobicyclo[1.1.1]pentan-1-yl)tetrafluoro-λ^{6}-sulfaneyl)pyridine (compound **10**).

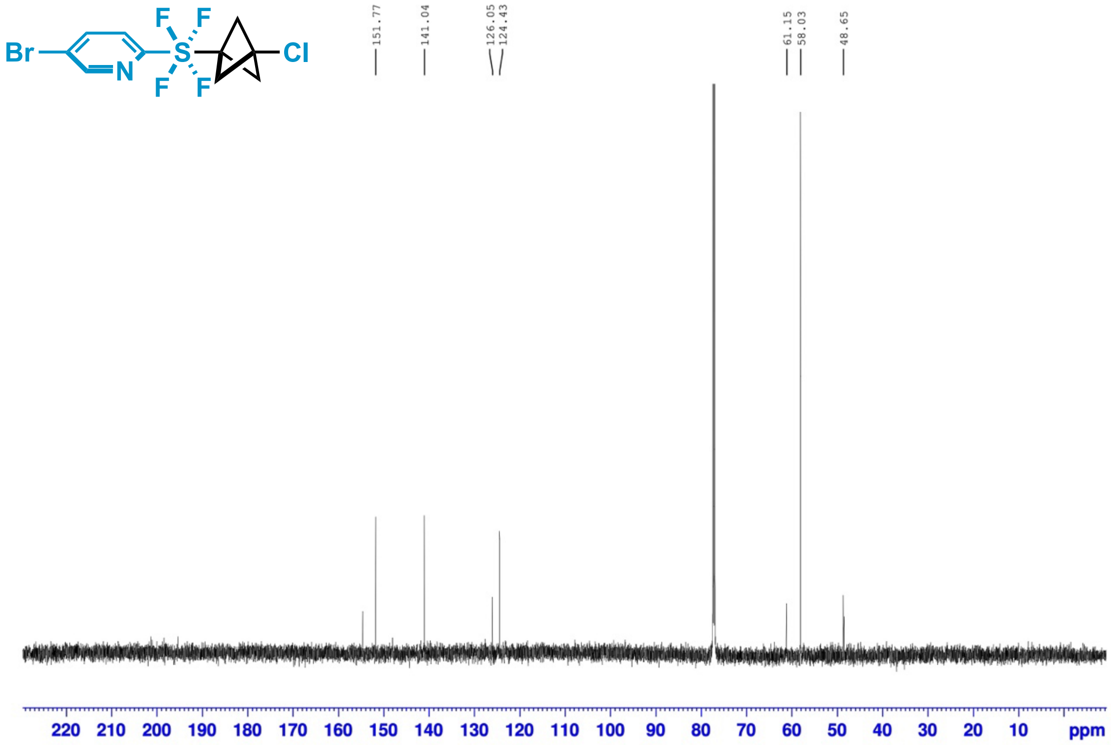

Figure S23: ^{13}C NMR spectrum of 5-bromo-2-((3-chlorobicyclo[1.1.1]pentan-1-yl)tetrafluoro-λ^{6}-sulfaneyl)pyridine (compound **10**). *Note: the quintets of aryl and alkyl carbon atoms adjacent to the -SF_4- unit could not be resolved.*

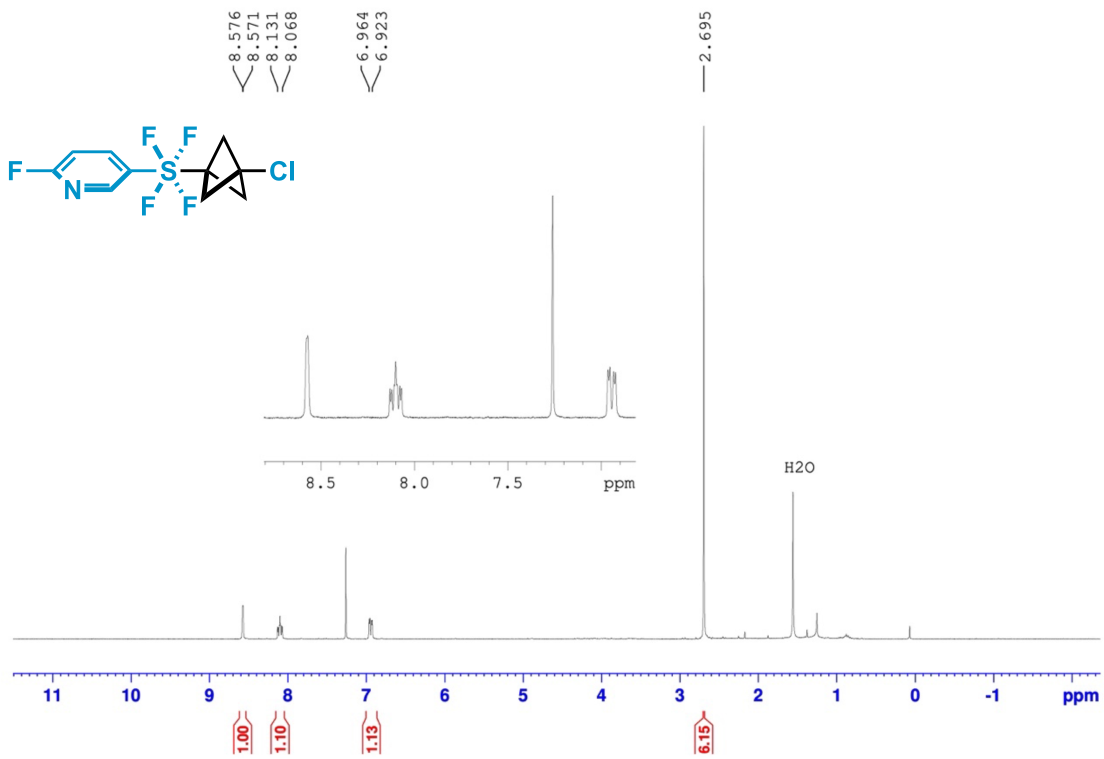

Figure S24: ^{1}H NMR spectrum of 5-((3-chlorobicyclo[1.1.1]pentan-1-yl)tetrafluoro-λ^6-sulfaneyl)-2-fluoropyridine (compound **11**).

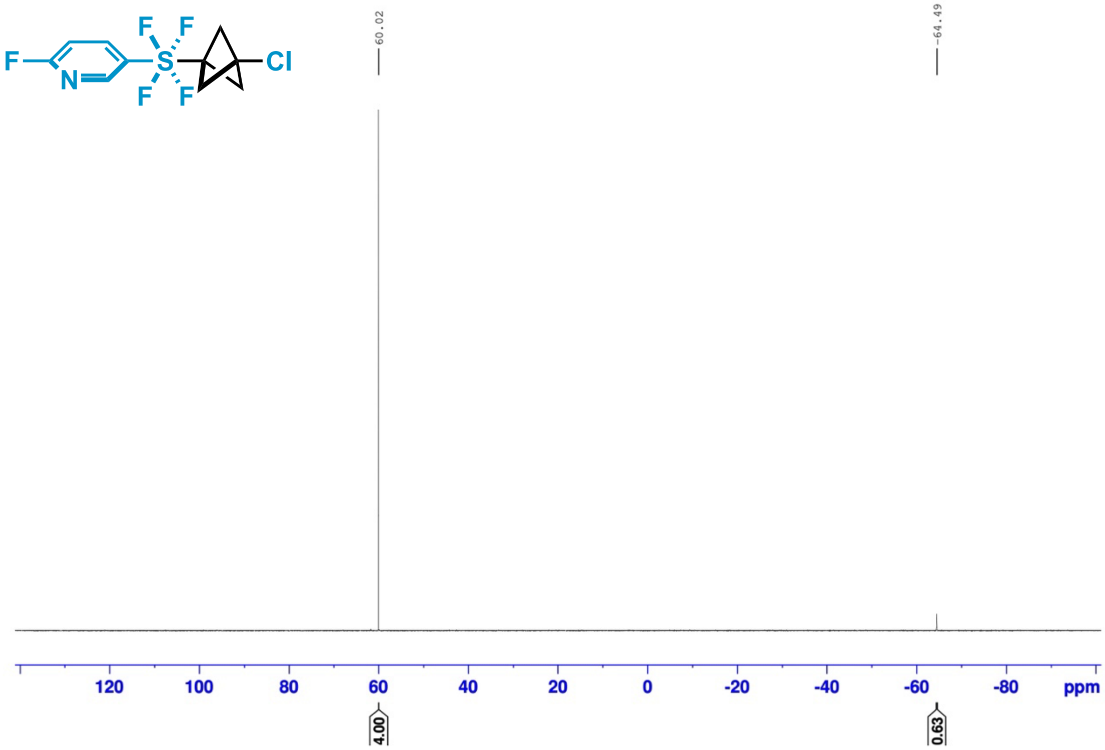

Figure S25: ^{19}F NMR spectrum of 5-((3-chlorobicyclo[1.1.1]pentan-1-yl)tetrafluoro-λ^6-sulfaneyl)-2-fluoropyridine (compound **11**).

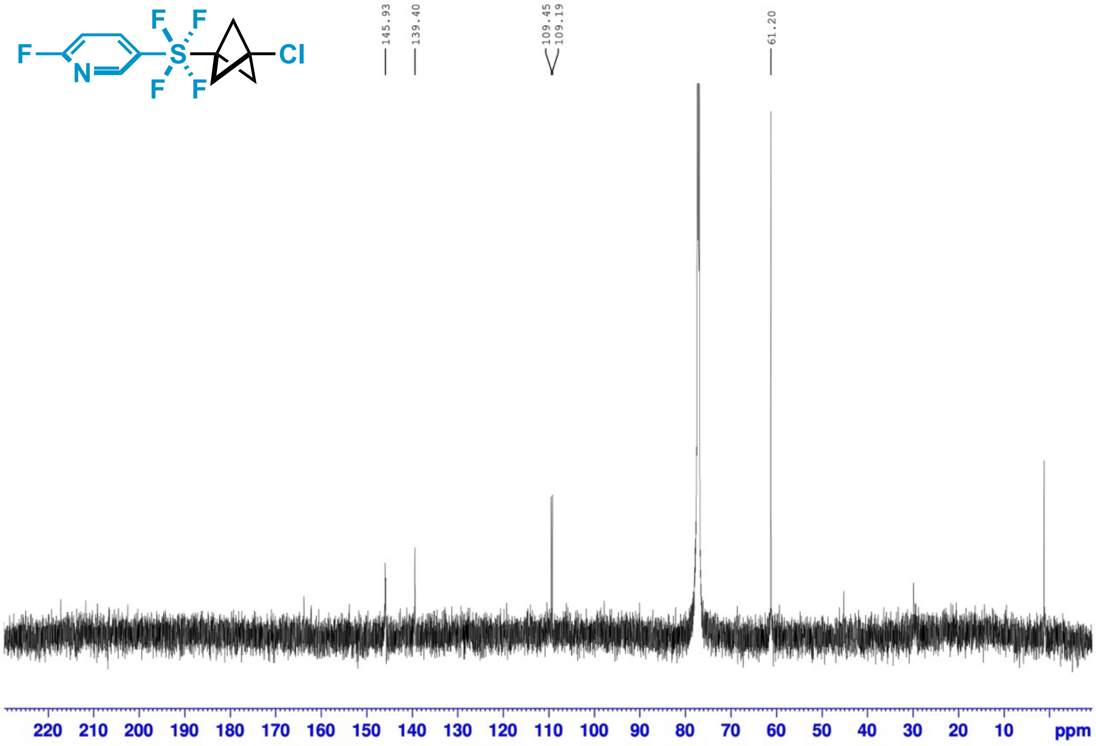

Figure S26: ^{13}C NMR spectrum of 5-((3-chlorobicyclo[1.1.1]pentan-1-yl)tetrafluoro-λ^6-sulfaneyl)-2-fluoropyridine (compound **11**).

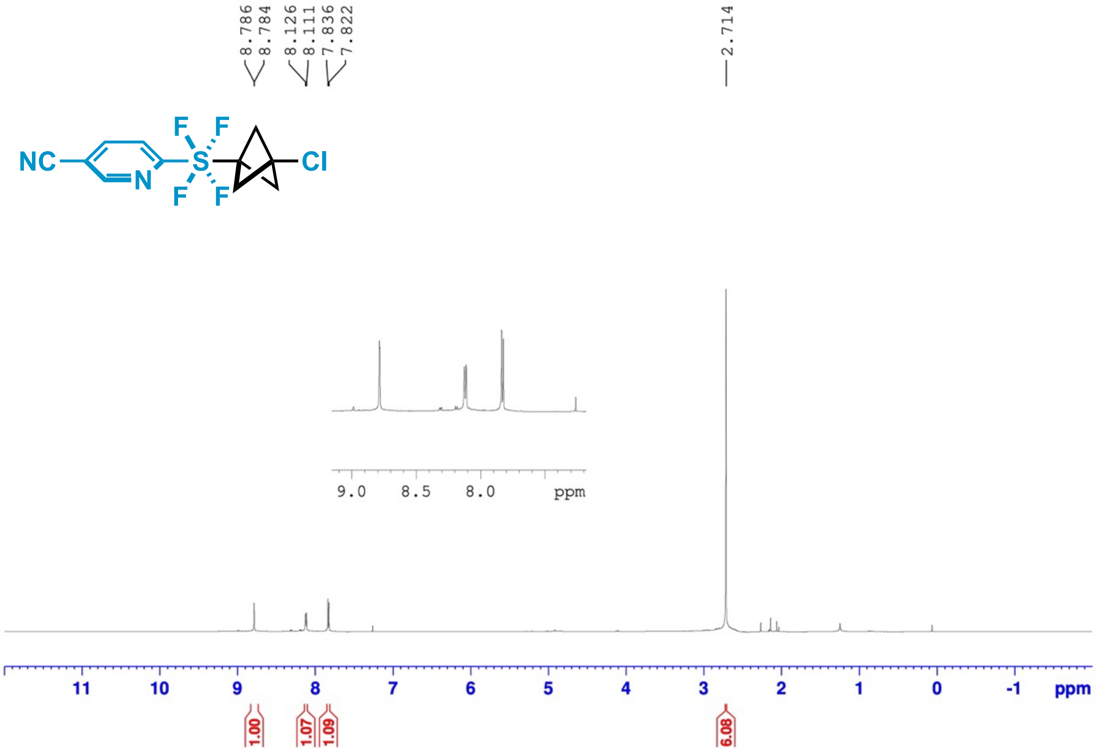

Figure S27: ^{1}H NMR spectrum of 6-((3-chlorobicyclo[1.1.1]pentan-1-yl)tetrafluoro-λ^6-sulfaneyl)nicotinonitrile (compound **12**). *Note: Compound **12** was prone to degradation with extended exposure to air and glass.*

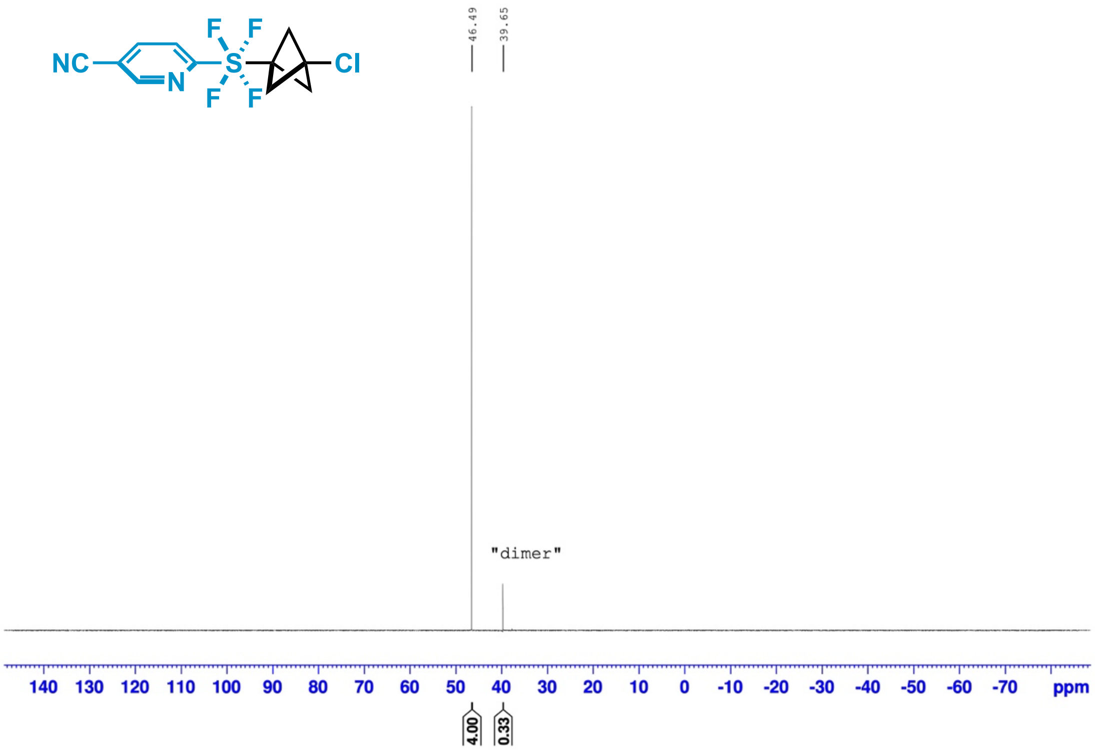

Figure S28: ^{19}F NMR spectrum of 6-((3-chlorobicyclo[1.1.1]pentan-1-yl)tetrafluoro-λ^6-sulfaneyl)nicotinonitrile (compound **12**).

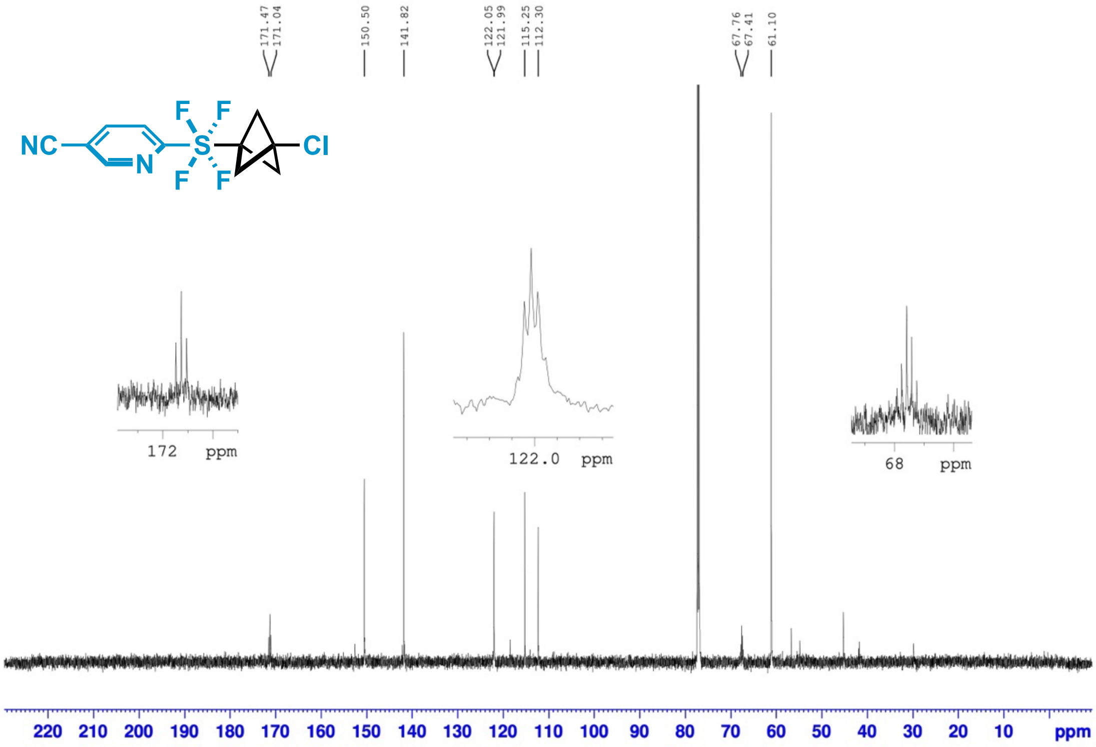

Figure S29: ^{13}C NMR spectrum of 6-((3-chlorobicyclo[1.1.1]pentan-1-yl)tetrafluoro-λ^{6}-sulfaneyl)nicotinonitrile (compound **12**).

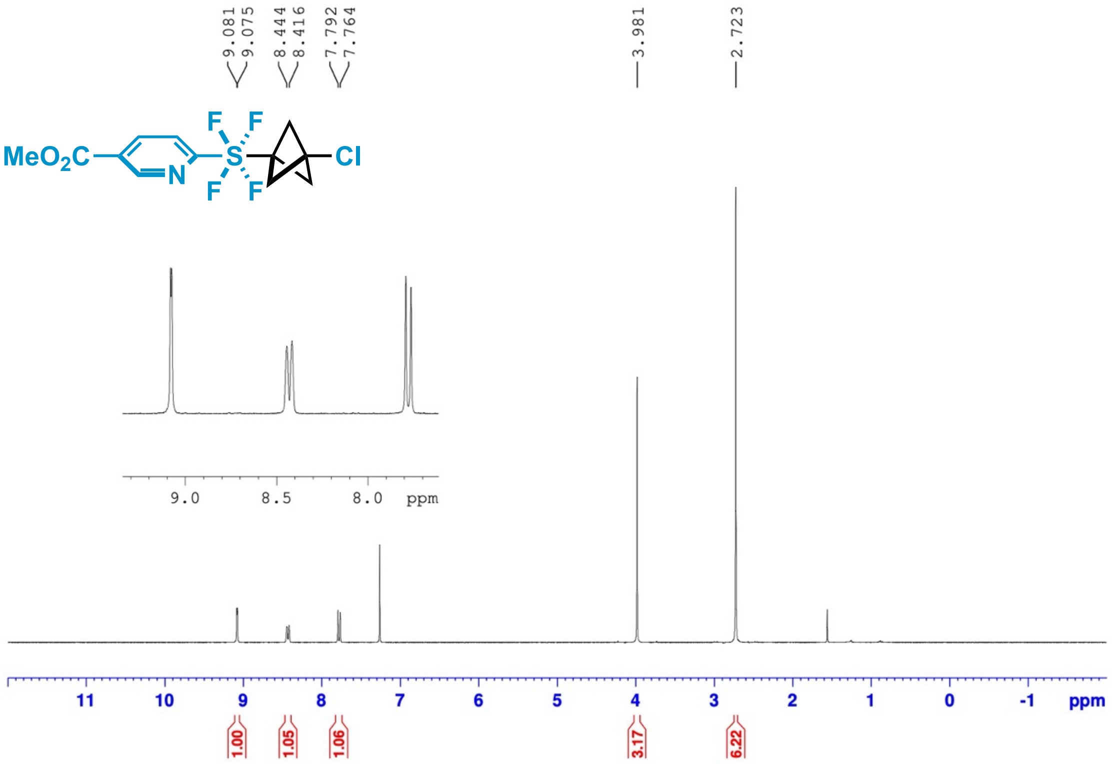

Figure S30: ^{1}H NMR spectrum of methyl 6-((3-chlorobicyclo[1.1.1]pentan-1-yl)tetrafluoro-λ^6-sulfaneyl)nicotinate (compound **13**).

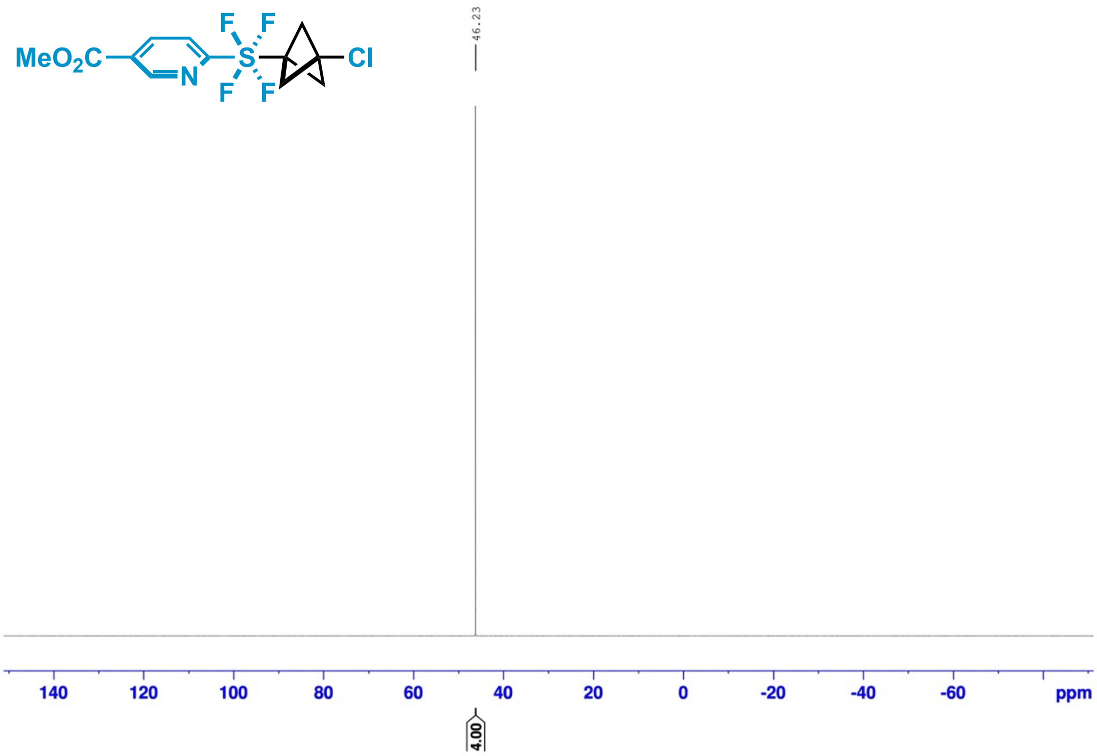

Figure S31: ^{19}F NMR spectrum of methyl 6-((3-chlorobicyclo[1.1.1]pentan-1-yl)tetrafluoro-λ^6-sulfaneyl)nicotinate (compound **13**).

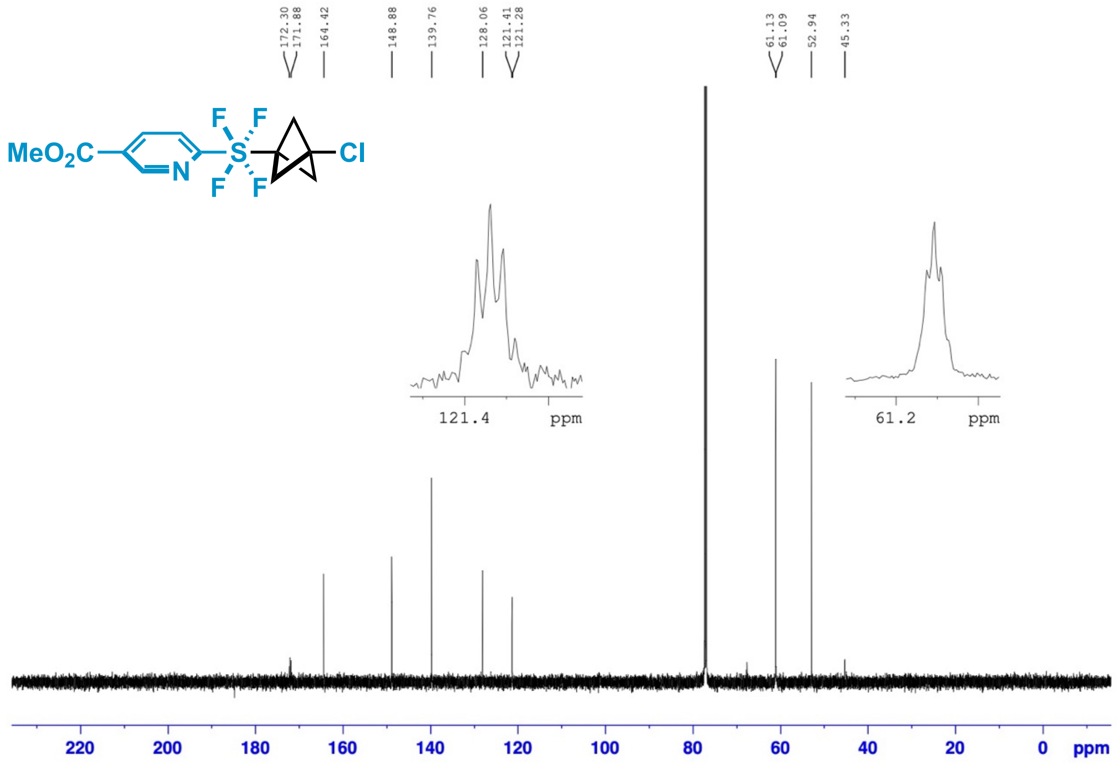

Figure S32: ^{13}C NMR spectrum of methyl 6-((3-chlorobicyclo[1.1.1]pentan-1-yl)tetrafluoro-λ^6-sulfaneyl)nicotinate (compound **13**).

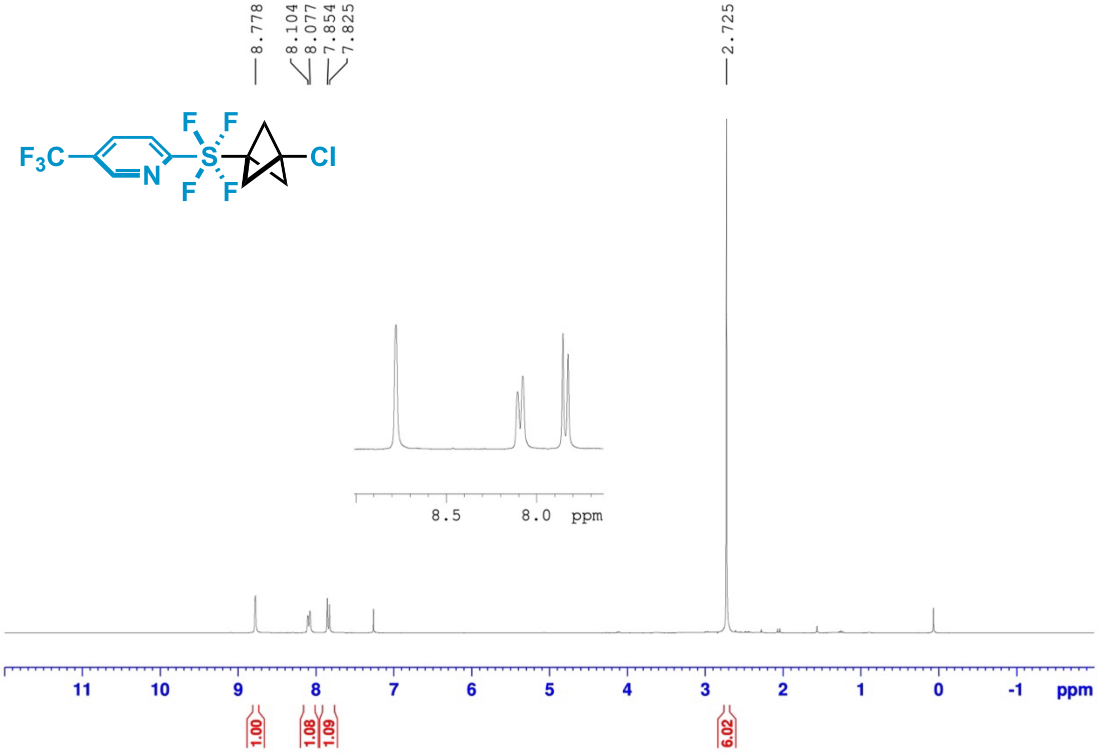

Figure S33: ^{1}H NMR spectrum of 2-((3-chlorobicyclo[1.1.1]pentan-1-yl)tetrafluoro-λ^{6}-sulfaneyl)-5-(trifluoromethyl)pyridine (compound **14**).

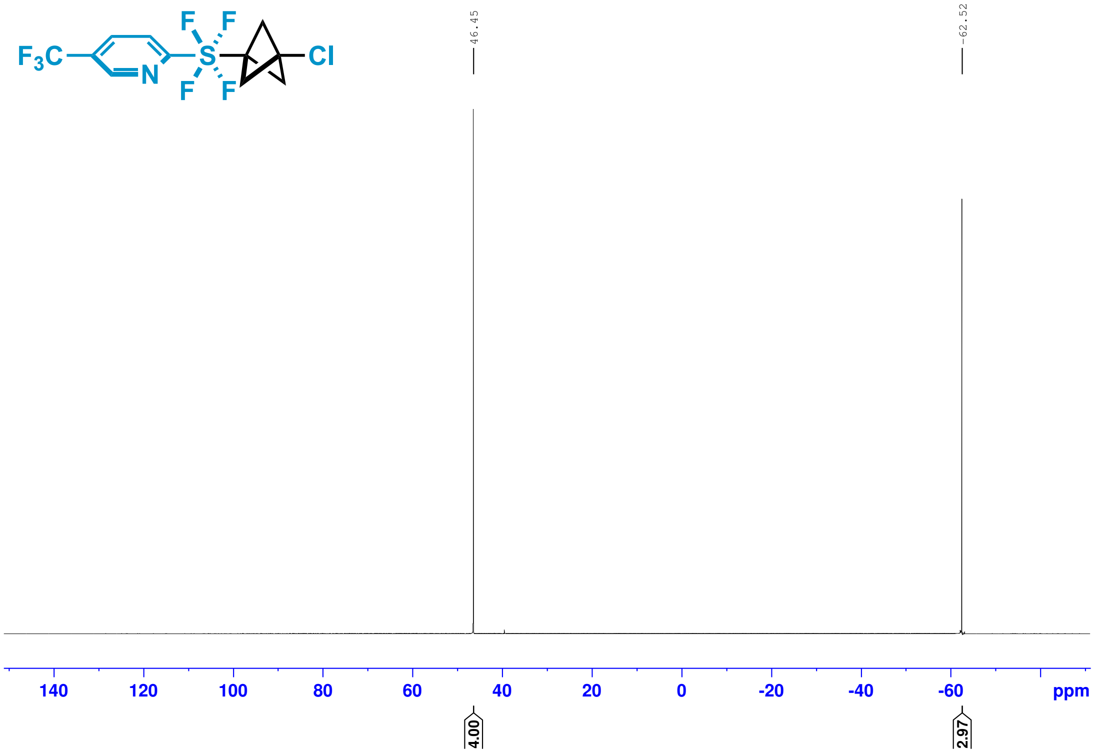

Figure S34: ^{19}F NMR spectrum of 2-((3-chlorobicyclo[1.1.1]pentan-1-yl)tetrafluoro-λ^6-sulfaneyl)-5-(trifluoromethyl)pyridine (compound **14**).

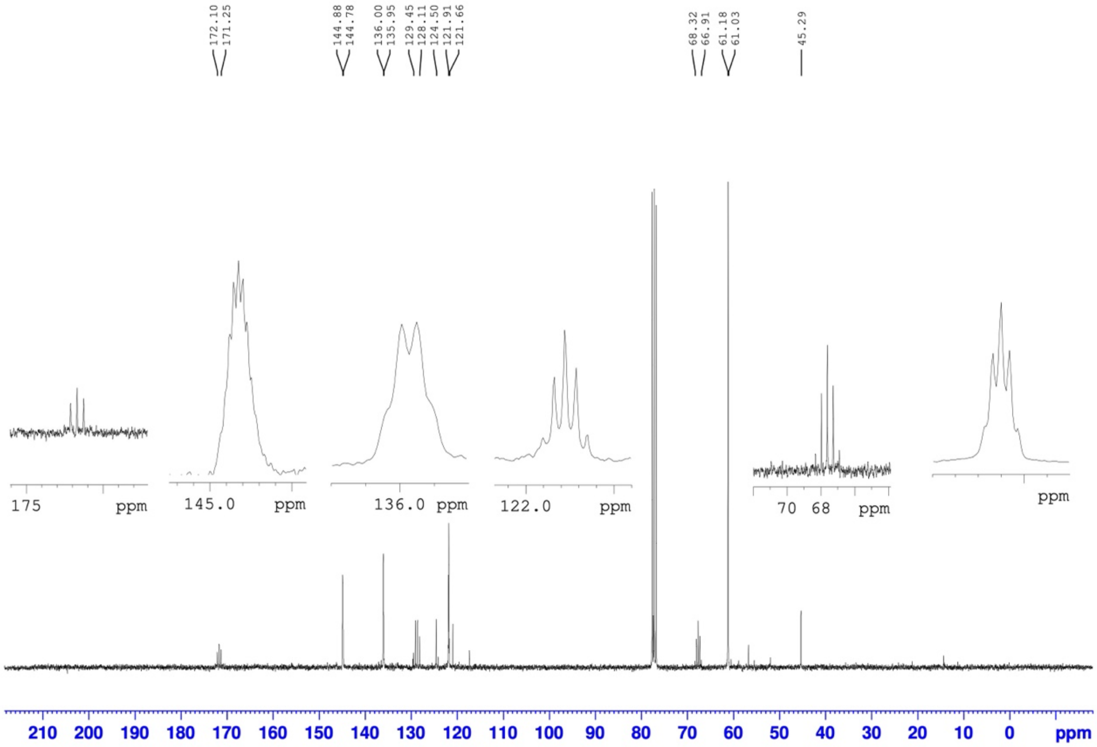

Figure S35: ^{13}C NMR spectrum of 2-((3-chlorobicyclo[1.1.1]pentan-1-yl)tetrafluoro-λ^6-sulfaneyl)-5-(trifluoromethyl)pyridine (compound **14**).

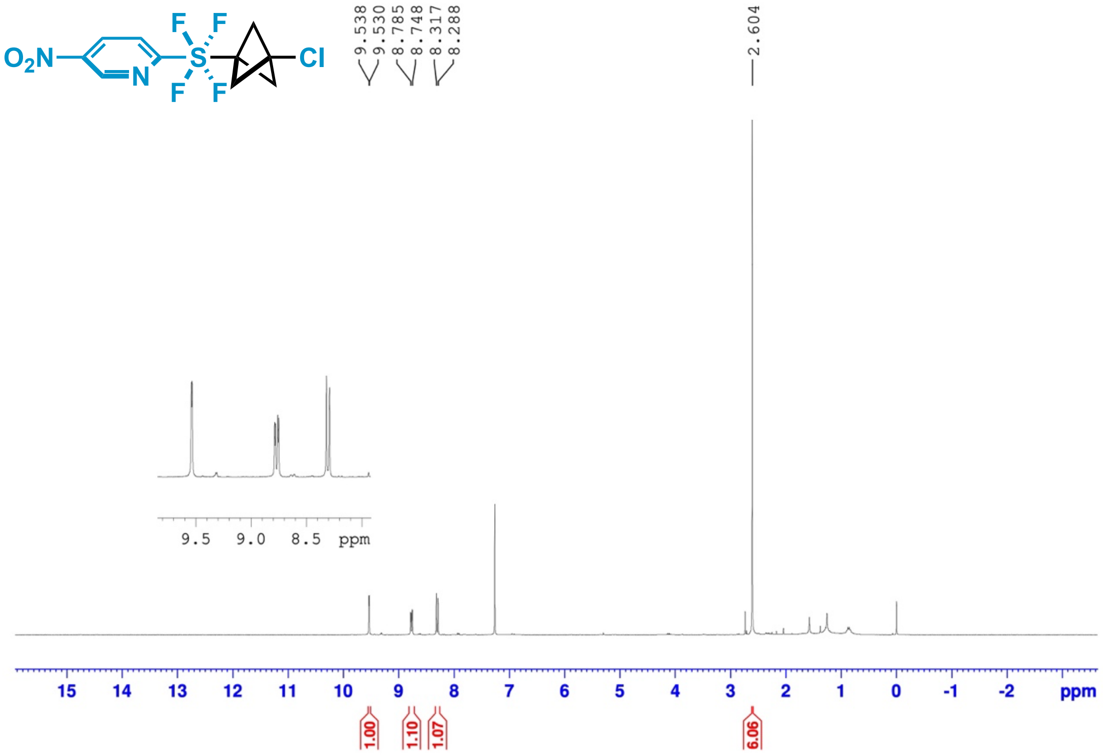

Figure S36: ^{1}H NMR spectrum of 2-((3-chlorobicyclo[1.1.1]pentan-1-yl)tetrafluoro-λ^6-sulfaneyl)-5-nitropyridine (compound **15**).

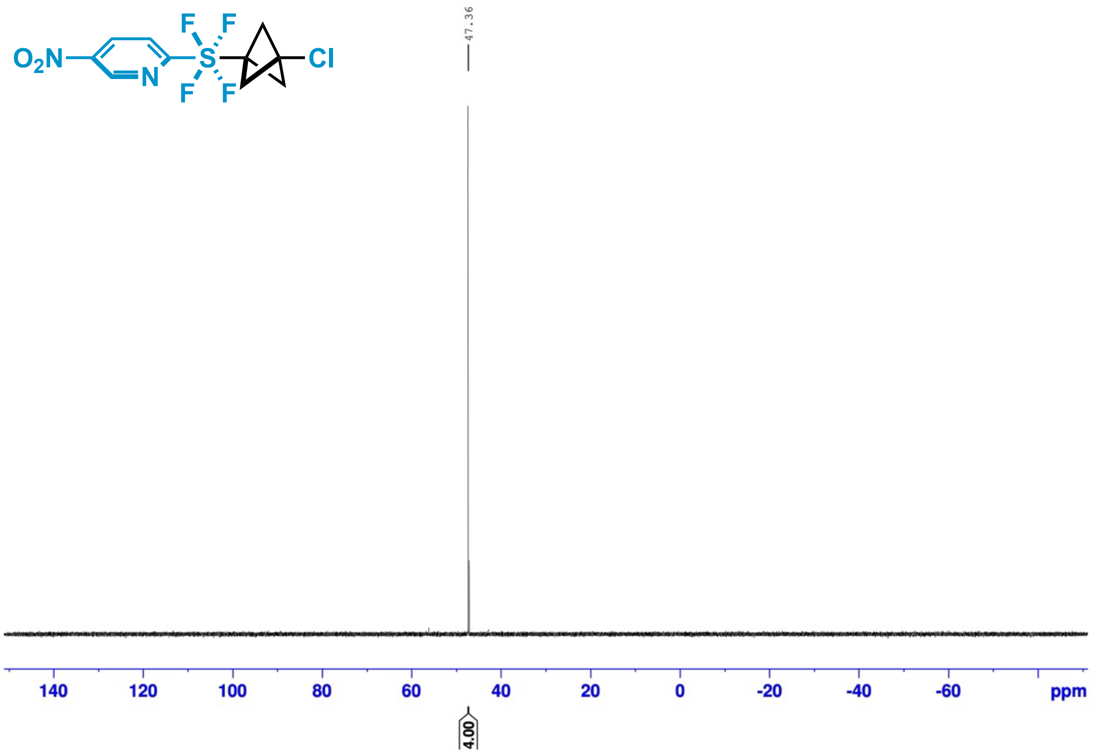

Figure S37: ^{19}F NMR spectrum of 2-((3-chlorobicyclo[1.1.1]pentan-1-yl)tetrafluoro-λ^{6}-sulfaneyl)-5-nitropyridine (compound **15**).

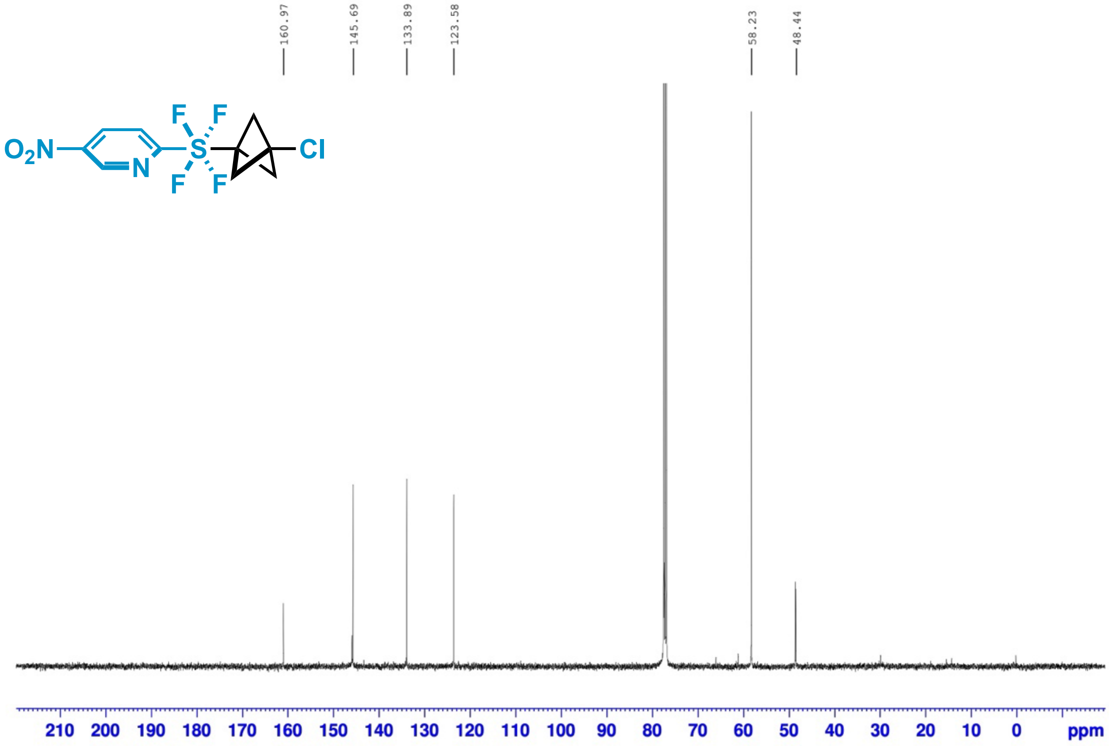

Figure S38: ^{13}C NMR spectrum of 2-((3-chlorobicyclo[1.1.1]pentan-1-yl)tetrafluoro-λ^6-sulfaneyl)-5-nitropyridine (compound **15**).). *Note: the quintets of aryl and alkyl carbon atoms adjacent to the -SF_4- unit could not be resolved.*

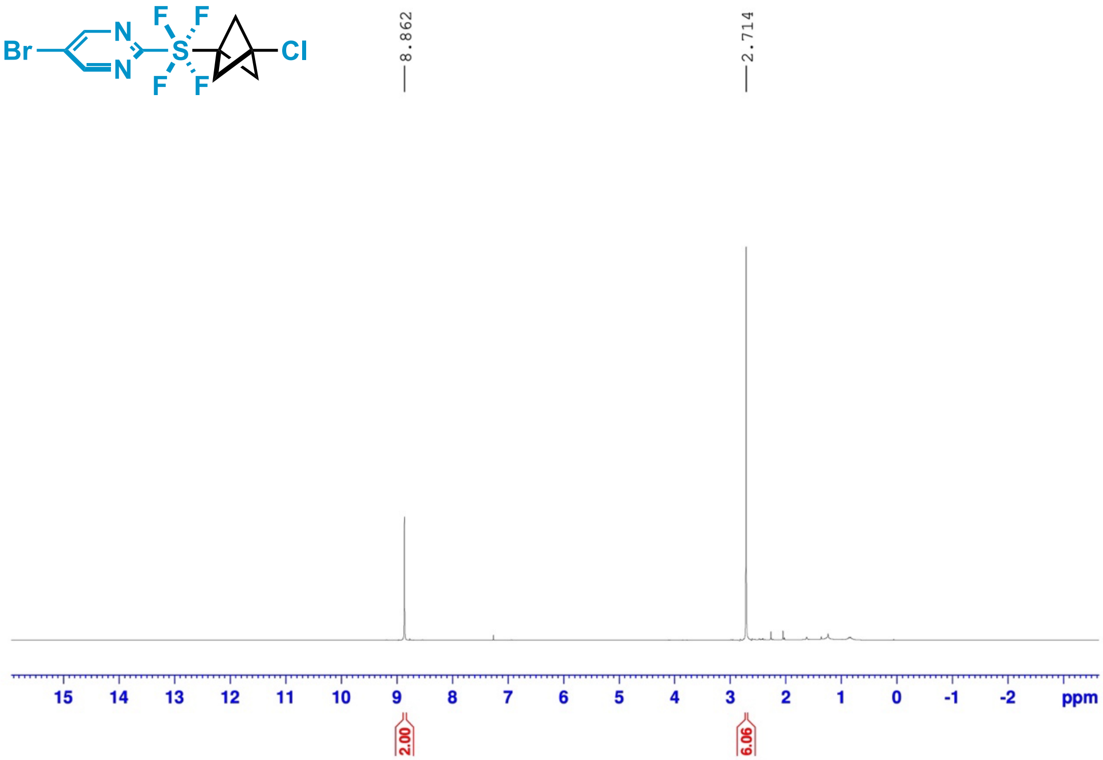

Figure S39: ^{1}H NMR spectrum of 5-bromo-2-((3-chlorobicyclo[1.1.1]pentan-1-yl)tetrafluoro-λ^{6}-sulfaneyl)pyrimidine (compound **16**).

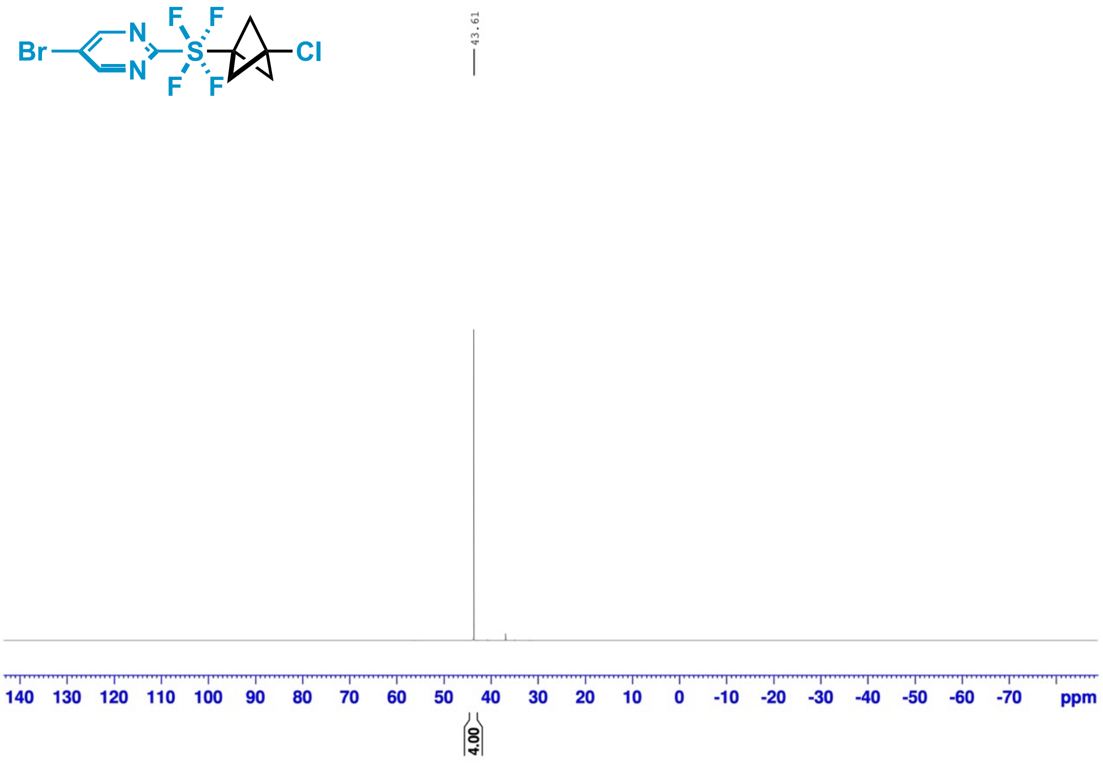

Figure S40: ^{19}F NMR spectrum of 5-bromo-2-((3-chlorobicyclo[1.1.1]pentan-1-yl)tetrafluoro-λ^6-sulfaneyl)pyrimidine (compound **16**).

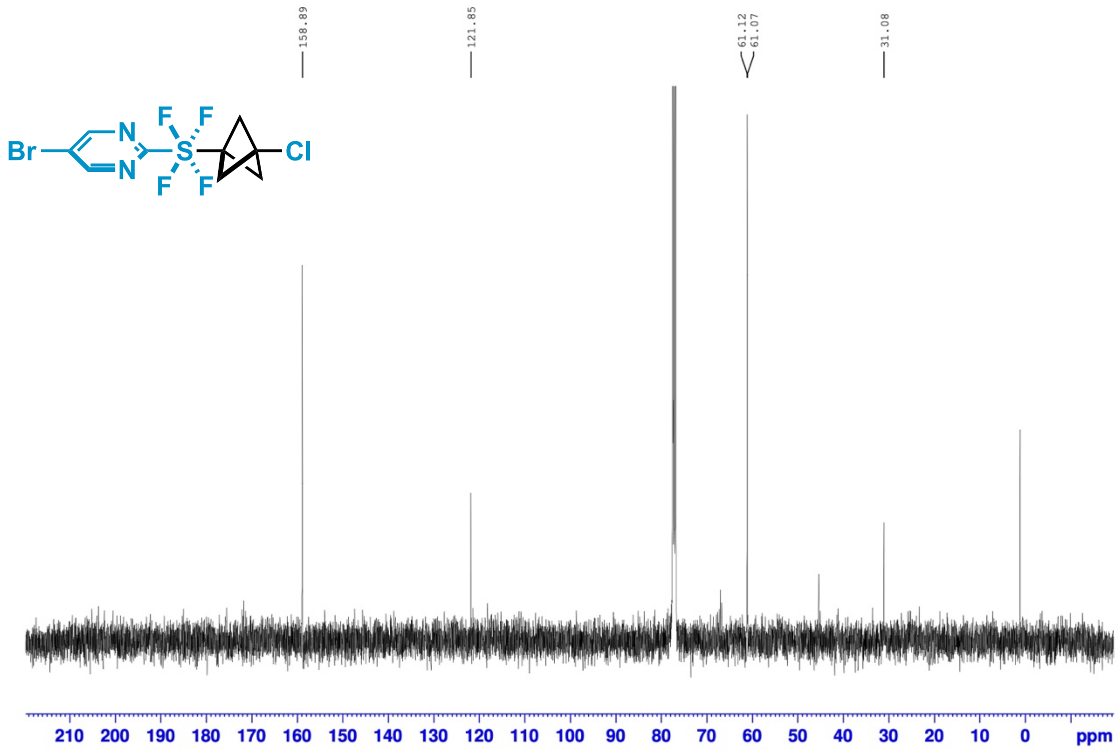

Figure S41: ^{13}C NMR spectrum of 5-bromo-2-((3-chlorobicyclo[1.1.1]pentan-1-yl)tetrafluoro-λ^6-sulfaneyl)pyrimidine (compound **16**). *Note:the quintets of aryl and alkyl carbon atoms adjacent to the -SF$_4$- unit could not be resolved.*

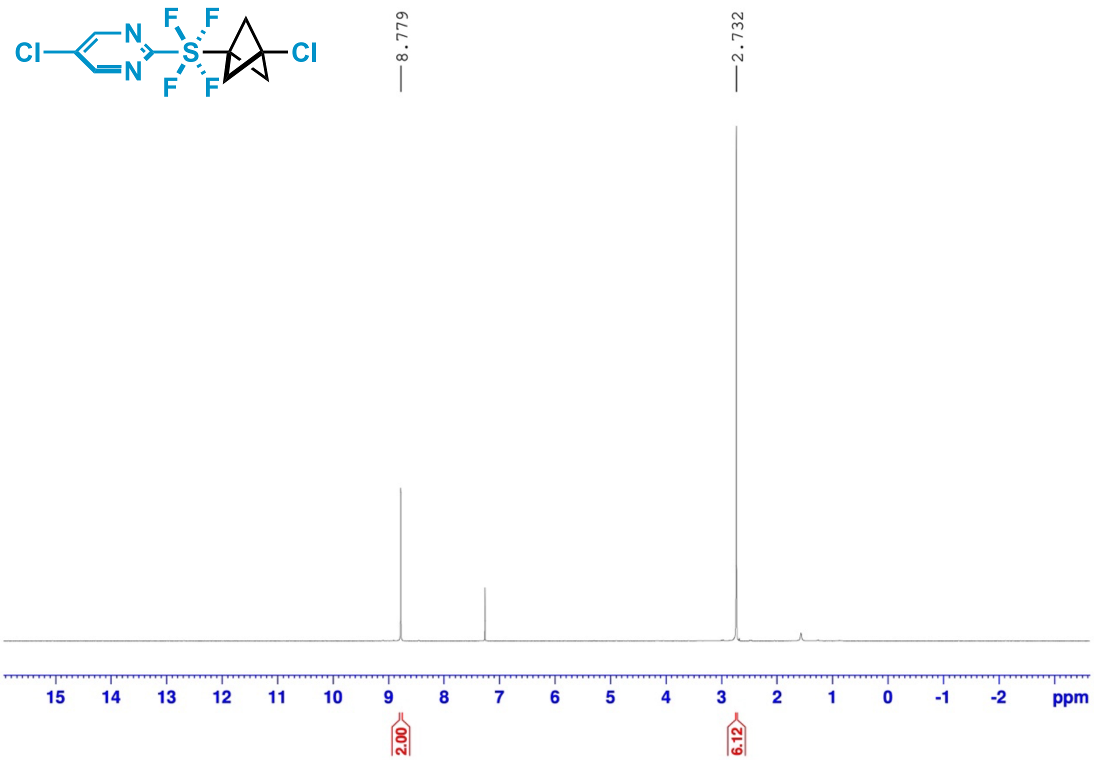

Figure S42: ^{1}H NMR spectrum of 5-chloro-2-((3-chlorobicyclo[1.1.1]pentan-1-yl)tetrafluoro-λ^6-sulfaneyl)pyrimidine (compound **17**).

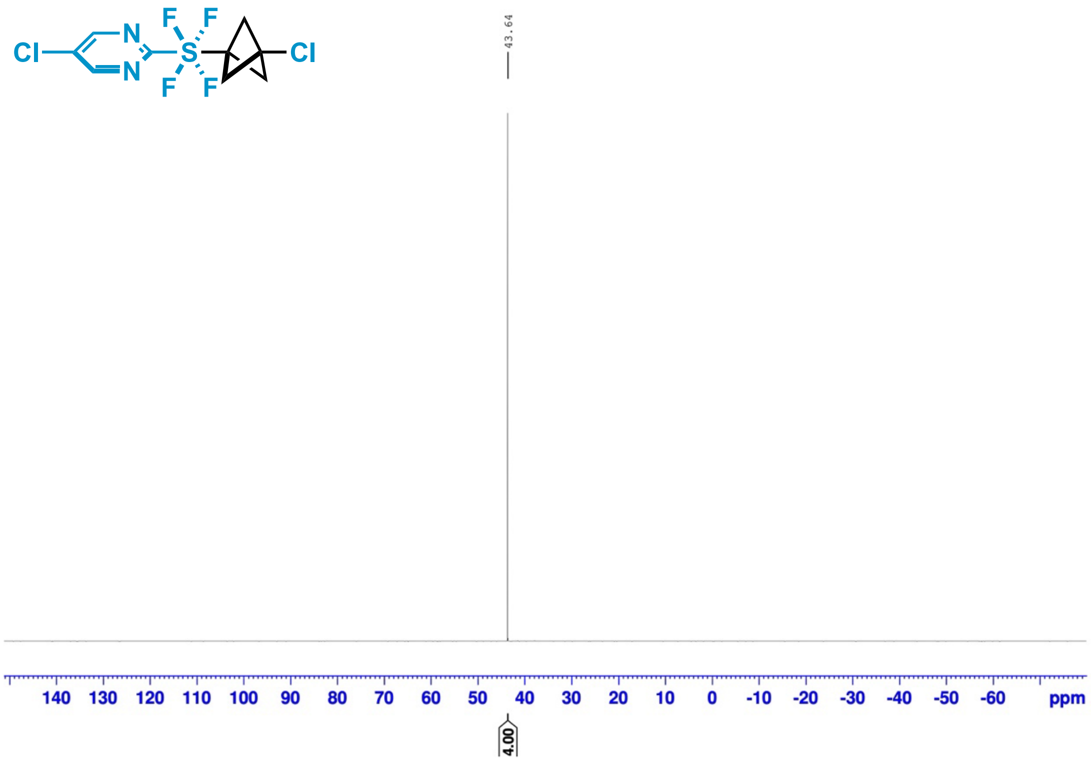

Figure S43: ^{19}F NMR spectrum of 5-chloro-2-((3-chlorobicyclo[1.1.1]pentan-1-yl)tetrafluoro-λ^6-sulfaneyl)pyrimidine (compound **17**).

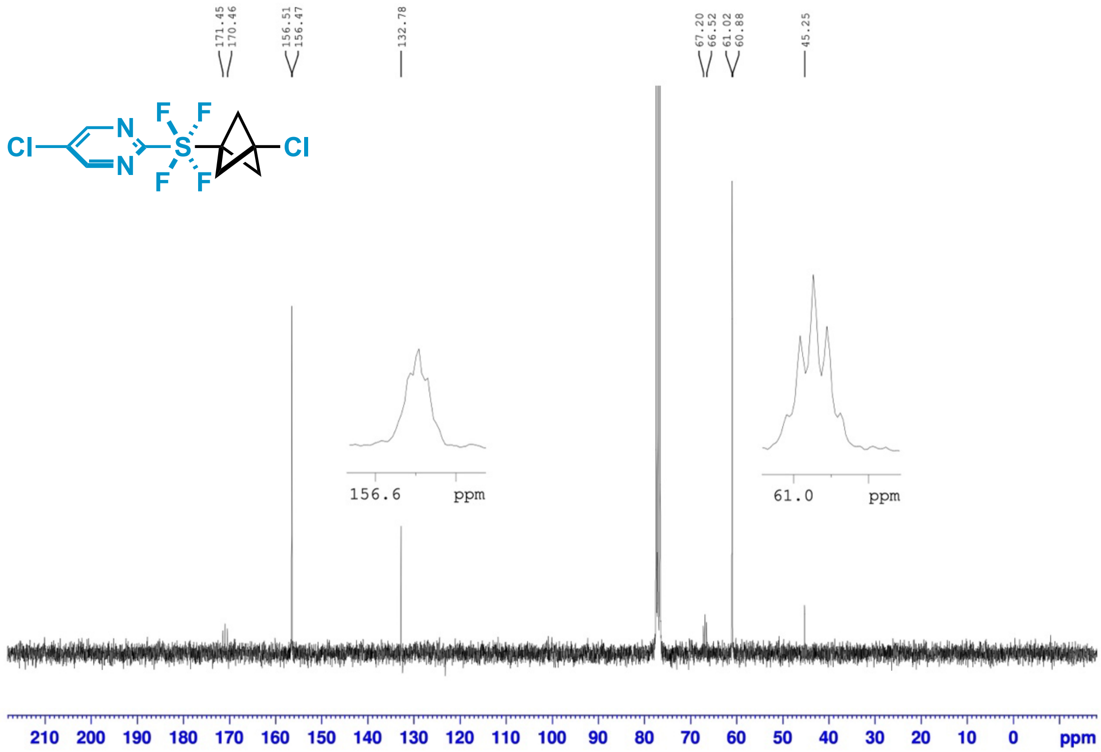

Figure S44: ^{13}C NMR spectrum of 5-chloro-2-((3-chlorobicyclo[1.1.1]pentan-1-yl)tetrafluoro-λ^6-sulfaneyl)pyrimidine (compound **17**).

Computational Methods

The Gaussian 09, Revison E.01[5] and Gaussian 16, Revision A.03[6a] suites were used for all calculations. Geometry optimizations and subsequent vibrational analyses were performed at either ωB97XD/6-311++G** or ωB97XD/cc-pVQZ.[6b] The ωB97XD functional was used to account for long-range and dispersion interactions.[7] Polarization functions were applied to account for effects from ring strain. Minima and were confirmed by means of vibrational analysis using the same functional/basis set combination: no imaginary frequencies were found. Natural bond orbital (NBO) second-order perturbation analyses and determination of Wiberg bond index (WBI) were performed at ωB97XD/cc-pVQZ on coordinates optimized using the same functional/basis set combination. Pictures of molecular orbitals were rendered using the IQmol program.[8]

Table S3. Computed C_1•••C_3, C_1–C_2, and C_2–C_3 distances, as well as $\theta_{C1\text{-}C2\text{-}C3}$ angles and Wiberg bond indexes for C_1•••C_3, for a series of substituted bicyclopentane rings and [1.1.1]propellane at ωB97XD/cc-pVQZ.

	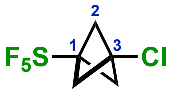	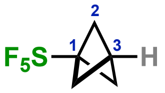	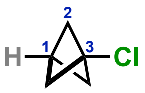	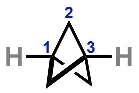	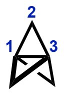
***d*(C1-C3):**	*1.816 Å*	*1.835 Å*	*1.838 Å*	*1.873 Å*	*1.556 Å*
***d*(C1-C2)^a:**	*1.543 Å*	*1.552 Å*	*1.552 Å*	*1.549 Å*	*1.511 Å*
***d*(C2-C3)^a:**	*1.544 Å*	*1.539 Å*	*1.539 Å*	*1.549 Å*	*1.511 Å*
**$\theta_{C1\text{-}C2\text{-}C3}$^a:**	*72.1°*	*72.8°*	*73.0°*	*74.4°*	*62.0°*
***WBI* (C1-C3):**	*0.085*	*0.063*	*0.071*	*0.035*	*0.79*

^a Average values for all three wing C–C bond distances and angles.

Table S4. Computed transannular C•••C and wing C–C distances, as well as $\theta_{C1\text{-}C2\text{-}C3}$ and $\theta_{C6\text{-}C7\text{-}C8}$ angles and Wiberg bond indexes for C_1•••C_3 and C_6•••C_8, for SF_5-BCP-BCP-Cl (**3**) at ωB97XD/cc-pVQZ.

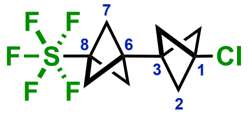

***d*(C8-C6):**	*1.838 Å*	***d*(C1-C3):**	*1.842 Å*
***d*(C8-C7)^a:**	*1.538 Å*	***d*(C1-C2)^a:**	*1.539 Å*
***d*(C7-C6)^a:**	*1.555 Å*	***d*(C2-C3)^a:**	*1.554 Å*
**$\theta_{C8\text{-}C7\text{-}C6}$^a:**	*72.9°*	**$\theta_{C1\text{-}C2\text{-}C3}$^a:**	*73.1°*
***WBI* (C8-C6):**	*0.068*	***WBI* (C1-C3):**	*0.076*

^a Average values for all three wing C–C bond distances and angles.

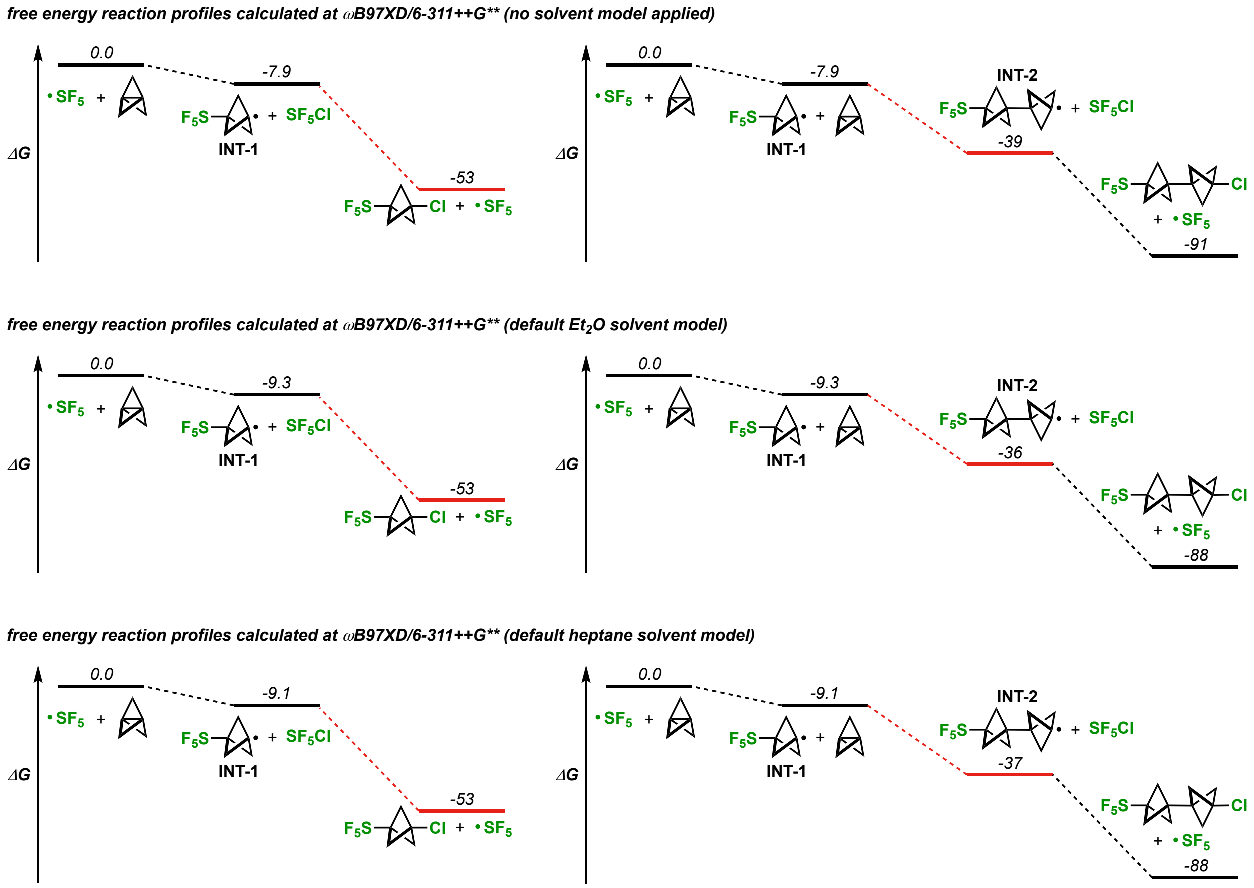

Figure S45. Gibbs free energy reaction profiles of proposed mechanisms to form SF_5-BCP-Cl and SF_5-BCP-BCP-Cl highlighting the computed differences in free energies of the product-determining steps (note: energy of the Cl radical not included in the 0.0). All structures were optimized at ωB97XD/6-311++G** and contain no imaginary frequencies; results from different solvent models (specified in the figure above) are compared.

Coordinates

Calculations at ωB97XD/cc-pVQZ

[1.1.1]propellane (1)

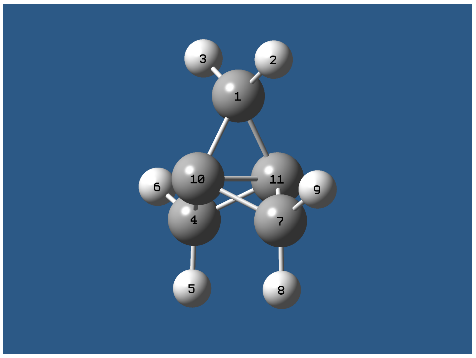

\# opt freq wb97xd/cc-pvqz geom=connectivity
Charge = 0 Multiplicity = 1

Center Number	Atomic Number	Forces (Hartrees/Bohr) X	Y	Z
1	6	0.000172620	-0.000284990	-0.000000000
2	1	-0.000034941	0.000049372	0.000133074
3	1	-0.000034941	0.000049372	-0.000133074
4	6	-0.000182179	0.000071519	0.000317471
5	1	0.000122406	-0.000091016	0.000003384
6	1	-0.000042518	0.000057438	-0.000118649
7	6	-0.000182179	0.000071519	-0.000317471
8	1	0.000122406	-0.000091016	-0.000003384
9	1	-0.000042518	0.000057438	0.000118649
10	6	0.000275457	0.000396408	-0.000000000
11	6	-0.000173614	-0.000286043	0.000000000

Bicyclo[1.1.1]pentane (20)

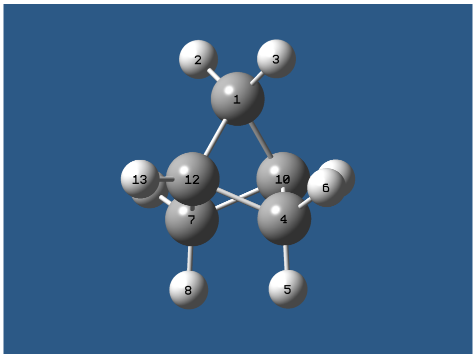

 # opt freq wb97xd/cc-pvqz geom=connectivity
Charge = 0 Multiplicity = 1

Center Number	Atomic Number	Forces (Hartrees/Bohr) X	Y	Z
1	6	0.000000000	-0.000000000	-0.000199189
2	1	-0.000000000	-0.000006936	0.000011563
3	1	0.000000000	0.000006936	0.000011563
4	6	0.000000000	-0.000154726	0.000119100
5	1	0.000000000	0.000033474	0.000019456
6	1	0.000000000	0.000002124	-0.000009271
7	6	-0.000000000	0.000154726	0.000119100
8	1	0.000000000	-0.000033474	0.000019456
9	1	-0.000000000	-0.000002124	-0.000009271
10	6	0.000023865	-0.000000000	-0.000031582
11	1	0.000016382	0.000000000	-0.000009672
12	6	-0.000023865	0.000000000	-0.000031582
13	1	-0.000016382	-0.000000000	-0.000009672

Bicyclo[1.1.1]pentan-1-ylpentafluoro-λ⁶-sulfane (21)

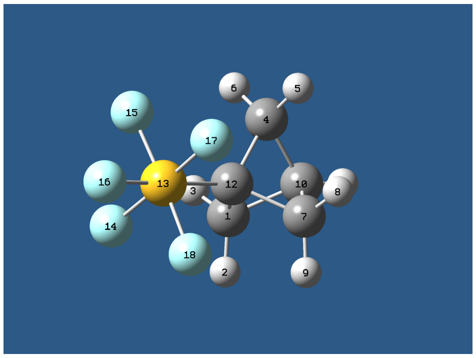

 # opt freq wb97xd/cc-pvqz geom=connectivity
Charge = 0 Multiplicity = 1

Center Number	Atomic Number	Forces (Hartrees/Bohr) X	Y	Z
1	6	-0.000015404	0.000020956	0.000014565
2	1	0.000007709	-0.000006619	0.000011174
3	1	0.000003564	-0.000007541	0.000007407
4	6	-0.000003078	-0.000023193	-0.000056821
5	1	0.000004927	-0.000009874	-0.000018320
6	1	0.000006210	-0.000009073	0.000000708
7	6	0.000003400	0.000046787	-0.000024649
8	1	-0.000006396	0.000001049	0.000002951
9	1	-0.000005945	0.000000090	-0.000007861
10	6	-0.000018469	0.000026426	0.000019977
11	1	-0.000001297	-0.000017282	0.000004383
12	6	0.000049391	0.000008044	-0.000005288
13	16	0.000145520	-0.000138296	0.000078516
14	9	-0.000068550	0.000017306	-0.000009554
15	9	-0.000021037	0.000023022	-0.000002407
16	9	0.000046012	0.000003766	-0.000003908
17	9	-0.000053410	0.000041068	-0.000005448
18	9	-0.000073146	0.000023365	-0.000005426

1-Chlorobicyclo[1.1.1]pentane (22)

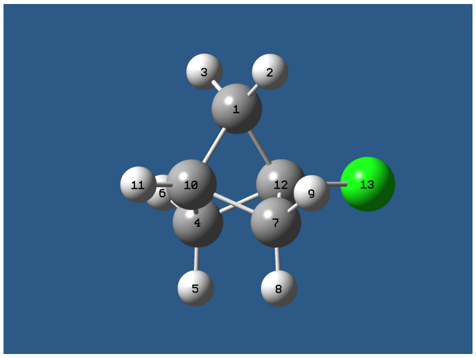

----

opt freq wb97xd/cc-pvqz geom=connectivity

Charge = 0 Multiplicity = 1

Center Number	Atomic Number	Forces (Hartrees/Bohr) X	Y	Z
1	6	0.000099665	0.000187472	-0.000003200
2	1	-0.000007658	-0.000009553	-0.000035165
3	1	-0.000007754	-0.000009631	0.000035784
4	6	0.000111091	-0.000127881	-0.000139778
5	1	-0.000006846	-0.000042324	0.000020265
6	1	-0.000011622	0.000036170	0.000015844
7	6	0.000111095	-0.000130637	0.000138764
8	1	-0.000006668	-0.000042016	-0.000019527
9	1	-0.000011313	0.000035350	-0.000014986
10	6	0.000048600	0.000038700	0.000000948
11	1	-0.000045827	0.000013066	-0.000000078
12	6	-0.000385685	0.000057871	0.000001055
13	17	0.000112923	-0.000006587	0.000000076

(3-Chlorobicyclo[1.1.1]pentan-1-yl)pentafluoro-λ^{6}-sulfane (2)

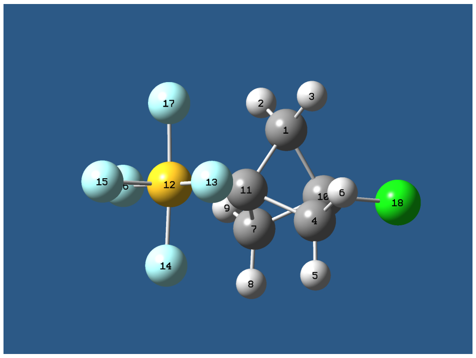

----

opt freq wb97xd/cc-pvqz geom=connectivity

Charge = 0 Multiplicity = 1

Center Number	Atomic Number	Forces (Hartrees/Bohr) X	Y	Z
1	6	-0.000003120	-0.000007759	-0.000004229
2	1	-0.000000256	0.000002340	0.000004272
3	1	-0.000000890	0.000000421	0.000004048
4	6	-0.000002643	0.000004043	-0.000016777
5	1	0.000000864	-0.000000042	0.000001390
6	1	0.000000498	0.000004290	0.000002727
7	6	-0.000004579	0.000009005	-0.000007241
8	1	0.000001951	0.000001319	-0.000003208
9	1	0.000000576	-0.000000670	-0.000007087
10	6	0.000022903	-0.000009509	0.000023217
11	6	0.000005647	-0.000002551	0.000000944
12	16	-0.000033862	0.000025028	-0.000010066
13	9	0.000000033	-0.000002204	-0.000020399
14	9	-0.000011117	0.000032182	0.000011704
15	9	0.000027988	-0.000016155	-0.000007781
16	9	-0.000001184	-0.000020994	0.000034154
17	9	0.000004886	-0.000020129	-0.000005388
18	17	-0.000007695	0.000001386	-0.000000279

(3'-chloro-[1,1'-bi(bicyclo[1.1.1]pentan)]-3-yl)pentafluoro-λ⁶-sulfane (3)

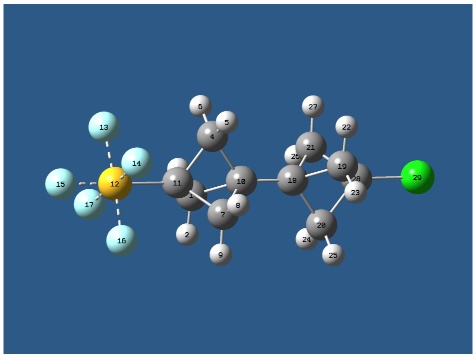

\# opt freq wb97xd/cc-pvqz geom=connectivity
Charge = 0 Multiplicity = 1

Center Number	Atomic Number	Forces (Hartrees/Bohr) X	Y	Z
1	6	-0.000174467	-0.000057058	-0.000022567
2	1	0.000004138	0.000018333	0.000000098
3	1	0.000003786	0.000015873	0.000009726
4	6	-0.000175438	0.000001695	0.000053091
5	1	0.000004319	0.000000206	-0.000015172
6	1	0.000005384	-0.000003052	-0.000019151
7	6	-0.000172713	0.000032301	-0.000032693
8	1	0.000003199	-0.000015493	0.000015627
9	1	0.000004661	-0.000019626	0.000009543
10	6	0.000258962	0.000011865	0.000001034
11	6	0.000339816	0.000016148	0.000000080
12	16	-0.000243183	-0.000028662	-0.000020255
13	9	0.000009551	-0.000003372	-0.000029327
14	9	0.000025666	0.000001194	0.000005564
15	9	0.000111119	-0.000019736	-0.000011146
16	9	0.000008526	-0.000020565	0.000033495
17	9	0.000013370	0.000063874	0.000023431
18	6	-0.000052083	-0.000008820	0.000008443
19	6	0.000012777	0.000008499	-0.000009150
20	6	0.000012811	0.000004181	-0.000015704
21	6	0.000013055	0.000004870	0.000014062
22	1	0.000000152	-0.000002919	0.000000967
23	1	-0.000001069	-0.000004059	-0.000000167
24	1	-0.000001168	0.000003463	0.000001243
25	1	-0.000001372	-0.000000449	0.000003997
26	1	0.000000233	0.000007019	-0.000002993
27	1	-0.000000898	0.000005829	-0.000002829
28	6	-0.000008106	-0.000012295	0.000000956
29	17	-0.000001029	0.000000755	-0.000000204

*Calculations at ωB97XD/6-311++G***

Pentafluorosulfanyl radical

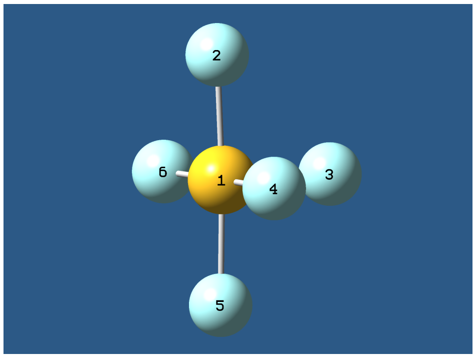

 # opt freq wb97xd/6-311+g(d,p) geom=connectivity
Charge = 0 Multiplicity = 2

Center Number	Atomic Number	Forces (Hartrees/Bohr) X	Y	Z
1	16	0.000045207	-0.000000000	0.000000000
2	9	0.000021885	0.000052242	0.000000000
3	9	-0.000132748	0.000000000	-0.000000000
4	9	0.000021885	0.000000000	0.000052242
5	9	0.000021885	-0.000052242	0.000000000
6	9	0.000021885	-0.000000000	-0.000052242

Using Default Et_2O Solvent Model

\# opt freq uwb97xd/6-311+g(d,p) scrf=(solvent=diethylether) geom=connectivity
Charge = 0 Multiplicity = 2

Center Number	Atomic Number	Forces (Hartrees/Bohr) X	Y	Z
1	16	-0.000000000	-0.000000000	-0.000321379
2	9	0.000000000	0.000193735	0.000052508
3	9	-0.000000000	-0.000000000	0.000111345
4	9	0.000193735	0.000000000	0.000052508
5	9	0.000000000	-0.000193735	0.000052508
6	9	-0.000193735	-0.000000000	0.000052508

Using Default Heptane Solvent Model

\# opt freq uwb97xd/6-311+g(d,p) scrf=(solvent=heptane) geom=connectivity
Charge = 0 Multiplicity = 2

Center Number	Atomic Number	Forces (Hartrees/Bohr) X	Y	Z
1	16	-0.000000000	-0.000000000	-0.000236747
2	9	0.000000000	0.000114537	0.000033006
3	9	-0.000000000	-0.000000000	0.000104722
4	9	0.000114537	0.000000000	0.000033006
5	9	0.000000000	-0.000114537	0.000033006
6	9	-0.000114537	-0.000000000	0.000033006

[1.1.1]propellane (1)

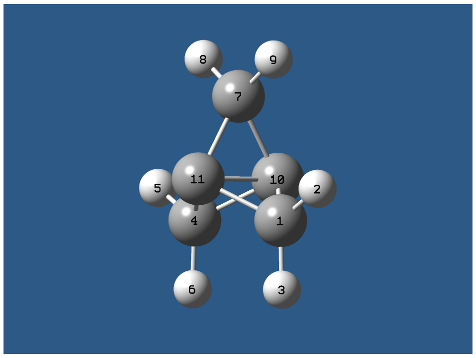

# opt freq wb97xd/6-311+g(d,p) geom=connectivity
Charge = 0 Multiplicity = 1

Center Number	Atomic Number	Forces (Hartrees/Bohr) X	Y	Z
1	6	0.000297941	-0.000000000	0.000033385
2	1	-0.000005364	0.000001010	-0.000001701
3	1	-0.000005364	-0.000001010	-0.000001701
4	6	0.000010964	0.000011736	-0.000018138
5	1	0.000104175	-0.000001348	-0.000004383
6	1	0.000039967	0.000005119	-0.000001152
7	6	0.000010964	-0.000011736	-0.000018138
8	1	0.000104175	0.000001348	-0.000004383
9	1	0.000039967	-0.000005119	-0.000001152
10	6	-0.000250650	-0.000000000	0.000560104
11	6	-0.000346776	0.000000000	-0.000542742

Using Default Et_2O Solvent Model

 # opt freq uwb97xd/6-311+g(d,p) scrf=(solvent=diethylether) geom=connectivity
Charge = 0 Multiplicity = 1

Center Number	Atomic Number	Forces (Hartrees/Bohr) X	Y	Z
1	6	-0.000105549	0.000333479	-0.000000000
2	1	-0.000176462	0.000122104	0.000015129
3	1	-0.000176462	0.000122104	-0.000015129
4	6	0.000197200	-0.000115986	0.000305734
5	1	0.000071714	-0.000048813	0.000155865
6	1	0.000056612	-0.000070653	0.000128859
7	6	0.000197200	-0.000115986	-0.000305734
8	1	0.000071714	-0.000048813	-0.000155865
9	1	0.000056612	-0.000070653	-0.000128859
10	6	-0.000181502	-0.000259613	0.000000000
11	6	-0.000011075	0.000152831	0.000000000

Using Default Heptane Solvent Model

 # opt freq uwb97xd/6-311+g(d,p) scrf=(solvent=heptane) geom=connectivity
Charge = 0 Multiplicity = 1

Center Number	Atomic Number	Forces (Hartrees/Bohr) X	Y	Z
1	6	0.000011819	0.000243457	-0.000000000
2	1	-0.000108617	0.000060600	0.000006712
3	1	-0.000108617	0.000060600	-0.000006712
4	6	0.000138516	-0.000072312	0.000174513
5	1	0.000041130	-0.000023039	0.000072460
6	1	0.000018761	-0.000036759	0.000050582
7	6	0.000138516	-0.000072312	-0.000174513
8	1	0.000041130	-0.000023039	-0.000072460
9	1	0.000018761	-0.000036759	-0.000050582
10	6	-0.000106009	-0.000162339	0.000000000
11	6	-0.000085391	0.000061900	-0.000000000

Pentafluorosulfanyl chloride

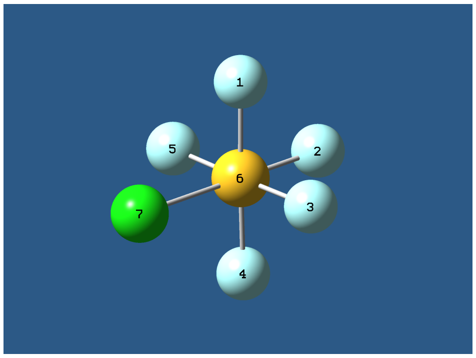

# opt freq wb97xd/6-311+g(d,p) geom=connectivity
Charge = 0 Multiplicity = 1

Center Number	Atomic Number	Forces (Hartrees/Bohr) X	Y	Z
1	9	0.000000000	0.000165843	-0.000039819
2	9	0.000000000	0.000000000	-0.000085934
3	9	0.000165843	0.000000000	-0.000039819
4	9	0.000000000	-0.000165843	-0.000039819
5	9	-0.000165843	-0.000000000	-0.000039819
6	16	0.000000000	0.000000000	-0.000020545
7	17	0.000000000	0.000000000	0.000265755

Using Default Et_2O Solvent Model

 # opt freq uwb97xd/6-311+g(d,p) scrf=(solvent=diethylether) geom=connectivity
Charge = 0 Multiplicity = 1

Center Number	Atomic Number	Forces (Hartrees/Bohr) X	Y	Z
1	9	-0.000000000	0.000010883	0.000046138
2	9	-0.000000000	0.000000000	-0.000341044
3	9	0.000010883	0.000000000	0.000046138
4	9	0.000000000	-0.000010883	0.000046138
5	9	-0.000010883	-0.000000000	0.000046138
6	16	-0.000000000	0.000000000	0.000521645
7	17	-0.000000000	0.000000000	-0.000365152

Using Default Heptane Solvent Model

 # opt freq uwb97xd/6-311+g(d,p) scrf=(solvent=heptane) geom=connectivity
Charge = 0 Multiplicity = 1

Center Number	Atomic Number	Forces (Hartrees/Bohr) X	Y	Z
1	9	0.000000000	-0.000010929	0.000021015
2	9	-0.000000000	0.000000000	-0.000129702
3	9	-0.000010929	0.000000000	0.000021015
4	9	0.000000000	0.000010929	0.000021015
5	9	0.000010929	0.000000000	0.000021015
6	16	-0.000000000	-0.000000000	0.000237651
7	17	-0.000000000	-0.000000000	-0.000192011

1-Pentafluoro-λ[6]-sulfanyl-bicyclo[1.1.1]pentan-3-yl radical (INT-1)

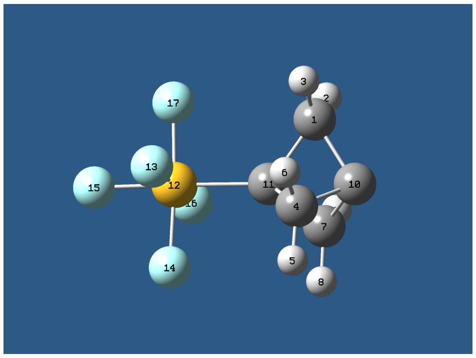

\# opt freq wb97xd/6-311+g(d,p) geom=connectivity
Charge = 0 Multiplicity = 2

Center Number	Atomic Number	Forces (Hartrees/Bohr) X	Y	Z
1	6	0.000060114	-0.000045379	-0.000328956
2	1	-0.000013150	0.000007861	0.000033339
3	1	0.000009726	-0.000085070	0.000157228
4	6	-0.000005295	-0.000165539	0.000355060
5	1	0.000017420	-0.000005421	-0.000090474
6	1	0.000039293	0.000157631	-0.000195159
7	6	0.000227446	0.000062140	0.000040358
8	1	-0.000044585	0.000019136	0.000007212
9	1	-0.000096176	-0.000127307	0.000014982
10	6	-0.000288483	0.000154984	0.000021725
11	6	0.000223054	0.000007581	0.000061678
12	16	-0.000110749	-0.000004668	-0.000012997
13	9	0.000000350	0.000051357	-0.000001935
14	9	-0.000014760	0.000005562	-0.000008727
15	9	-0.000030903	-0.000027821	-0.000021982
16	9	-0.000001185	-0.000035057	0.000017209
17	9	0.000027883	0.000030010	-0.000048560

Using Default Et_2O Solvent Model

 # opt freq uwb97xd/6-311+g(d,p) scrf=(solvent=diethylether) geom=connectivity
Charge = 0 Multiplicity = 2

Center Number	Atomic Number	Forces (Hartrees/Bohr) X	Y	Z
1	6	0.000027093	0.000096028	0.000026550
2	1	-0.000002139	0.000034290	-0.000029028
3	1	-0.000012539	0.000003835	0.000051416
4	6	0.000000871	-0.000078617	-0.000052524
5	1	0.000014343	0.000060777	-0.000021125
6	1	0.000000820	-0.000015759	-0.000013019
7	6	0.000036066	-0.000068326	0.000019487
8	1	-0.000036229	0.000000926	0.000026043
9	1	-0.000024793	-0.000038067	0.000043809
10	6	-0.000043630	0.000013316	-0.000012803
11	6	0.000125093	-0.000016393	-0.000007321
12	16	0.000132819	-0.000012425	0.000022885
13	9	0.000053249	0.000042496	-0.000050346
14	9	0.000036422	-0.000060047	-0.000041145
15	9	-0.000404345	0.000009424	0.000022127
16	9	0.000028653	0.000003066	0.000041798
17	9	0.000068247	0.000025478	-0.000026804

Using Default Heptane Solvent Model

\# opt freq uwb97xd/6-311+g(d,p) scrf=(solvent=heptane) geom=connectivity
Charge = 0 Multiplicity = 2

Center Number	Atomic Number	Atomic Type	Coordinates (Angstroms) X	Y	Z
1	6	0	1.909043	0.011790	-1.273807
2	1	0	1.898963	-0.893958	-1.873472
3	1	0	1.886563	0.932625	-1.850222
4	6	0	1.909690	1.093957	0.656463
5	1	0	1.899888	1.133608	1.742022
6	1	0	1.886860	2.065971	0.171371
7	6	0	1.919035	-1.119242	0.628297
8	1	0	1.909406	-1.187005	1.712778
9	1	0	1.908276	-2.079192	0.119238
10	6	0	2.769218	0.000066	0.000699
11	6	0	1.060526	-0.009479	0.007283
12	16	0	-0.853773	-0.002244	0.001754
13	9	0	-0.871731	1.400092	-0.864362
14	9	0	-0.920937	0.859245	1.402982
15	9	0	-2.464269	0.033040	-0.026344
16	9	0	-0.958207	-1.398611	0.865357
17	9	0	-0.910929	-0.871399	-1.395789

(3-Chlorobicyclo[1.1.1]pentan-1-yl)pentafluoro-λ^6-sulfane (2)

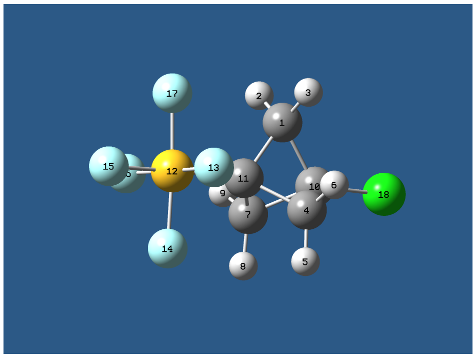

# opt freq wb97xd/6-311+g(d,p) geom=connectivity
Charge = 0 Multiplicity = 1

Center Number	Atomic Number	Forces (Hartrees/Bohr) X	Y	Z
1	6	-0.000013408	0.000025928	0.000020168
2	1	-0.000031022	0.000012660	-0.000015435
3	1	-0.000034696	0.000040460	-0.000016094
4	6	-0.000053274	0.000063218	0.000032171
5	1	0.000045752	0.000019339	0.000031404
6	1	-0.000025962	-0.000075722	-0.000026745
7	6	-0.000088961	-0.000030083	0.000011933
8	1	0.000006113	-0.000020393	-0.000002324
9	1	-0.000010647	0.000049737	-0.000002944
10	6	0.000079626	-0.000046911	0.000013771
11	6	0.000145365	-0.000030343	-0.000025044
12	16	-0.000045511	-0.000017037	0.000078679
13	9	0.000016926	0.000001980	-0.000017136
14	9	-0.000006038	0.000010584	0.000006614
15	9	0.000039085	0.000007079	-0.000080318
16	9	-0.000028786	0.000013147	-0.000003168
17	9	0.000010368	-0.000040512	-0.000011435
18	17	-0.000004929	0.000016870	0.000005903

Using Default Et_2O Solvent Model

 # opt freq uwb97xd/6-311+g(d,p) scrf=(solvent=diethylether) geom=connectivity
Charge = 0 Multiplicity = 1

Center Number	Atomic Number	Atomic Type	Coordinates (Angstroms) X	Y	Z
1	6	0	-1.200992	0.817635	0.948675
2	1	0	-1.201788	0.522550	1.995989
3	1	0	-1.192123	1.895712	0.798733
4	6	0	-1.200433	0.398857	-1.185657
5	1	0	-1.200660	-0.270124	-2.043770
6	1	0	-1.191566	1.453480	-1.454458
7	6	0	-1.207448	-1.240220	0.244191
8	1	0	-1.206127	-2.000546	-0.534765
9	1	0	-1.208011	-1.649839	1.252549
10	6	0	-2.108200	-0.003862	0.001387
11	6	0	-0.306068	-0.012887	0.003977
12	16	0	1.507613	-0.001816	0.000654
13	9	0	1.536062	1.585284	-0.372280
14	9	0	1.574825	-0.379515	-1.582631
15	9	0	3.127256	0.040416	-0.014061
16	9	0	1.620467	-1.582371	0.373360
17	9	0	1.584139	0.366574	1.585392
18	17	0	-3.868671	0.002776	-0.000482

Using Default Heptane Solvent Model

# opt freq uwb97xd/6-311+g(d,p) scrf=(solvent=heptane) geom=connectivity
Charge = 0 Multiplicity = 1

Center Number	Atomic Number	Atomic Type	Coordinates (Angstroms) X	Y	Z
1	6	0	-1.197689	0.060369	-1.245306
2	1	0	-1.192085	0.998753	-1.796374
3	1	0	-1.191283	-0.820809	-1.884013
4	6	0	-1.203007	-1.115341	0.582249
5	1	0	-1.204632	-1.230490	1.664504
6	1	0	-1.196315	-2.062248	0.045130
7	6	0	-1.203643	1.055909	0.685988
8	1	0	-1.205262	1.067499	1.774301
9	1	0	-1.197574	2.049635	0.241355
10	6	0	-2.108244	0.000096	0.004206
11	6	0	-0.303296	0.000419	0.012474
12	16	0	1.510186	0.000091	0.001918
13	9	0	1.544251	-1.114396	-1.185958
14	9	0	1.603853	-1.185463	1.112874
15	9	0	3.125439	-0.001272	-0.042805
16	9	0	1.607574	1.106476	1.191283
17	9	0	1.548090	1.193569	-1.106227
18	17	0	-3.867259	-0.000160	-0.002103

1-Pentafluoro-λ^6-sulfanyl-[1,1'-bi(bicyclo[1.1.1]pentan)]-3'-yl radical (INT-2)

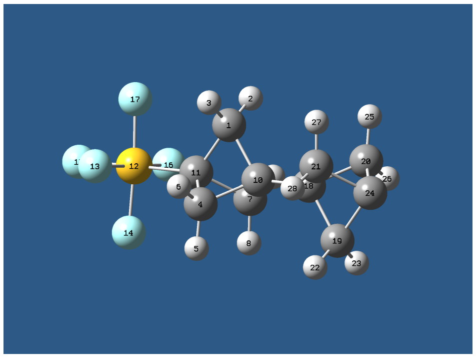

# opt freq wb97xd/6-311+g(d,p) geom=connectivity
Charge = 0 Multiplicity = 2

Center Number	Atomic Number	Forces (Hartrees/Bohr) X	Y	Z
1	6	0.000000143	-0.000094423	-0.000076118
2	1	-0.000008915	-0.000032064	0.000005372
3	1	-0.000012469	-0.000068424	-0.000033258
4	6	0.000016101	-0.000036909	0.000090957
5	1	-0.000008028	-0.000020866	-0.000044887
6	1	-0.000018713	0.000017183	0.000088462
7	6	-0.000020283	-0.000017153	0.000121079
8	1	0.000011219	0.000005511	0.000058500
9	1	0.000004773	0.000004807	-0.000007373
10	6	-0.000038552	0.000079570	-0.000106026
11	6	0.000022004	0.000139869	-0.000126182
12	16	0.000004776	0.000016070	-0.000017530
13	9	0.000022385	-0.000025627	0.000003361
14	9	-0.000017937	-0.000033262	0.000065969
15	9	0.000047454	0.000027559	0.000000909
16	9	0.000002187	0.000027279	0.000009090
17	9	0.000012547	-0.000001514	-0.000018049
18	6	-0.000047997	-0.000084360	0.000093567
19	6	0.000007352	0.000055579	0.000083061
20	6	-0.000009074	0.000041711	-0.000116900
21	6	-0.000017498	0.000022216	-0.000152889
22	1	-0.000007605	0.000041507	-0.000011771
23	1	0.000002137	0.000059203	0.000042646
24	6	0.000014739	-0.000107146	0.000160242
25	1	0.000009886	0.000010350	0.000048531
26	1	0.000017470	-0.000016507	-0.000090201
27	1	0.000007121	-0.000000756	-0.000079309
28	1	0.000004777	-0.000009402	0.000008747

Using Default Et_2O Solvent Model

# opt freq uwb97xd/6-311+g(d,p) scrf=(solvent=diethylether) geom=connectivity
Charge = 0 Multiplicity = 2

Center Number	Atomic Number	Atomic Type	Coordinates (Angstroms) X	Y	Z
1	6	0	-0.474253	0.683597	1.044644
2	1	0	-0.470350	0.257324	2.046608
3	1	0	-0.461387	1.772794	1.040629
4	6	0	-0.473197	0.549191	-1.120187
5	1	0	-0.468245	0.001752	-2.061409
6	1	0	-0.460318	1.630319	-1.251924
7	6	0	-0.480302	-1.259168	0.078841
8	1	0	-0.474679	-1.914820	-0.791133
9	1	0	-0.476236	-1.800771	1.023914
10	6	0	-1.405258	-0.004886	0.000196
11	6	0	0.413280	-0.013726	0.002008
12	16	0	2.243039	-0.002086	0.000417
13	9	0	2.276517	1.626857	-0.155916
14	9	0	2.318187	-0.163428	-1.625084
15	9	0	3.865421	0.043551	-0.007753
16	9	0	2.364648	-1.623794	0.157338
17	9	0	2.325146	0.150461	1.626242
18	6	0	-2.897312	-0.000650	-0.000194
19	6	0	-3.809643	-0.696686	-1.050078
20	6	0	-3.808714	-0.558957	1.129631
21	6	0	-3.804539	1.260046	-0.079632
22	1	0	-3.800052	-0.272901	-2.054506
23	1	0	-3.803569	-1.786907	-1.047307
24	6	0	-4.699076	0.003829	0.000338
25	1	0	-3.798134	-0.011743	2.072504
26	1	0	-3.802515	-1.640686	1.265103
27	1	0	-3.793769	1.918335	0.789410
28	1	0	-3.794553	1.802329	-1.025406

Using Default Heptane Solvent Model

# opt freq uwb97xd/6-311+g(d,p) scrf=(solvent=heptane) geom=connectivity
Charge = 0 Multiplicity = 2

Center Number	Atomic Number	Atomic Type	Coordinates (Angstroms) X	Y	Z
1	6	0	-0.477059	1.197101	0.396659
2	1	0	-0.471004	1.483183	1.447844
3	1	0	-0.470214	2.052421	-0.278174
4	6	0	-0.470666	-0.246199	-1.220405
5	1	0	-0.456130	-1.258413	-1.622848
6	1	0	-0.464226	0.521491	-1.992803
7	6	0	-0.471291	-0.925252	0.837520
8	1	0	-0.456890	-1.978277	0.558546
9	1	0	-0.465609	-0.768232	1.915194
10	6	0	-1.403838	0.004951	0.003078
11	6	0	0.418521	0.012776	0.006488
12	16	0	2.245410	0.001739	0.000754
13	9	0	2.313170	0.563293	-1.532193
14	9	0	2.271778	-1.535660	-0.556642
15	9	0	3.863322	-0.040933	-0.019716
16	9	0	2.322123	-0.550540	1.536275
17	9	0	2.361259	1.532316	0.556404
18	6	0	-2.895754	0.000841	0.000741
19	6	0	-3.803385	-1.197197	-0.399763
20	6	0	-3.810443	0.250898	1.233796
21	6	0	-3.808183	0.941557	-0.837100
22	1	0	-3.793054	-1.480937	-1.452465
23	1	0	-3.794954	-2.056051	0.271866
24	6	0	-4.699217	-0.003962	-0.002838
25	1	0	-3.806638	1.261964	1.641890
26	1	0	-3.802517	-0.518916	2.005881
27	1	0	-3.804094	1.995159	-0.556495
28	1	0	-3.798206	0.789414	-1.916709

(3'-chloro-[1,1'-bi(bicyclo[1.1.1]pentan)]-3-yl)pentafluoro-λ⁶-sulfane (3)

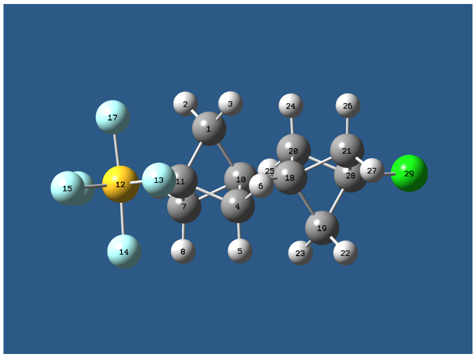

\# opt freq wb97xd/6-311+g(d,p) geom=connectivity
Charge = 0 Multiplicity = 1

Center Number	Atomic Number	Forces (Hartrees/Bohr) X	Y	Z
1	6	-0.000005809	-0.000014342	0.000069761
2	1	0.000001305	-0.000015662	-0.000015175
3	1	0.000013903	-0.000087542	0.000092567
4	6	-0.000007134	-0.000030102	0.000049729
5	1	0.000000580	-0.000007304	0.000083081
6	1	0.000010743	-0.000000928	-0.000116665
7	6	0.000033761	-0.000014929	-0.000037118
8	1	-0.000018695	-0.000040153	-0.000084250
9	1	-0.000002184	-0.000005801	0.000004964
10	6	-0.000090059	0.000108271	-0.000019615
11	6	0.000008638	0.000104712	-0.000004956
12	16	-0.000026142	0.000004616	-0.000010758
13	9	-0.000020811	-0.000006858	0.000031563
14	9	0.000016441	-0.000046458	-0.000027049
15	9	-0.000023102	0.000016287	-0.000000875
16	9	0.000015467	0.000020197	-0.000011950
17	9	-0.000012327	0.000016771	-0.000000010
18	6	0.000247655	-0.000112896	0.000026836
19	6	0.000018585	-0.000041333	-0.000034971
20	6	0.000021272	0.000043563	-0.000118775
21	6	-0.000001647	0.000094392	0.000081658
22	1	-0.000005927	0.000006819	0.000007587
23	1	-0.000019273	0.000087464	-0.000086181
24	1	0.000003279	-0.000007104	-0.000084415
25	1	-0.000020463	0.000003644	0.000122785
26	1	0.000002743	0.000034039	0.000096921
27	1	-0.000005067	0.000009613	-0.000017244
28	6	-0.000057281	-0.000116086	0.000004028
29	17	-0.000078448	-0.000002890	-0.000001473

Using Default Et_2O Solvent Model

\# opt freq uwb97xd/6-311+g(d,p) scrf=(solvent=diethylether) geom=connectivity
Charge = 0 Multiplicity = 1

Center Number	Atomic Number	Atomic Type	Coordinates (Angstroms) X	Y	Z
1	6	0	0.261420	1.133247	0.558912
2	1	0	0.265981	1.271622	1.639363
3	1	0	0.267828	2.072558	0.007305
4	6	0	0.267072	-0.074832	-1.242156
5	1	0	0.279426	-1.022237	-1.779391
6	1	0	0.272879	0.792121	-1.901075
7	6	0	0.265848	-1.030943	0.704037
8	1	0	0.278536	-2.035321	0.282519
9	1	0	0.269899	-1.022989	1.792920
10	6	0	-0.666886	0.006385	0.004761
11	6	0	1.154887	0.012397	0.009583
12	16	0	2.982492	0.000762	0.000709
13	9	0	3.053340	0.803845	-1.421848
14	9	0	3.012690	-1.426839	-0.798835
15	9	0	4.604639	-0.038380	-0.029943
16	9	0	3.067791	-0.794175	1.426463
17	9	0	3.106121	1.421258	0.797874
18	6	0	-2.146572	0.003161	0.002321
19	6	0	-3.076219	-1.118117	-0.549478
20	6	0	-3.081587	0.084371	1.245329
21	6	0	-3.080594	1.037434	-0.693508
22	1	0	-3.079140	-1.259094	-1.631256
23	1	0	-3.080003	-2.060761	-0.000411
24	1	0	-3.089462	1.031194	1.787130
25	1	0	-3.085824	-0.782007	1.908308
26	1	0	-3.088676	2.044729	-0.274711
27	1	0	-3.083761	1.033259	-1.784406
28	6	0	-3.982702	-0.000794	-0.000588
29	17	0	-5.760986	-0.004736	-0.003312

Using Default Heptane Solvent Model

\# opt freq uwb97xd/6-311+g(d,p) scrf=(solvent=heptane) geom=connectivity
Charge = 0 Multiplicity = 1

Center Number	Atomic Number	Atomic Type	Coordinates (Angstroms) X	Y	Z
1	6	0	0.263607	1.124129	0.572015
2	1	0	0.269878	1.250046	1.654236
3	1	0	0.271687	2.070784	0.032835
4	6	0	0.269422	-0.060188	-1.242031
5	1	0	0.283981	-1.000647	-1.791576
6	1	0	0.276803	0.814456	-1.891087
7	6	0	0.268365	-1.039455	0.690111
8	1	0	0.282751	-2.039152	0.257020
9	1	0	0.274468	-1.045627	1.779077
10	6	0	-0.666035	0.005200	0.004615
11	6	0	1.159310	0.011461	0.009424
12	16	0	2.984905	0.000924	0.000689
13	9	0	3.048954	0.814000	-1.414733
14	9	0	3.010369	-1.419680	-0.808486
15	9	0	4.602699	-0.036325	-0.030151
16	9	0	3.064934	-0.803541	1.419626
17	9	0	3.101160	1.414539	0.807872
18	6	0	-2.145883	0.002268	0.002278
19	6	0	-3.077225	-1.111023	-0.562687
20	6	0	-3.081881	0.068337	1.245421
21	6	0	-3.080442	1.044688	-0.680038
22	1	0	-3.081618	-1.239568	-1.646213
23	1	0	-3.082960	-2.060731	-0.025728
24	1	0	-3.090687	1.008124	1.799617
25	1	0	-3.087780	-0.805880	1.898345
26	1	0	-3.088973	2.047373	-0.249754
27	1	0	-3.085186	1.054425	-1.770987
28	6	0	-3.985190	-0.000661	-0.000549
29	17	0	-5.758716	-0.003404	-0.003253

SC-XRD Data

Structure search in the Cambridge Structural Database (CSD)

A database (CSD Version 5.42, November 2021) survey was performed to search for related structural motives. The following search motive was used in the ConQuest (Version 2020.1) program:

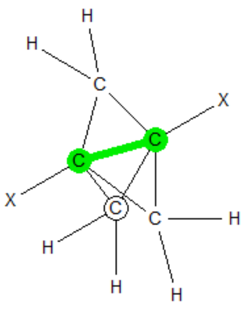

The initial search found 153 structures containing the predefined entity.

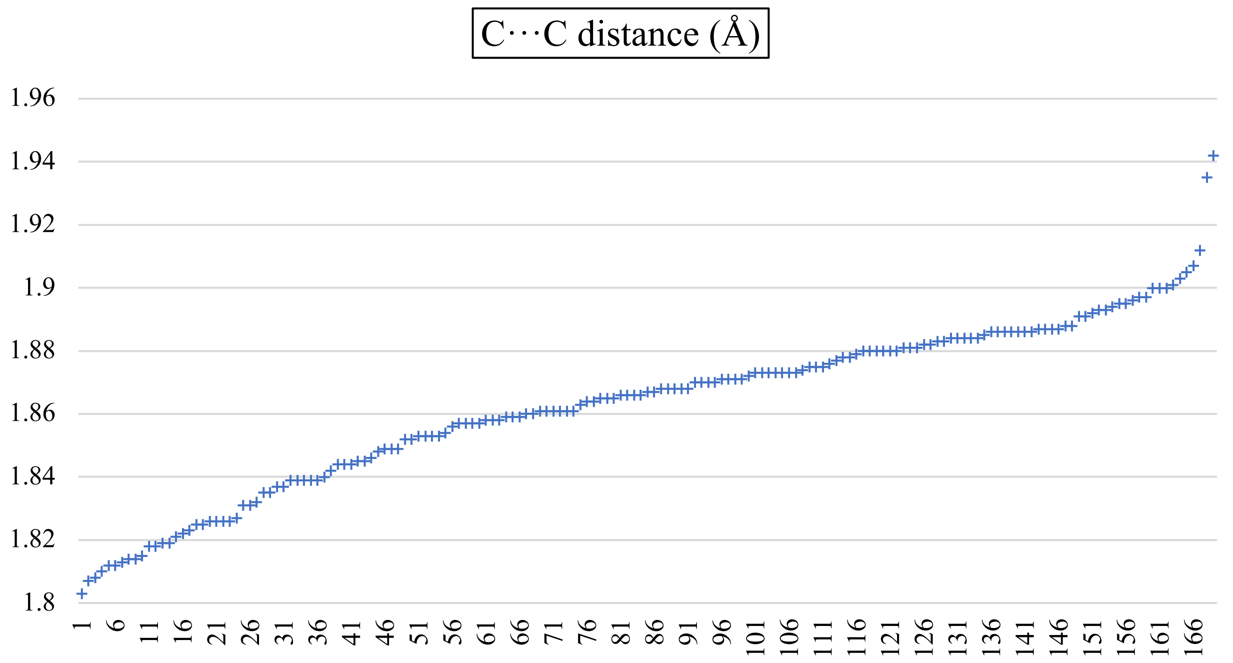

Figure S46. Structures containing bicyclo[1.1.1]pentane in the CSD showing the variation in the $C_1 \cdots C_3$ distance (sorted by distances; ordinate: distance in Å; abscissa: number of independent distances in the CSD)

The chosen dataset gives an average C···C distance of 1.86(3) Å for the initially defined motive. The strucutre Ref. codes VUFXUP (1.803 Å) and IKUBEY (1.942 Å) where found to have the shortest and longest known C···C distances at the time of the survey.

Single crystal structure analysis of compound 2

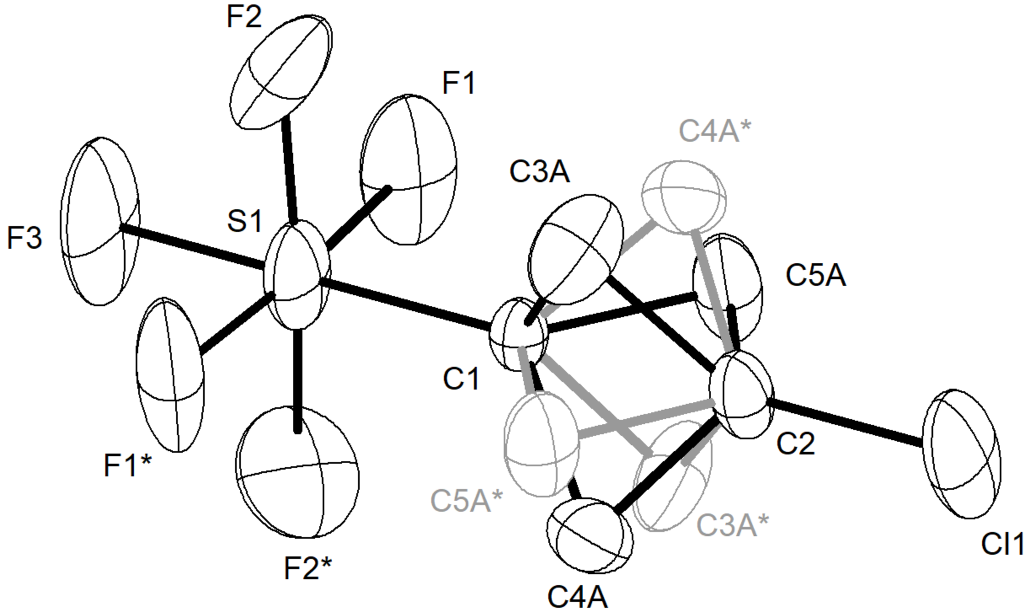

Figure S47. The molecular structure of compound **2**. H atoms have been removed for clarity.

X-ray Crystal Structure Analysis of compound 2:

C_5 H_6 Cl F_5 S, M_r = 228.61 g mol^{-1}, colorless prism, crystal size 0.051 x 0.042 x 0.032 mm^3, tetragonal, space group $P4_12_12$ [92], a = 6.9051(3) Å, c = 17.3184(12) Å, V = 825.75(9) Å^3, T = 100(2) K, Z = 4, D_{calc} = 1.839 g·cm^3, λ = 0.71073 Å, $\mu(Mo\text{-}K_{\alpha})$ = 0.741 mm^{-1}, Gaussian absorption correction ($T_{\min}$ = 0.97414, $T_{\max}$ = 0.98582), Bruker-AXS Kappa Mach3 with APEX-II detector and IµS microfocus Mo-anode X-ray source, 3.176 < θ < 28.354°, 18060 measured reflections, 1031 independent reflections, 777 reflections with $I > 2\sigma(I)$, R_{int} = 0.0623. The structure was solved by *SHELXT* and refined by full-matrix least-squares (*SHELXL*) against F^2 to R_1 = 0.0446 [$I > 2\sigma(I)$], wR_2 = 0.1075 [all data], 72 parameters.

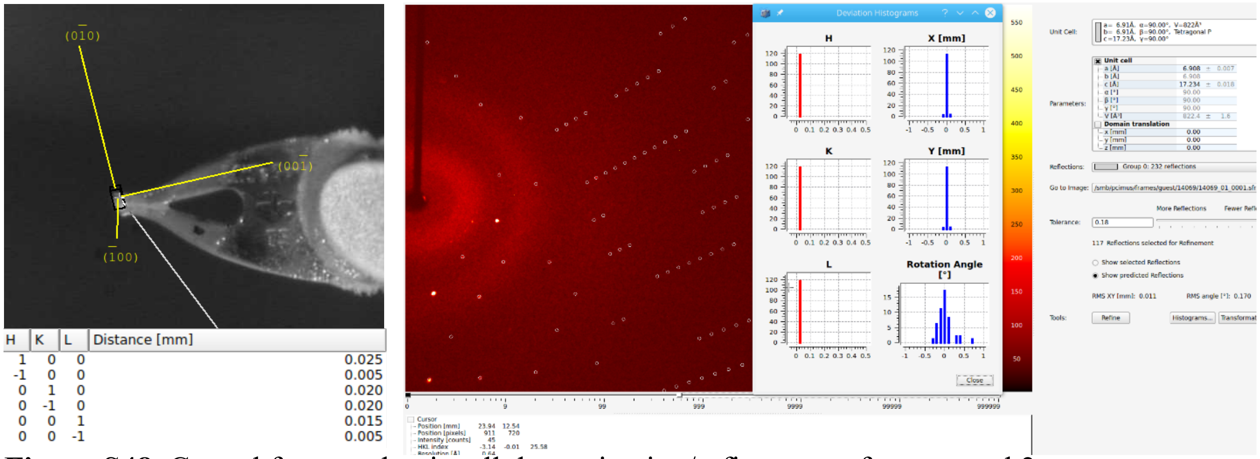

Figure S48. Crystal faces and unit cell determination/refinement of compound **2**.

INTENSITY STATISTICS FOR DATASET

Resolution	#Data	#Theory	%Complete	Redundancy	Mean I	Mean I/s	Rmerge	Rsigma
Inf - 3.66	11	13	84.6	20.31	159.22	129.25	0.0224	0.0100
3.66 - 2.32	23	23	100.0	32.17	33.38	140.69	0.0228	0.0057
2.32 - 1.77	34	35	97.1	40.09	14.17	104.00	0.0354	0.0080
1.77 - 1.53	33	35	94.3	39.94	13.85	89.94	0.0406	0.0082
1.53 - 1.35	37	38	97.4	43.84	12.12	89.47	0.0444	0.0087
1.35 - 1.24	35	35	100.0	45.06	8.51	71.28	0.0584	0.0122
1.24 - 1.16	32	32	100.0	41.69	6.01	49.08	0.0816	0.0175
1.16 - 1.10	32	32	100.0	39.31	4.81	41.18	0.1034	0.0213
1.10 - 1.05	34	34	100.0	34.74	5.95	31.18	0.1301	0.0237
1.05 - 1.00	33	33	100.0	27.55	1.97	18.24	0.1667	0.0536
1.00 - 0.96	39	39	100.0	21.44	1.69	14.18	0.2189	0.0723
0.96 - 0.93	34	34	100.0	21.09	1.51	11.03	0.2765	0.0802
0.93 - 0.90	33	33	100.0	19.58	1.35	10.04	0.2568	0.0946
0.90 - 0.87	39	39	100.0	17.10	1.76	8.99	0.2024	0.0788
0.87 - 0.85	35	35	100.0	16.23	1.51	8.40	0.2870	0.1028
0.85 - 0.83	30	30	100.0	17.13	0.94	6.15	0.3696	0.1480
0.83 - 0.81	34	34	100.0	15.50	0.59	3.96	0.5317	0.2625
0.81 - 0.79	38	38	100.0	15.89	0.96	5.78	0.3867	0.1648
0.79 - 0.78	24	24	100.0	15.21	0.74	4.54	0.4197	0.2207
0.78 - 0.76	44	44	100.0	14.41	0.59	3.50	0.5156	0.2841
0.76 - 0.75	29	30	96.7	12.03	0.59	2.71	0.5376	0.3587
0.85 - 0.75	199	200	99.5	15.02	0.73	4.42	0.4450	0.2259
Inf - 0.75	683	690	99.0	26.33	7.68	35.58	0.0589	0.0207

Complete .cif-data of the compound are available under the CCDC number **CCDC-2195538**. The investigated crystal is twinned by inversion (TWIN -1 0 0 0 -1 0 0 0 -1) with a free refined BASF value of 0.5(3). Additionally, the molecule is located on a crystallographic special position (two fold rotational axis) and the bicyclo[1.1.1]pentane unit is rotationally disorderd over two positions. This group was refined with fixed occupancy of 50% for PART -1.

To take the molecular displacement into account a libration correction was later applied to the structure. Two different programs where used: XP (LIBR ALL) and PLATON (CALC TMP).[9a-e] This lead to the following, corrected atomic bond distances:

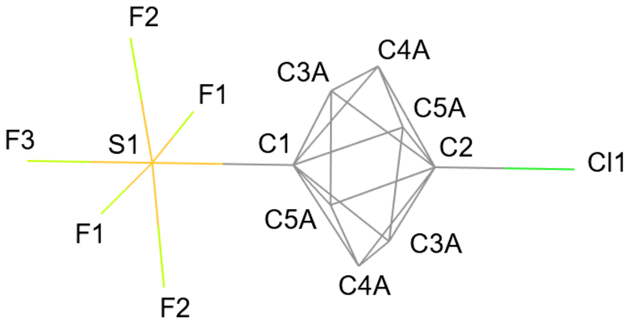


```
Librational tensors and esds (orthogonal axes)

L (radians**2)

 0.0060 (0.0011) -0.0024 (0.0007) -0.0017 (0.0007)
                  0.0060 (0.0011) -0.0018 (0.0007)
                                   0.0088 (0.0007)


S (radians.A)

 0.0001 (0.0015) -0.0006 (0.0010) -0.0012 (0.0010)
-0.0006 (0.0010)  0.0002 (0.0015) -0.0016 (0.0010)
-0.0012 (0.0009) -0.0016 (0.0009) -0.0003 (0.0015)


T (A**2)

 0.0303 (0.0031)  0.0079 (0.0027)  0.0016 (0.0028)
                  0.0291 (0.0031)  0.0011 (0.0028)
                                   0.0352 (0.0035)

RG = 0.2107

Librational corrections and corrected bond lengths

  0.009    1.583  F1    S1
  0.014    1.594  F2    S1
  0.010    1.589  F3    S1
  0.011    1.790  S1    C1
  0.011    1.770  CL1    C2
  0.011    1.800  C1    C2
  0.013    1.566  C1    C3A
  0.013    1.622  C2    C3A
  0.006    0.996  C3A    H3AA
  0.007    0.997  C3A    H3AB
  0.010    1.543  C1    C4A
  0.010    1.477  C2    C4A
  0.006    0.996  C4A    H4AA
  0.009    0.999  C4A    H4AB
  0.009    1.551  C1    C5A
  0.009    1.511  C2    C5A
  0.009    0.999  C5A    H5AA
  0.007    0.997  C5A    H5AB
  0.009    1.583  S1    F1A
  0.014    1.594  S1    F2A
```

Rigid-Body Model Libration Corrections for Bond Distances and "Hirshfeld Rigid-Bond" Test (Acta Cryst., 1976, A32, 239-244)

Bond		Bond Distance		Components of the Correction			MSDA from U(obs) Vibration Along the Interatomic Bond				Angle with Lib. Axes		
Atom(I)	Atom(J)	Obsd	Calcd	Del(L)	Del(M)	Del(N)	I to J	J to I	Difference	Sqrt(Diff)	L(1)	L(2)	L(3)
Cl1	- C2	1.759(4)	1.7719	-0.0126	0	0	0.0175(10)	0.019(2)	0.002(2)	0.0447	180.00	90.01	89.99
S1	- F1	1.574(3)	1.5983	-0.0005	-0.0084	-0.0234	0.0400(7)	0.039(2)	0.001(2)	0.0316	92.29	162.77	107.01
S1	- F2	1.580(3)	1.6104	-0.0005	-0.0268	0.0135	0.0726(7)	0.070(2)	0.003(2)	0.0548	92.29	107.54	17.65
S1	- F3	1.579(3)	1.5903	-0.0113	0	0	0.0154(7)	0.013(2)	0.002(2)	0.0447	180.00	90.01	89.99
S1	- C1	1.779(3)	1.7920	0.0127	0	0	0.0154(7)	0.0168(16)	0.0014(17)	0.0374	0.00	89.99	90.01
C1	- C3A	1.553(9)	1.5776	0.0060	0.0171	-0.0184	0.0237(16)	0.024(5)	0.000(5)	0	57.06	96.56	146.26
C1	- C4A	1.532(9)	1.5526	0.0068	-0.0203	-0.0018	0.0228(16)	0.028(5)	0.005(5)	0.0707	51.70	128.48	61.39
C1	- C5A	1.541(10)	1.5606	0.0066	0.0063	0.0184	0.0186(16)	0.025(5)	0.006(5)	0.0775	52.97	40.49	75.94
C2	- C3A	1.610(10)	1.6338	-0.0068	0.0171	-0.0184	0.044(2)	0.035(5)	0.009(5)	0.0949	125.92	96.33	143.31
C2	- C4A	1.467(8)	1.4873	-0.0060	-0.0203	-0.0018	0.030(2)	0.026(5)	0.004(5)	0.0632	124.91	130.54	59.98
C2	- C5A	1.502(10)	1.5210	-0.0062	0.0063	0.0184	0.023(2)	0.030(5)	0.007(5)	0.0837	124.97	38.73	75.55

Sqrt(Sum(DelIJ**2)/Nrb) = 0.0052

# - Indicates bonds exceeding the 5.0 sigma test level

Figure S49. Libration corrections for compound **2**. Wire plot of compound 2 (top left), XP output (top right) and PLATON output (bottom).

Table S5. Crystal data and structure refinement of compound **2**

Identification code	14069	
Empirical formula	$C_5 H_6 Cl F_5 S$	
Color	colourless	
Formula weight	228.61 g·mol^{-1}	
Temperature	100(2) K	
Wavelength	0.71073 Å	
Crystal system	Tetragonal	
Space group	$P4_12_12$, (no. 92)	
Unit cell dimensions	a = 6.9051(3) Å	α= 90°.
	b = 6.9051(3) Å	β= 90°.
	c = 17.3184(12) Å	γ = 90°.
Volume	825.75(9) Å^3	
Z	4	
Density (calculated)	1.839 Mg·m^{-3}	
Absorption coefficient	0.741 mm^{-1}	
F(000)	456 e	
Crystal size	0.051 x 0.042 x 0.032 mm^3	
θ range for data collection	3.176 to 28.354°.	
Index ranges	-9 ≤ h ≤ 9, -9≤ k ≤ 9, -23 ≤ l ≤ 23	
Reflections collected	18060	
Independent reflections	1031 [R_{int} = 0.0623]	
Reflections with I>2σ(I)	777	
Completeness to θ = 25.242°	99.8 %	
Absorption correction	Gaussian	
Max. and min. transmission	0.98582 and 0.97414	
Refinement method	Full-matrix least-squares on F^2	
Data / restraints / parameters	1031 / 0 / 72	
Goodness-of-fit on F^2	1.071	
Final R indices [I>2σ(I)]	R_1 = 0.0446	wR^2 = 0.0944
R indices (all data)	R_1 = 0.0733	wR^2 = 0.1075
Absolute structure parameter	0.5(3)	
Extinction coefficient	n/a	
Largest diff. peak and hole	0.477 and -0.283 e·Å^{-3}	

Table S6. Bond lengths [Å] and angles [°] of compound **2**

Cl(1)-C(2)	1.759(5)	F(1)-S(1)	1.574(3)
F(2)-S(1)	1.580(3)	F(3)-S(1)	1.579(4)
S(1)-C(1)	1.779(4)	C(1)-C(2)	1.789(6)
C(1)-C(3A)	1.553(9)	C(1)-C(4A)	1.533(8)
C(1)-C(5A)	1.542(9)	C(2)-C(3A)	1.609(10)
C(2)-C(4A)	1.468(8)	C(2)-C(5A)	1.502(9)
C(3A)-H(3AA)	0.9900	C(3A)-H(3AB)	0.9900
C(4A)-H(4AA)	0.9900	C(4A)-H(4AB)	0.9900
C(5A)-H(5AA)	0.9900	C(5A)-H(5AB)	0.9900
F(1)-S(1)-F(1)#1	175.4(2)	F(1)#1-S(1)-F(2)	90.4(2)
F(1)#1-S(1)-F(2)#1	89.4(2)	F(1)-S(1)-F(2)	89.4(2)
F(1)-S(1)-F(2)#1	90.4(2)	F(1)-S(1)-F(3)	87.70(12)
F(1)#1-S(1)-F(3)	87.70(12)	F(1)-S(1)-C(1)	92.30(12)
F(1)#1-S(1)-C(1)	92.30(12)	F(2)-S(1)-F(2)#1	175.4(3)
F(2)-S(1)-C(1)	92.32(13)	F(2)#1-S(1)-C(1)	92.32(13)
F(3)-S(1)-F(2)	87.68(13)	F(3)-S(1)-F(2)#1	87.68(13)
F(3)-S(1)-C(1)	180.00(9)	S(1)-C(1)-C(2)	180.0
C(3A)-C(1)-S(1)	123.0(4)	C(3A)-C(1)-C(2)	57.0(4)
C(4A)-C(1)-S(1)	128.3(3)	C(4A)-C(1)-C(2)	51.7(3)
C(4A)-C(1)-C(3A)	89.5(5)	C(4A)-C(1)-C(5A)	89.1(5)
C(5A)-C(1)-S(1)	127.0(4)	C(5A)-C(1)-C(2)	53.0(4)
C(5A)-C(1)-C(3A)	87.8(6)	Cl(1)-C(2)-C(1)	180.0(3)
C(3A)-C(2)-Cl(1)	125.9(3)	C(3A)-C(2)-C(1)	54.1(3)
C(4A)-C(2)-Cl(1)	124.9(4)	C(4A)-C(2)-C(1)	55.1(4)
C(4A)-C(2)-C(3A)	89.7(5)	C(4A)-C(2)-C(5A)	93.1(5)
C(5A)-C(2)-Cl(1)	125.0(4)	C(5A)-C(2)-C(1)	55.0(4)
C(5A)-C(2)-C(3A)	87.1(6)	C(1)-C(3A)-C(2)	68.9(4)
C(1)-C(3A)-H(3AA)	116.8	C(1)-C(3A)-H(3AB)	116.8
C(2)-C(3A)-H(3AA)	116.8	C(2)-C(3A)-H(3AB)	116.8
H(3AA)-C(3A)-H(3AB)	113.8	C(1)-C(4A)-H(4AA)	116.2
C(1)-C(4A)-H(4AB)	116.2	C(2)-C(4A)-C(1)	73.2(4)
C(2)-C(4A)-H(4AA)	116.2	C(2)-C(4A)-H(4AB)	116.2

H(4AA)-C(4A)-H(4AB)	113.2	C(1)-C(5A)-H(5AA)	116.4
C(1)-C(5A)-H(5AB)	116.4	C(2)-C(5A)-C(1)	72.0(4)
C(2)-C(5A)-H(5AA)	116.4	C(2)-C(5A)-H(5AB)	116.4
H(5AA)-C(5A)-H(5AB)	113.4		

Symmetry transformations used to generate equivalent atoms:

#1 -y+1,-x+1,-z+3/2

Single crystal structure analysis of compound 3

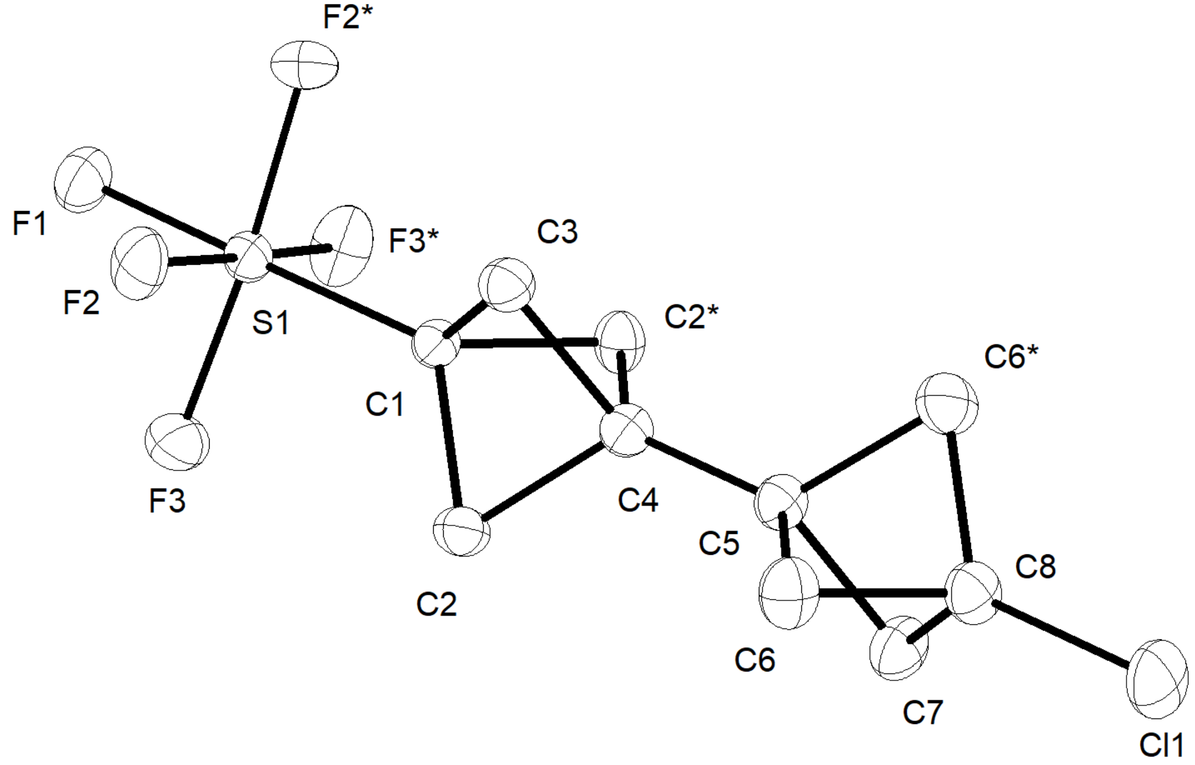

Figure S50. The molecular structure of compound **3**. H atoms have been removed for clarity.

X-ray Crystal Structure Analysis of compound 3:

C_{10} H_{12} Cl F_5 S, M_r = 294.71 g mol^{-1}, colorless block, crystal size 0.071 x 0.061 x 0.058 mm^3, orthorhombic, space group *Pnma* [62], a = 18.5597(10) Å, b = 8.9377(5) Å, c = 7.1509(4) Å, V = 1186.20(11) Å^3, T = 100(2) K, Z = 4, D_{calc} = 1.650 g·cm^3, λ = 0.71073 Å, $\mu(Mo\text{-}K_\alpha)$ = 0.536 mm^{-1}, Gaussian absorption correction ($T_{\min}$ = 0.96961, $T_{\max}$ = 0.98448), Bruker-AXS Kappa Mach3 with APEX-II detector and IµS microfocus Mo-anode X-ray source, 2.195 < θ < 30.997°, 34854 measured reflections, 2003 independent reflections, 1438 reflections with $I > 2\sigma(I)$, R_{int} = 0.0886. The structure was solved by *SHELXT* and refined by full-matrix least-squares (*SHELXL*) against F^2 to R_1 = 0.0446 [$I > 2\sigma(I)$], wR_2 = 0.1181 [all data], 91 parameters.

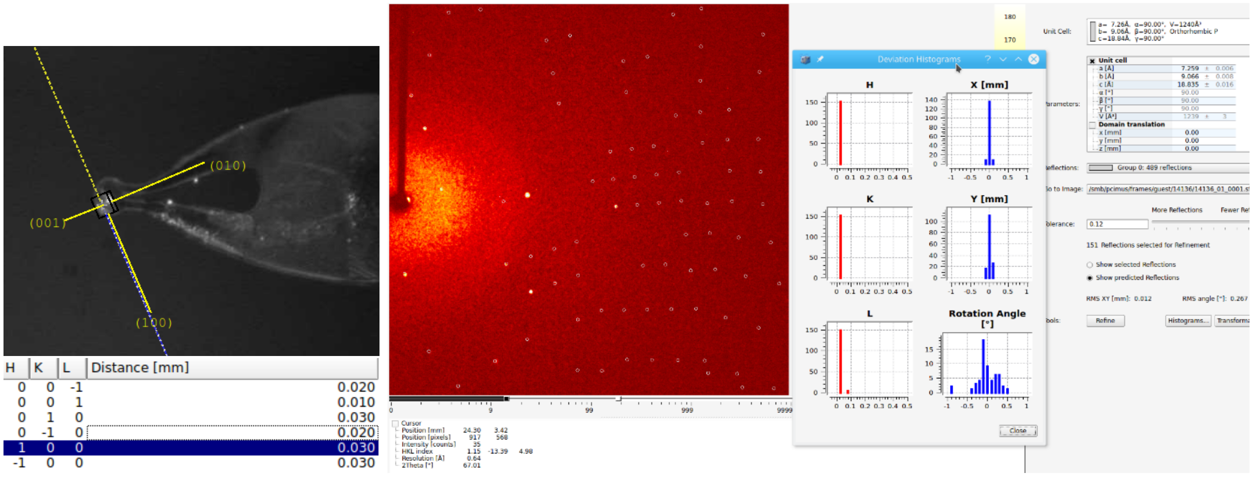

Figure S51. Crystal faces and unit cell determination/refinement of compound **3**.

INTENSITY STATISTICS FOR DATASET

Resolution	#Data	#Theory	%Complete	Redundancy	Mean I	Mean I/s	Rmerge	Rsigma
Inf - 3.23	35	35	100.0	19.43	51.92	47.80	0.0349	0.0115
3.23 - 2.01	82	82	100.0	26.22	16.59	60.47	0.0390	0.0112
2.01 - 1.54	118	118	100.0	28.28	11.31	50.51	0.0458	0.0125
1.54 - 1.33	118	118	100.0	29.34	10.85	52.52	0.0550	0.0138
1.33 - 1.20	110	110	100.0	29.25	7.87	38.44	0.0658	0.0163
1.20 - 1.10	130	130	100.0	27.93	6.13	27.10	0.0927	0.0222
1.10 - 1.03	114	114	100.0	22.04	3.57	21.79	0.1249	0.0363
1.03 - 0.98	110	110	100.0	17.41	3.64	17.63	0.1303	0.0421
0.98 - 0.93	126	126	100.0	15.72	2.34	12.13	0.1847	0.0642
0.93 - 0.89	118	118	100.0	13.79	2.15	8.98	0.2183	0.0783
0.89 - 0.86	119	119	100.0	12.77	1.91	8.50	0.2433	0.0939
0.86 - 0.83	122	122	100.0	12.31	2.08	8.57	0.2308	0.0931
0.83 - 0.81	84	84	100.0	11.99	1.08	5.64	0.3254	0.1646
0.81 - 0.78	168	168	100.0	11.69	1.36	6.22	0.3164	0.1445
0.78 - 0.76	117	117	100.0	10.79	1.08	4.96	0.3740	0.1925
0.76 - 0.75	68	68	100.0	11.22	1.03	4.78	0.3836	0.1976
0.75 - 0.73	139	138	100.7	10.79	1.04	4.19	0.4077	0.2115
0.73 - 0.71	146	147	99.3	10.48	1.06	4.08	0.4359	0.2183
0.71 - 0.70	94	94	100.0	9.73	1.04	4.06	0.3809	0.2373
0.70 - 0.69	85	85	100.0	9.78	0.77	2.94	0.5046	0.3211
0.69 - 0.67	115	149	77.2	5.85	0.67	2.25	0.5557	0.4659
0.77 - 0.67	707	741	95.4	9.48	0.95	3.78	0.4279	0.2550
Inf - 0.67	2318	2352	98.6	16.23	4.46	17.30	0.0859	0.0433

Complete .cif-data of the compound are available under the CCDC number **CCDC-2195540**.

To take the molecular displacement into account a libration correction was later applied to the structure. Two different programs where used: XP (LIBR ALL) and PLATON (CALC TMP).[9a-e] This leads to the following, corrected atomic bond distances:

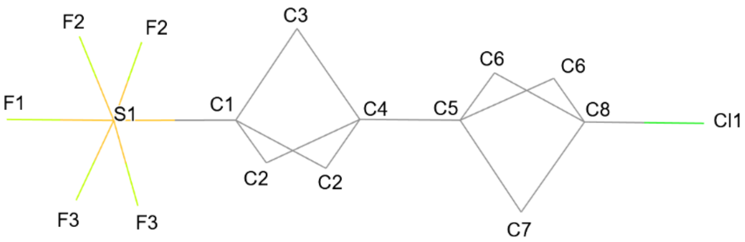


```
Librational tensors and esds (orthogonal axes)

L (radians**2)

 0.0016 (0.0002)  0.0000 (0.0001)  0.0008 (0.0002)
                  0.0008 (0.0001)  0.0000 (0.0001)
                                   0.0010 (0.0002)


S (radians.A)

 0.0000 (0.0004)  0.0003 (0.0002)  0.0000 (0.0002)
 0.0003 (0.0001)  0.0000 (0.0004)  0.0005 (0.0001)
 0.0000 (0.0002)  0.0005 (0.0002)  0.0000 (0.0004)


T (A**2)

 0.0203 (0.0007)  0.0000 (0.0007)  0.0012 (0.0007)
                  0.0193 (0.0009)  0.0000 (0.0007)
                                   0.0202 (0.0007)

RG = 0.1649

Librational corrections and corrected bond lengths

   0.002   1.588  S1   F2
   0.001   1.584  S1   F1
   0.002   1.599  S1   F3
   0.001   1.806  S1   C1
   0.002   1.541  C1   C3
   0.001   0.991  C3   H3A
   0.001   0.991  C3   H3B
   0.001   1.766  CL1   C8
   0.001   1.840  C8   C5
   0.001   1.837  C1   C4
   0.002   1.566  C3   C4
   0.001   1.484  C5   C4
   0.002   1.542  C1   C2
   0.002   1.565  C4   C2
   0.001   0.991  C2   H2A
   0.001   0.991  C2   H2B
   0.002   1.543  C8   C6
   0.002   1.555  C5   C6
   0.001   0.991  C6   H6A
   0.001   0.991  C6   H6B
   0.002   1.540  C8   C7
   0.002   1.561  C5   C7
   0.001   0.991  C7   H7A
   0.001   0.991  C7   H7B
   0.002   1.588  S1   F2A
   0.002   1.599  S1   F3A
   0.002   1.542  C1   C2A
   0.002   1.565  C4   C2A
   0.001   0.991  C2A   H2AA
   0.001   0.991  C2A   H2BA
   0.002   1.543  C8   C6A
   0.002   1.555  C5   C6A
   0.001   0.991  C6A   H6AA
   0.001   0.991  C6A   H6BA
```

```
=================================================================================================================
Rigid-Body Model Libration Corrections for Bond Distances and "Hirshfeld Rigid-Bond" Test (Acta Cryst., 1976, A32, 239-244)
=================================================================================================================

                                                                       MSDA from U(obs)
     Bond           Bond Distance   Components of the Correction  Vibration Along the Interatomic Bond        Angle with Lib. Axes

Atom(I)  Atom(J)         Obsd       Calcd  Del(L)  Del(M)  Del(N)     I to J      J to I    Difference  Sqrt(Diff)    L(1)    L(2)    L(3)
-----------------------------------------------------------------------------------------------------------------
Cl1     - C2         1.759(4)    1.7719 -0.0126       0       0  0.0175(10)    0.019(2)     0.002(2)     0.0447   180.00   90.01   89.99
S1      - F1         1.574(3)    1.5983 -0.0005 -0.0084 -0.0234   0.0400(7)    0.039(2)     0.001(2)     0.0316    92.29  162.77  107.01
S1      - F2         1.580(3)    1.6104 -0.0005 -0.0268  0.0135   0.0726(7)    0.070(2)     0.003(2)     0.0548    92.29  107.54   17.65
S1      - F3         1.579(3)    1.5903 -0.0113       0       0   0.0154(7)    0.013(2)     0.002(2)     0.0447   180.00   90.01   89.99
S1      - C1         1.779(3)    1.7920  0.0127       0       0   0.0154(7)  0.0168(16)   0.0014(17)     0.0374     0.00   89.99   90.01
C1      - C3A        1.553(9)    1.5776  0.0060  0.0171 -0.0184  0.0237(16)    0.024(5)     0.000(5)          0    57.06   96.56  146.26
C1      - C4A        1.532(9)    1.5526  0.0068 -0.0203 -0.0018  0.0228(16)    0.028(5)     0.005(5)     0.0707    51.70  128.48   61.39
C1      - C5A       1.541(10)    1.5606  0.0066  0.0063  0.0184  0.0186(16)    0.025(5)     0.006(5)     0.0775    52.97   40.49   75.94
C2      - C3A       1.610(10)    1.6338 -0.0068  0.0171 -0.0184    0.044(2)    0.035(5)     0.009(5)     0.0949   125.92   96.33  143.31
C2      - C4A        1.467(8)    1.4873 -0.0060 -0.0203 -0.0018    0.030(2)    0.026(5)     0.004(5)     0.0632   124.91  130.54   59.98
C2      - C5A       1.502(10)    1.5210 -0.0062  0.0063  0.0184    0.023(2)    0.030(5)     0.007(5)     0.0837   124.97   38.73   75.55

                                                       Sqrt(Sum(DelIJ**2)/Nrb) =     0.0052

                                  # - Indicates bonds exceeding the 5.0 sigma test level
```

Figure S52. Libration corrections for compound **3**. Wire plot of compound **3** (top left), XP output (top right) and PLATON output (bottom).

Table S7. Crystal data and structure refinement of compound **3**

Identification code	14136	
Empirical formula	$C_{10} H_{12} Cl F_5 S$	
Color	colourless	
Formula weight	294.71 g·mol^{-1}	
Temperature	100(2) K	
Wavelength	0.71073 Å	
Crystal system	Orthorhombic	
Space group	*Pnma*, (no. 62)	
Unit cell dimensions	a = 18.5597(10) Å	α= 90°.
	b = 8.9377(5) Å	β= 90°.
	c = 7.1509(4) Å	γ = 90°.
Volume	1186.20(11) Å^3	
Z	4	
Density (calculated)	1.650 Mg·m^{-3}	
Absorption coefficient	0.536 mm^{-1}	
F(000)	600 e	
Crystal size	0.071 x 0.061 x 0.058 mm^3	
θ range for data collection	2.195 to 30.997°.	
Index ranges	$-26 \leq h \leq 26$, $-12 \leq k \leq 12$, $-10 \leq l \leq 10$	
Reflections collected	34854	
Independent reflections	2003 [R_{int} = 0.0886]	
Reflections with I>2σ(I)	1438	
Completeness to θ = 25.242°	100.0 %	
Absorption correction	Gaussian	
Max. and min. transmission	0.98448 and 0.96961	
Refinement method	Full-matrix least-squares on F^2	
Data / restraints / parameters	2003 / 0 / 91	
Goodness-of-fit on F^2	1.064	
Final R indices [I>2σ(I)]	R_1 = 0.0446	wR^2 = 0.1040
R indices (all data)	R_1 = 0.0758	wR^2 = 0.1181
Extinction coefficient	n/a	
Largest diff. peak and hole	0.723 and -0.401 e·Å^{-3}	

Table S8. Bond lengths [Å] and angles [°] of compound **3**

S(1)-F(2)	1.5860(13)	S(1)-F(2)#1	1.5860(13)
S(1)-F(1)	1.5831(19)	S(1)-F(3)	1.5967(13)
S(1)-F(3)#1	1.5967(13)	S(1)-C(1)	1.805(3)
Cl(1)-C(8)	1.765(3)	C(1)-C(3)	1.539(4)
C(1)-C(4)	1.835(4)	C(1)-C(2)#1	1.540(3)
C(1)-C(2)	1.540(3)	C(3)-H(3A)	0.9900
C(3)-H(3B)	0.9900	C(3)-C(4)	1.564(4)
C(8)-C(5)	1.839(4)	C(8)-C(6)#1	1.542(3)
C(8)-C(6)	1.542(3)	C(8)-C(7)	1.538(4)
C(5)-C(4)	1.483(4)	C(5)-C(6)#1	1.554(3)
C(5)-C(6)	1.554(3)	C(5)-C(7)	1.560(4)
C(4)-C(2)	1.563(3)	C(4)-C(2)#1	1.563(3)
C(2)-H(2A)	0.9900	C(2)-H(2B)	0.9900
C(6)-H(6A)	0.9900	C(6)-H(6B)	0.9900
C(7)-H(7A)	0.9900	C(7)-H(7B)	0.9900
F(2)#1-S(1)-F(2)	90.80(10)	F(2)#1-S(1)-F(3)#1	174.66(8)
F(2)-S(1)-F(3)#1	89.77(7)	F(2)#1-S(1)-F(3)	89.77(7)
F(2)-S(1)-F(3)	174.66(8)	F(2)-S(1)-C(1)	92.21(8)
F(2)#1-S(1)-C(1)	92.21(8)	F(1)-S(1)-F(2)	87.42(7)
F(1)-S(1)-F(2)#1	87.42(7)	F(1)-S(1)-F(3)#1	87.31(7)
F(1)-S(1)-F(3)	87.31(7)	F(1)-S(1)-C(1)	179.47(12)
F(3)-S(1)-F(3)#1	89.17(11)	F(3)#1-S(1)-C(1)	93.07(8)
F(3)-S(1)-C(1)	93.07(8)	S(1)-C(1)-C(4)	179.78(19)
C(3)-C(1)-S(1)	125.40(19)	C(3)-C(1)-C(4)	54.38(16)
C(3)-C(1)-C(2)#1	89.50(17)	C(3)-C(1)-C(2)	89.50(17)
C(2)-C(1)-S(1)	125.80(14)	C(2)#1-C(1)-S(1)	125.80(14)
C(2)#1-C(1)-C(4)	54.31(12)	C(2)-C(1)-C(4)	54.31(12)
C(2)-C(1)-C(2)#1	89.3(2)	C(1)-C(3)-H(3A)	116.3
C(1)-C(3)-H(3B)	116.3	C(1)-C(3)-C(4)	72.51(19)
H(3A)-C(3)-H(3B)	113.3	C(4)-C(3)-H(3A)	116.3
C(4)-C(3)-H(3B)	116.3	Cl(1)-C(8)-C(5)	179.8(2)
C(6)-C(8)-Cl(1)	126.05(15)	C(6)#1-C(8)-Cl(1)	126.05(15)

C(6)-C(8)-C(5)	53.85(13)	C(6)#1-C(8)-C(5)	53.85(13)
C(6)-C(8)-C(6)#1	88.7(2)	C(7)-C(8)-Cl(1)	126.1(2)
C(7)-C(8)-C(5)	54.12(17)	C(7)-C(8)-C(6)#1	88.96(18)
C(7)-C(8)-C(6)	88.96(18)	C(4)-C(5)-C(8)	179.6(2)
C(4)-C(5)-C(6)	126.93(15)	C(4)-C(5)-C(6)#1	126.93(15)
C(4)-C(5)-C(7)	126.6(2)	C(6)#1-C(5)-C(8)	53.26(12)
C(6)-C(5)-C(8)	53.26(12)	C(6)#1-C(5)-C(6)	87.8(2)
C(6)-C(5)-C(7)	87.77(17)	C(6)#1-C(5)-C(7)	87.77(17)
C(7)-C(5)-C(8)	53.05(17)	C(3)-C(4)-C(1)	53.12(16)
C(5)-C(4)-C(1)	179.3(2)	C(5)-C(4)-C(3)	127.6(2)
C(5)-C(4)-C(2)#1	126.46(14)	C(5)-C(4)-C(2)	126.46(14)
C(2)-C(4)-C(1)	53.18(12)	C(2)#1-C(4)-C(1)	53.18(12)
C(2)-C(4)-C(3)	87.79(16)	C(2)#1-C(4)-C(3)	87.79(16)
C(2)-C(4)-C(2)#1	87.7(2)	C(1)-C(2)-C(4)	72.52(15)
C(1)-C(2)-H(2A)	116.3	C(1)-C(2)-H(2B)	116.3
C(4)-C(2)-H(2A)	116.3	C(4)-C(2)-H(2B)	116.3
H(2A)-C(2)-H(2B)	113.3	C(8)-C(6)-C(5)	72.89(16)
C(8)-C(6)-H(6A)	116.3	C(8)-C(6)-H(6B)	116.3
C(5)-C(6)-H(6A)	116.3	C(5)-C(6)-H(6B)	116.3
H(6A)-C(6)-H(6B)	113.3	C(8)-C(7)-C(5)	72.8(2)
C(8)-C(7)-H(7A)	116.3	C(8)-C(7)-H(7B)	116.3
C(5)-C(7)-H(7A)	116.3	C(5)-C(7)-H(7B)	116.3
H(7A)-C(7)-H(7B)	113.3		

Symmetry transformations used to generate equivalent atoms:

#1 x,-y+1/2,z

Differential scanning calorimetry and capillary crystallization of compound 4

Compound **4** is a liquid at ambient conditions and is not directly suitable for XRD experiments. Capillary crystallization of liquids is an established method for determining the crystal structure and absolute configuration of molecules by X-ray crystallography.[10a-f]

Compound **4** was initially investigated using differential scanning calorimetry (DSC) to determine the phase transition temperatures and the possible occurrence of polymorphism. DSC experiments where performed on a METTLER TOLEDO DSC 820 measuring module for thermal analysis. Two cycles with different cooling and heating rates where used for the sample (Figure S 5).

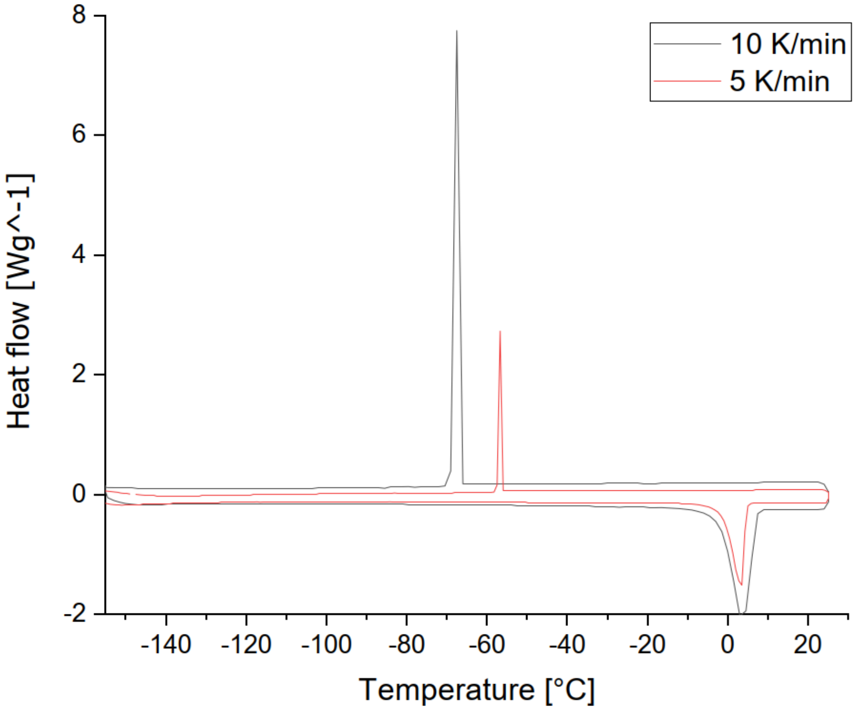

Figure S53. Ambient to low temperature DSC curve of **4** (SF5-Ph-Cl) using two different temperature gradients.

Crystals of **4** (SF5-Ph-Cl) were grown by first filling a 0.5 mm diameter WJM borosilicate glass capillary at ambient conditions. The capillary was then transferred to a diffractometer that was equipped with an Oxford Cryosystems' 700 Cryostream Cooler operating at 100 K. A crystalline powder was obtained directly. For recrystallizatoin the temperature was set slightly below the freezing point at 193 K. The samples were subsequently converted into high-quality single crystals by multiple melting and crystallization cycles. The crystal obtained in this fashion was used for the collection of diffraction data after cooling the capillary to 100 K.

Single crystal structure analysis of compound 4

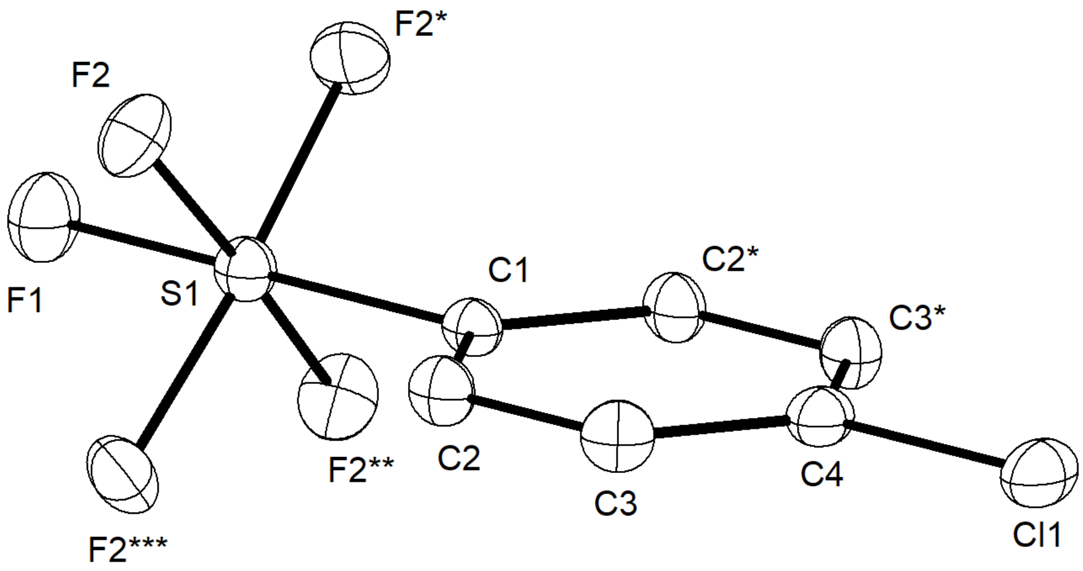

Figure S54. The molecular structure of compound **4**. H atoms have been removed for clarity.

X-ray Crystal Structure Analysis of compound 4:
$C_6 H_4 Cl F_5 S$, M_r = 238.609 g mol^{-1}, colorless capilliary, crystal size 0.432 x 0.409 x 0.40 mm^3, orthorhombic, space group *Cmcm* [63], a = 9.4508(11) Å, b = 12.6324(14) Å, c = 6.8646(8) Å, V = 819.54(16) Å^3, T = 100(2) K, Z = 4, D_{calc} = 1.934 g·cm^3, λ = 0.71073 Å, $\mu(Mo\text{-}K_\alpha)$ = 0.751 mm^{-1}, Gaussian absorption correction ($T_{\min}$ = 0.75517, $T_{\max}$ = 0.79046), Bruker-AXS Kappa Mach3 with APEX-II detector and IμS microfocus Mo-anode X-ray source, 2.69 < θ < 40.25°, 24861 measured reflections, 1421 independent reflections, 1333 reflections with $I > 2\sigma(I)$, R_{int} = 0.0288. The structure was solved by *SHELXT* and initially refined by full-matrix least-squares (*SHELXL*). The final structure refinement was performed by olex2.refine 1.5 (L-M) together with NoSpherA2 (atomic form factors) against F^2 to R_1 = 0.0135 [$I > 2\sigma(I)$], wR_2 = 0.0332 [all data] with 54 parameters.

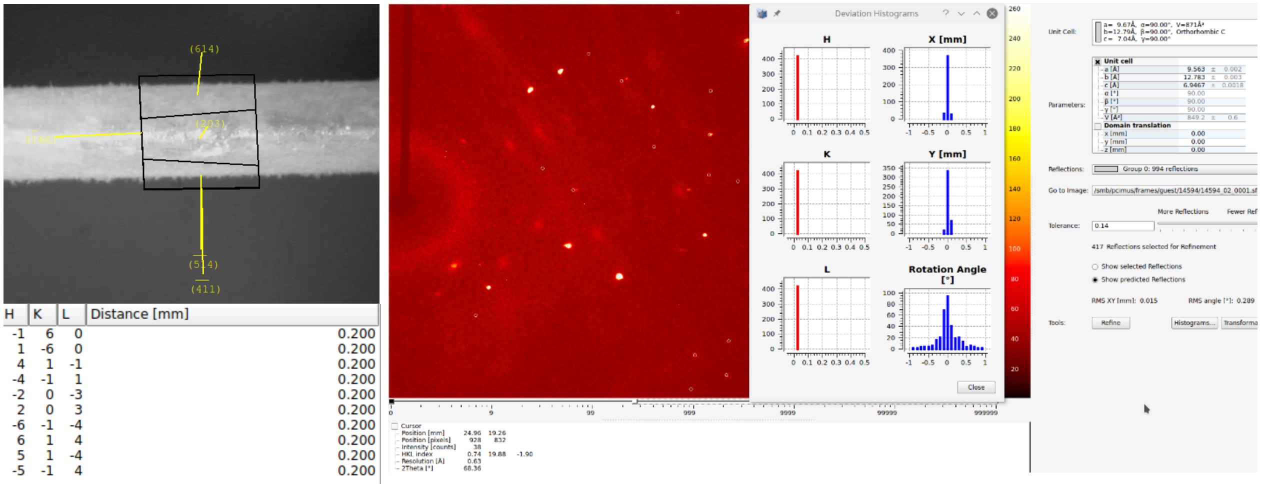

Figure S55. Crystal faces and unit cell determination/refinement of compound **4**.

```
INTENSITY STATISTICS FOR DATASET
```

Resolution	#Data	#Theory	%Complete	Redundancy	Mean I	Mean I/s	Rmerge	Rsigma
Inf - 2.32	24	28	85.7	20.57	105.77	117.22	0.0340	0.0083
2.32 - 1.54	55	55	100.0	32.38	123.50	131.62	0.0311	0.0097
1.54 - 1.20	81	82	98.8	35.65	55.79	137.84	0.0267	0.0070
1.20 - 1.04	76	76	100.0	31.01	55.68	111.93	0.0238	0.0080
1.04 - 0.94	79	79	100.0	24.71	20.21	99.40	0.0244	0.0086
0.94 - 0.87	79	79	100.0	21.05	18.85	79.34	0.0230	0.0098
0.87 - 0.81	90	90	100.0	19.61	15.70	78.45	0.0251	0.0106
0.81 - 0.77	77	77	100.0	18.61	11.97	71.47	0.0280	0.0108
0.77 - 0.73	93	93	100.0	17.42	8.27	60.85	0.0302	0.0111
0.73 - 0.70	80	80	100.0	15.98	9.46	61.66	0.0308	0.0116
0.70 - 0.68	67	67	100.0	15.76	6.54	52.74	0.0370	0.0136
0.68 - 0.66	68	68	100.0	14.60	5.76	47.63	0.0382	0.0153
0.66 - 0.64	87	87	100.0	14.79	4.72	46.18	0.0475	0.0143
0.64 - 0.62	84	84	100.0	13.39	3.67	40.66	0.0488	0.0180
0.62 - 0.60	109	109	100.0	13.39	2.92	34.38	0.0508	0.0183
0.60 - 0.59	53	53	100.0	7.49	3.13	24.54	0.0469	0.0292
0.59 - 0.58	57	57	100.0	5.00	2.13	18.85	0.0600	0.0347
0.58 - 0.56	134	135	99.3	4.53	2.19	19.15	0.0549	0.0372
0.56 - 0.55	72	72	100.0	4.24	1.64	15.66	0.0578	0.0451
0.55 - 0.54	117	123	95.1	3.59	1.18	12.21	0.0684	0.0581
0.64 - 0.54	626	633	98.9	7.30	2.34	23.42	0.0517	0.0305
Inf - 0.54	1582	1594	99.2	15.88	17.53	58.44	0.0288	0.0103

Complete .cif-data of the compound are available under the CCDC number **CCDC-2195541**.
The crystal was grown from the liquid in a long glass capillary. The maximum crystal size was estimated based on the diffraction geometry. For data acquisition, a fixed chi angle of 54.7 degrees was chosen to ensure that the exposed crystalline volume was as small as possible consistent with a high redundancy.
The molecule is located on a crystallographic special position (two orthogonal mirror planes). A

resolution cut off (SHEL 999 0.55) was applied to exclude poorly determined intensities at high diffraction angles.

The final structure refinement was carried out with using aspherical scattering factors with NoSpherA2. DFT-calculated with ORCA using a B3LYP functional and def2-TZVPP basis set, whereby the H atom positions were refined using anisotropy atomic displacement parameters. NoSpherA2 implementation of HAR makes use of tailor-made aspherical atomic form factors calculated on-the-fly from a Hirshfeld-partitioned electron density (ED) - not from spherical-atom form factors. The ED is calculated from a gaussian basis set single determinant SCF wavefunction - either Hartree-Fock or DFT using selected functionals - for a fragment of the crystal. This fragment can be embedded in an electrostatic crystal field by employing cluster charges or modelled using
implicit solvation models, depending on the software used. The following options were used:

SOFTWARE:	ORCA
PARTITIONING:	NoSpherA2
INT ACCURACY:	Normal
METHOD:	B3LYP
BASIS SET:	def2-TZVPP
CHARGE:	0
MULTIPLICITY:	1
DATE:	2021-12-25_21-30-30

To take the molecular displacement into account a libration correction was later applied to the structure. Two different programs where used: XP (LIBR ALL) and PLATON (CALC TMP).[9a-e] This leads to the following, corrected atomic bond distances:

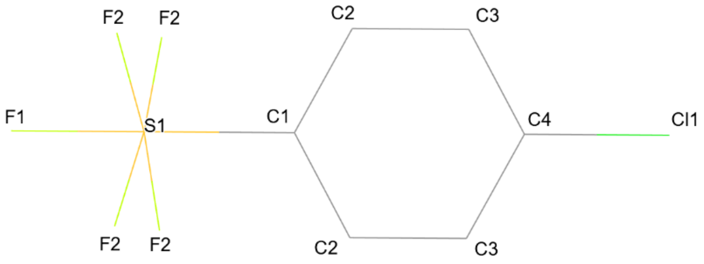

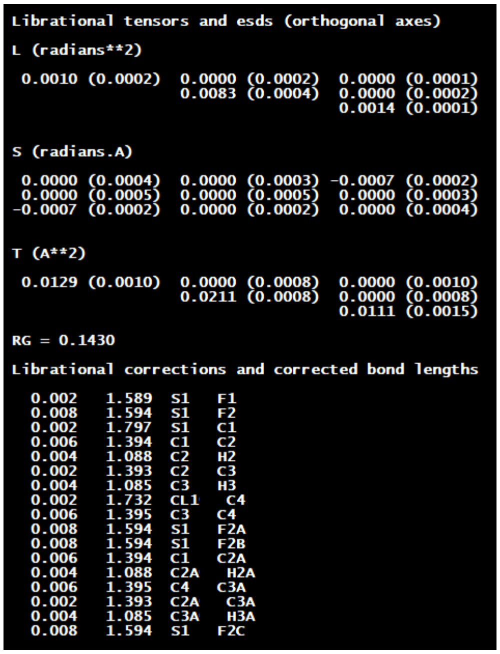

```
Librational tensors and esds (orthogonal axes)

L (radians**2)

 0.0010 (0.0002)  0.0000 (0.0002)  0.0000 (0.0001)
                  0.0083 (0.0004)  0.0000 (0.0002)
                                   0.0014 (0.0001)

S (radians.A)

 0.0000 (0.0004)  0.0000 (0.0003) -0.0007 (0.0002)
 0.0000 (0.0005)  0.0000 (0.0005)  0.0000 (0.0003)
-0.0007 (0.0002)  0.0000 (0.0002)  0.0000 (0.0004)

T (A**2)

 0.0129 (0.0010)  0.0000 (0.0008)  0.0000 (0.0010)
                  0.0211 (0.0008)  0.0000 (0.0008)
                                   0.0111 (0.0015)

RG = 0.1430

Librational corrections and corrected bond lengths

  0.002   1.589  S1   F1
  0.008   1.594  S1   F2
  0.002   1.797  S1   C1
  0.006   1.394  C1   C2
  0.004   1.088  C2   H2
  0.002   1.393  C2   C3
  0.004   1.085  C3   H3
  0.002   1.732  CL1   C4
  0.006   1.395  C3   C4
  0.008   1.594  S1   F2A
  0.008   1.594  S1   F2B
  0.006   1.394  C1   C2A
  0.004   1.088  C2A   H2A
  0.006   1.395  C4   C3A
  0.002   1.393  C2A   C3A
  0.004   1.085  C3A   H3A
  0.008   1.594  S1   F2C
```

Rigid-Body Model Libration Corrections for Bond Distances and "Hirshfeld Rigid-Bond" Test (Acta Cryst., 1976, A32, 239-244)

Bond		Bond Distance	Components of the Correction				MSDA from U(obs) Vibration Along the Interatomic Bond					Angle with Lib. Axes		
Atom(I)	Atom(J)	Obsd	Calcd	Del(L)	Del(M)	Del(N)	I to J	J to I	Difference		Sqrt(Diff)	L(1)	L(2)	L(3)
Cl1	- C4	1.7302(7)	1.7322	0.0020	0	0	0.01758(7)	0.01771(19)	0.0001(2)		0.0100	0.00	90.00	90.00
S1	- F1	1.5869(4)	1.5887	0.0018	0	0	0.01693(6)	0.01784(18)	0.00091(19)		0.0302	0.42	90.00	90.00
S1	- F2	1.5867(3)	1.5924	0.0001	-0.0041	0.0039	0.01638(6)	0.01721(9)	0.00083(11)	#	0.0288	87.44	45.10	45.01
S1	- C1	1.7952(6)	1.7973	-0.0020	0	0	0.01693(6)	0.01783(18)	0.00090(19)		0.0300	180.00	90.00	90.00
C1	- C2	1.3888(5)	1.3931	-0.0008	0.0045	0	0.01350(18)	0.01368(14)	0.0002(2)		0.0141	119.13	90.00	150.87
C2	- C3	1.3911(6)	1.3927	-0.0016	0	0	0.02003(14)	0.01993(15)	0.0001(2)		0.0100	179.69	90.00	89.98
C3	- C4	1.3894(5)	1.3936	-0.0008	-0.0045	0	0.02140(15)	0.02151(19)	0.0001(2)		0.0100	119.21	90.00	29.22

Sqrt(Sum(DelIJ**2)/Nrb) = 0.0006

# - Indicates bonds exceeding the 5.0 sigma test level

Figure S56. Libration corrections for compound **4**. Wire plot of compound 4 (top left), XP output (top right) and PLATON output (bottom).

Table S9. Crystal data and structure refinement of compound **4**

Identification code	14594	
Empirical formula	$C_6 H_4 Cl F_5 S$	
Color	colourless	
Formula weight	238.609 g·mol^{-1}	
Temperature	100(2) K	
Wavelength	0.71073 Å	
Crystal system	Orthorhombic	
Space group	*Cmcm*, (no. 63)	
Unit cell dimensions	a = 9.4508(11) Å	α= 90°.
	b = 12.6324(14) Å	β= 90°.
	c = 6.8646(8) Å	γ = 90°.
Volume	819.54(16) Å^3	
Z	4	
Density (calculated)	1.934 Mg·m^{-3}	
Absorption coefficient	0.751 mm^{-1}	
F(000)	473.506 e	
Crystal size	0.432 x 0.409 x 0.40 mm^3	
θ range for data collection	2.69 to 40.25°.	
Index ranges	$-17 \le h \le 16$, $-23 \le k \le 21$, $-12 \le l \le 12$	
Reflections collected	24861	
Independent reflections	1421 [R_{int} = 0.0288]	
Reflections with I>2σ(I)	1333	
Completeness to θ = 25.2417°	99.08 %	
Absorption correction	Gaussian	
Max. and min. transmission	0.79046 and 0.75517	
Refinement method	Full-matrix least-squares on F^2	
Data / restraints / parameters	1421 / 0 / 54	
Goodness-of-fit on F^2	1.0918	
Final R indices [I>2σ(I)]	R_1 = 0.0135	wR^2 = 0.0326
R indices (all data)	R_1 = 0.0148	wR^2 = 0.0332
Largest diff. peak and hole	0.2271 and -0.3263 e·Å^{-3}	
Special remarks	Aspherical atomic form factors have been used	

Table S10. Bond lengths [Å] and angles [°] of compound **4**

Cl(1)-C(4)	1.7302(6)	S(1)-F(1)	1.5869(5)
S(1)-F(2)#1	1.5867(2)	S(1)-F(2)#2	1.5867(2)
S(1)-F(2)	1.5867(2)	S(1)-F(2)#3	1.5867(2)
S(1)-C(1)	1.7952(6)	C(1)-C(2)#2	1.3888(5)
C(1)-C(2)	1.3888(5)	C(2)-C(3)	1.3911(6)
C(3)-C(4)	1.3893(5)		
F(2)#2-S(1)-F(1)	87.441(9)	F(2)#3-S(1)-F(1)	87.441(9)
F(2)-S(1)-F(1)	87.441(9)	F(2)#1-S(1)-F(1)	87.441(9)
F(2)#3-S(1)-F(2)#2	89.793(17)	F(2)#1-S(1)-F(2)#3	
174.881(17)	F(2)-S(1)-F(2)#2	174.881(17)	F(2)#3-
S(1)-F(2)	89.978(17)	F(2)#1-S(1)-F(2)#2	89.978(17)
F(2)#1-S(1)-F(2)	89.793(17)	C(1)-S(1)-F(1)	180.0
C(1)-S(1)-F(2)#3	92.559(9)	C(1)-S(1)-F(2)#1	92.559(9)
C(1)-S(1)-F(2)	92.559(9)	C(1)-S(1)-F(2)#2	92.559(9)
C(2)-C(1)-S(1)	119.13(3)	C(2)#2-C(1)-S(1)	119.13(3)
C(2)#2-C(1)-C(2)	121.73(5)	C(3)-C(2)-C(1)	119.12(4)
C(4)-C(3)-C(2)	119.23(4)	C(3)#2-C(4)-Cl(1)	119.21(3)
C(3)-C(4)-Cl(1)	119.21(3)	C(3)#2-C(4)-C(3)	121.57(5)

Symmetry transformations used to generate equivalent atoms:
#1 -x,-y,z+5/2   #2+3   #3 -x+1,y,-z+1/2

Single crystal structure analysis of compound 8

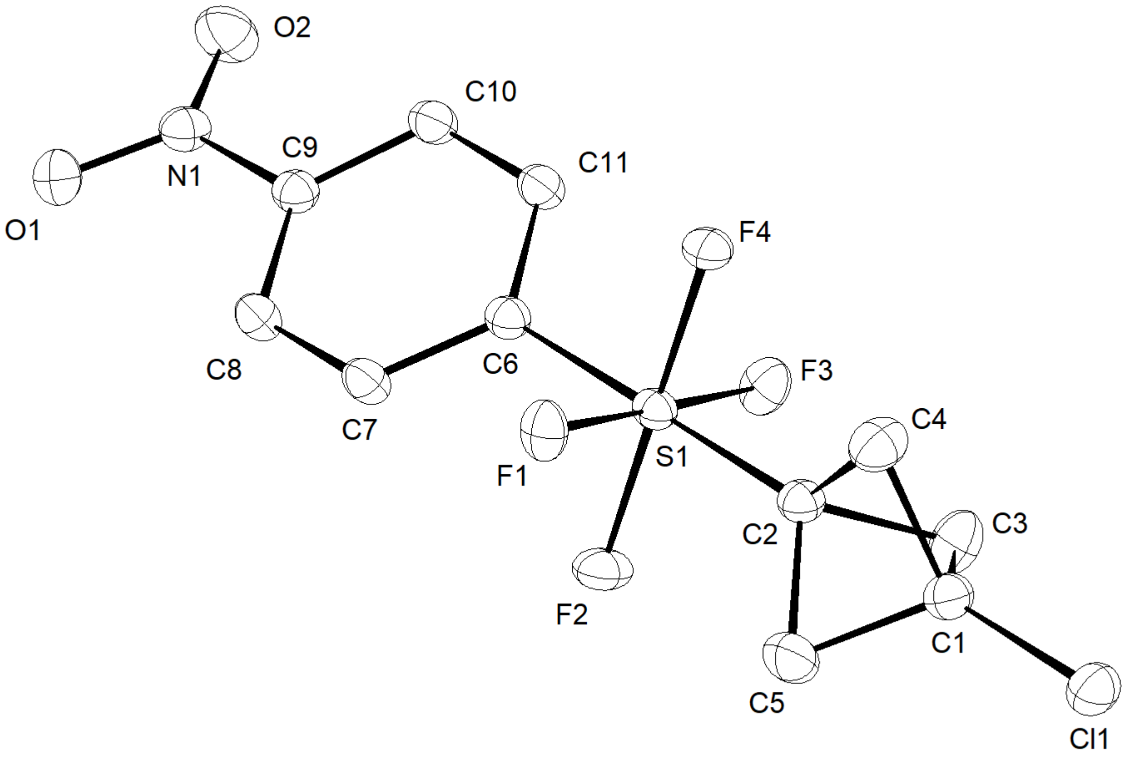

Figure S57. The molecular structure of compound **8**. H atoms have been removed for clarity.

X-ray Crystal Structure Analysis of compound 8:

C_{11} H_{10} Cl F_4 N O_2 S, M_r = 331.71 g mol^{-1}, colorless plate, crystal size 0.24 x 0.22 x 0.04 mm^3, Monoclinic, space group $P2_1/c$ [14], a = 9.0944(4) Å, b = 10.5692(4) Å, c = 13.3760(6) Å, β = 97.641(4)°, V = 1274.29(9) Å^3, T = 100(2) K, Z = 4, D_{calc} = 1.729 g·cm^3, λ = 0.71073 Å, μ(*Mo-K$_\alpha$*) = 0.513 mm^{-1}, Gaussian absorption correction ($T_{\min}$ = 0.95686, $T_{\max}$ = 0.97017), Bruker AXS Enraf-Nonius KappaCCD diffractometer with a FR591 rotating Mo-anode X-ray source, 2.970 < θ < 40.086°, 41921 measured reflections, 7928 independent reflections, 5022 reflections with $I > 2\sigma(I)$, R_{int} = 0.0529. The structure was solved by *SHELXS* and refined by full-matrix least-squares (*SHELXL*) against F^2 to R_1 = 0.0448 [$I > 2\sigma(I)$], wR_2 = 0.1173 [all data], 221parameters.

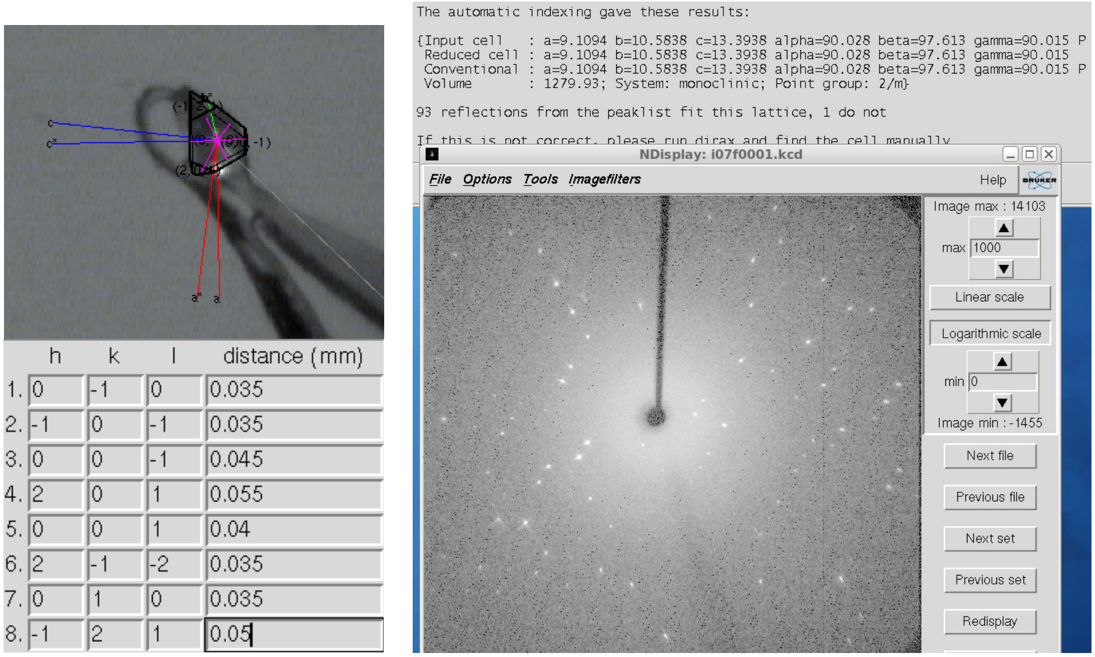

Figure S58. Crystal faces and unit cell determination/refinement of compound **8**.

INTENSITY STATISTICS FOR DATASET

Resolution	#Data	#Theory	%Complete	Redundancy	Mean I	Mean I/s	Rmerge	Rsigma
Inf - 2.27	126	133	94.7	6.85	83.77	70.72	0.0199	0.0099
2.27 - 1.51	293	293	100.0	7.62	51.50	63.44	0.0204	0.0104
1.51 - 1.20	421	421	100.0	7.59	33.09	54.21	0.0235	0.0122
1.20 - 1.05	396	396	100.0	7.29	19.52	40.12	0.0293	0.0157
1.05 - 0.95	425	425	100.0	6.78	11.24	28.09	0.0419	0.0226
0.95 - 0.88	414	414	100.0	6.34	9.23	23.41	0.0507	0.0282
0.88 - 0.83	396	396	100.0	6.17	8.06	20.97	0.0605	0.0335
0.83 - 0.78	491	491	100.0	5.64	5.87	15.95	0.0785	0.0459
0.78 - 0.75	362	362	100.0	5.43	5.65	14.48	0.0831	0.0512
0.75 - 0.72	439	439	100.0	5.12	5.13	12.39	0.0950	0.0604
0.72 - 0.69	502	502	100.0	4.98	4.22	10.27	0.1248	0.0766
0.69 - 0.67	392	392	100.0	4.70	3.14	7.47	0.1542	0.1099
0.67 - 0.65	422	422	100.0	4.60	2.63	6.21	0.1930	0.1436
0.65 - 0.63	501	501	100.0	4.32	2.21	4.75	0.2357	0.1959
0.63 - 0.62	272	272	100.0	4.30	1.55	3.28	0.3278	0.3006
0.62 - 0.60	591	593	99.7	4.08	1.47	2.80	0.3487	0.3645
0.60 - 0.59	323	325	99.4	3.94	1.28	2.09	0.4131	0.4942
0.59 - 0.58	351	351	100.0	3.81	1.05	1.55	0.4419	0.6710
0.58 - 0.57	373	373	100.0	3.76	1.01	1.38	0.5000	0.7767
0.57 - 0.56	394	401	98.3	3.54	1.06	1.30	0.4849	0.8689
0.56 - 0.55	360	367	98.1	3.34	1.00	0.98	0.5309	1.0528
0.65 - 0.55	3165	3183	99.4	3.90	1.37	2.38	0.3718	0.4950
Inf - 0.55	8244	8269	99.7	5.18	9.11	16.12	0.0500	0.0513

Complete .cif-data of the compound are available under the CCDC number **CCDC-2195539**.

To take the molecular displacement into account a libration correction was later applied to the structure. Two different programs where used: XP (LIBR ALL) and PLATON (CALC TMP).[9a-e] This leads to the following, corrected atomic bond distances:

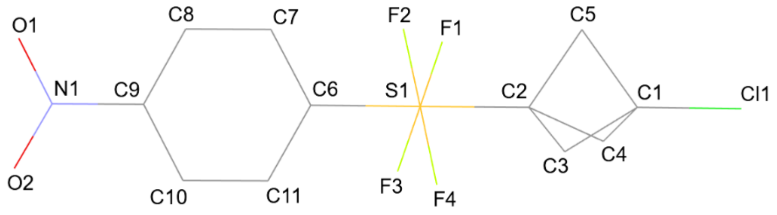


```
Librational tensors and esds (orthogonal axes)

L (radians**2)

 0.0015 (0.0002)  0.0001 (0.0001)  0.0018 (0.0002)
                  0.0005 (0.0001)  0.0001 (0.0001)
                                   0.0027 (0.0004)

S (radians.A)

-0.0001 (0.0005) -0.0001 (0.0003)  0.0001 (0.0003)
-0.0001 (0.0002) -0.0002 (0.0005)  0.0000 (0.0002)
 0.0001 (0.0003)  0.0000 (0.0004)  0.0002 (0.0006)

T (A**2)

 0.0188 (0.0012)  0.0012 (0.0011)  0.0043 (0.0010)
                  0.0185 (0.0013) -0.0010 (0.0010)
                                   0.0220 (0.0010)

RG = 0.2218

Librational corrections and corrected bond lengths

  0.004   1.634  S1   F1
  0.003   1.629  S1   F2
  0.004   1.626  S1   F3
  0.003   1.635  S1   F4
  0.002   1.226  O1   N1
  0.002   1.223  O2   N1
  0.001   1.752  CL1   C1
  0.001   1.808  S1   C2
  0.001   1.813  C1   C2
  0.002   1.545  C1   C3
  0.002   1.544  C2   C3
  0.003   1.552  C1   C4
  0.002   1.551  C2   C4
  0.002   1.541  C1   C5
  0.003   1.548  C2   C5
  0.001   1.821  S1   C6
  0.002   1.395  C6   C7
  0.001   1.389  C7   C8
  0.001   1.476  N1   C9
  0.003   1.385  C8   C9
  0.002   1.386  C9   C10
  0.003   1.396  C6   C11
  0.001   1.387  C10   C11
  0.002   0.940  C7   H7
  0.001   0.926  C11   H11
  0.002   1.016  C4   H4A
  0.002   0.988  C5   H5A
  0.002   0.936  C10   H10
  0.001   0.891  C8   H8
  0.002   1.013  C3   H3A
  0.002   0.995  C5   H5B
  0.002   0.979  C4   H4B
  0.002   1.004  C3   H3B
```

```
=============================================================================================================
Rigid-Body Model Libration Corrections for Bond Distances and "Hirshfeld Rigid-Bond" Test (Acta Cryst., 1976, A32, 239-244)
=============================================================================================================

                                                                MSDA from U(obs)
     Bond          Bond Distance   Components of the Correction  Vibration Along the Interatomic Bond       Angle with Lib. Axes

Atom(I)  Atom(J)        Obsd       Calcd  Del(L)  Del(M)  Del(N)    I to J      J to I    Difference  Sqrt(Diff)    L(1)   L(2)   L(3)
-------------------------------------------------------------------------------------------------------------
Cl1     - C4       1.7302(7)     1.7322  0.0020       0       0  0.01758(7) 0.01771(19)   0.0001(2)     0.0100      0.00  90.00  90.00
S1      - F1       1.5869(4)     1.5887  0.0018       0       0  0.01693(6) 0.01784(18) 0.00091(19)     0.0302      0.42  90.00  90.00
S1      - F2       1.5867(3)     1.5924  0.0001 -0.0041  0.0039  0.01638(6)  0.01721(9) 0.00083(11) #   0.0288     87.44  45.10  45.01
S1      - C1       1.7952(6)     1.7973 -0.0020       0       0  0.01693(6) 0.01783(18) 0.00090(19)     0.0300    180.00  90.00  90.00
C1      - C2       1.3888(5)     1.3931 -0.0008  0.0045       0 0.01350(18) 0.01368(14)   0.0002(2)     0.0141    119.13  90.00 150.87
C2      - C3       1.3911(6)     1.3927 -0.0016       0       0 0.02003(14) 0.01993(15)   0.0001(2)     0.0100    179.69  90.00  89.98
C3      - C4       1.3894(5)     1.3936 -0.0008 -0.0045       0 0.02140(15) 0.02151(19)   0.0001(2)     0.0100    119.21  90.00  29.22

                                                   Sqrt(Sum(DelIJ**2)/Nrb) =     0.0006

                                # - Indicates bonds exceeding the 5.0 sigma test level
```

Figure S59. Libration corrections for compound **8**. Wire plot of compound **8** (top left), XP output (top right) and PLATON output (bottom).

Table S11. Crystal data and structure refinement of compound **8**.

Identification code	14133	
Empirical formula	$C_{11} H_{10} Cl F_4 N O_2 S$	
Color	colourless	
Formula weight	331.71 g · mol^{-1}	
Temperature	100(2) K	
Wavelength	0.71073 Å	
Crystal system	Monoclinic	
Space group	$P2_1/c$, (no. 14)	
Unit cell dimensions	a = 9.0944(4) Å	α= 90°.
	b = 10.5692(4) Å	β= 97.641(4)°.
	c = 13.3760(6) Å	γ = 90°.
Volume	1274.29(9) Å^3	
Z	4	
Density (calculated)	1.729 Mg · m^{-3}	
Absorption coefficient	0.513 mm^{-1}	
F(000)	672 e	
Crystal size	0.1 x 0.07 x 0.07 mm^3	
θ range for data collection	2.970 to 40.086°.	
Index ranges	$-16 \le h \le 16$, $-18 \le k \le 19$, $-24 \le l \le 23$	
Reflections collected	41921	
Independent reflections	7928 [R_{int} = 0.0529]	
Reflections with I>2σ(I)	5022	
Completeness to θ = 25.242°	99.8 %	
Absorption correction	Gaussian	
Max. and min. transmission	0.97 and 0.96	
Refinement method	Full-matrix least-squares on F^2	
Data / restraints / parameters	7928 / 0 / 221	
Goodness-of-fit on F^2	1.021	
Final R indices [I>2σ(I)]	R_1 = 0.0448	wR^2 = 0.1034
R indices (all data)	R_1 = 0.0913	wR^2 = 0.1173
Largest diff. peak and hole	0.5 and -0.4 e · Å^{-3}	

Table S12. Bond lengths [Å] and angles [°] of compound **8**.

Cl(1)-C(1)	1.7517(12)	S(1)-F(1)	1.6302(8)
S(1)-F(2)	1.6252(7)	S(1)-F(3)	1.6220(8)
S(1)-F(4)	1.6319(7)	S(1)-C(2)	1.8078(11)
S(1)-C(6)	1.8199(11)	O(1)-N(1)	1.2244(13)
O(2)-N(1)	1.2202(14)	N(1)-C(9)	1.4759(15)
C(1)-C(2)	1.8122(16)	C(1)-C(3)	1.5422(18)
C(1)-C(4)	1.5489(18)	C(1)-C(5)	1.5391(18)
C(2)-C(3)	1.5415(18)	C(2)-C(4)	1.5486(17)
C(2)-C(5)	1.5453(17)	C(3)-H(3A)	1.011(19)
C(3)-H(3B)	1.002(19)	C(4)-H(4A)	1.01(2)
C(4)-H(4B)	0.977(19)	C(5)-H(5A)	0.99(2)
C(5)-H(5B)	0.99(2)	C(6)-C(7)	1.3926(15)
C(6)-C(11)	1.3932(16)	C(7)-C(8)	1.3884(16)
C(7)-H(7)	0.939(18)	C(8)-C(9)	1.3826(16)
C(8)-H(8)	0.889(18)	C(9)-C(10)	1.3837(16)
C(10)-C(11)	1.3869(17)	C(10)-H(10)	0.934(19)
C(11)-H(11)	0.925(17)		
F(1)-S(1)-F(4)	89.77(4)	F(1)-S(1)-C(2)	89.61(5)
F(1)-S(1)-C(6)	90.30(4)	F(2)-S(1)-F(1)	90.45(4)
F(2)-S(1)-F(4)	179.74(5)	F(2)-S(1)-C(2)	89.98(5)
F(2)-S(1)-C(6)	89.86(4)	F(3)-S(1)-F(1)	179.43(4)
F(3)-S(1)-F(2)	89.97(4)	F(3)-S(1)-F(4)	89.81(4)
F(3)-S(1)-C(2)	90.78(5)	F(3)-S(1)-C(6)	89.31(5)
F(4)-S(1)-C(2)	90.17(4)	F(4)-S(1)-C(6)	89.99(4)
C(2)-S(1)-C(6)	179.81(5)	O(1)-N(1)-C(9)	117.97(10)
O(2)-N(1)-O(1)	124.02(11)	O(2)-N(1)-C(9)	118.00(10)
Cl(1)-C(1)-C(2)	178.43(9)	C(3)-C(1)-Cl(1)	124.45(9)
C(3)-C(1)-C(2)	53.99(7)	C(3)-C(1)-C(4)	88.84(10)
C(4)-C(1)-Cl(1)	126.77(9)	C(4)-C(1)-C(2)	54.19(7)
C(5)-C(1)-Cl(1)	126.41(9)	C(5)-C(1)-C(2)	54.18(7)
C(5)-C(1)-C(3)	89.53(10)	C(5)-C(1)-C(4)	89.00(10)
S(1)-C(2)-C(1)	178.77(8)	C(3)-C(2)-S(1)	125.26(9)

C(3)-C(2)-C(1)	54.03(7)	C(3)-C(2)-C(4)	88.87(10)
C(3)-C(2)-C(5)	89.33(10)	C(4)-C(2)-S(1)	127.03(8)
C(4)-C(2)-C(1)	54.20(7)	C(5)-C(2)-S(1)	125.63(9)
C(5)-C(2)-C(1)	53.86(7)	C(5)-C(2)-C(4)	88.78(10)
C(1)-C(3)-H(3A)	115.5(11)	C(1)-C(3)-H(3B)	110.2(11)
C(2)-C(3)-C(1)	71.98(9)	C(2)-C(3)-H(3A)	114.2(11)
C(2)-C(3)-H(3B)	110.9(10)	H(3A)-C(3)-H(3B)	123.0(15)
C(1)-C(4)-H(4A)	117.0(10)	C(1)-C(4)-H(4B)	113.7(11)
C(2)-C(4)-C(1)	71.61(8)	C(2)-C(4)-H(4A)	116.3(11)
C(2)-C(4)-H(4B)	115.0(11)	H(4A)-C(4)-H(4B)	115.9(15)
C(1)-C(5)-C(2)	71.96(9)	C(1)-C(5)-H(5A)	109.2(12)
C(1)-C(5)-H(5B)	112.3(12)	C(2)-C(5)-H(5A)	109.1(12)
C(2)-C(5)-H(5B)	114.7(12)	H(5A)-C(5)-H(5B)	126.6(17)
C(7)-C(6)-S(1)	119.76(8)	C(7)-C(6)-C(11)	121.58(10)
C(11)-C(6)-S(1)	118.61(8)	C(6)-C(7)-H(7)	120.7(11)
C(8)-C(7)-C(6)	119.08(10)	C(8)-C(7)-H(7)	120.3(11)
C(7)-C(8)-H(8)	118.4(12)	C(9)-C(8)-C(7)	118.46(11)
C(9)-C(8)-H(8)	123.0(12)	C(8)-C(9)-N(1)	118.74(10)
C(8)-C(9)-C(10)	123.15(11)	C(10)-C(9)-N(1)	118.09(10)
C(9)-C(10)-C(11)	118.26(10)	C(9)-C(10)-H(10)	121.3(11)
C(11)-C(10)-H(10)	120.4(11)	C(6)-C(11)-H(11)	121.5(10)
C(10)-C(11)-C(6)	119.31(10)	C(10)-C(11)-H(11)	119.2(10)

Single crystal structure analysis of compound 7

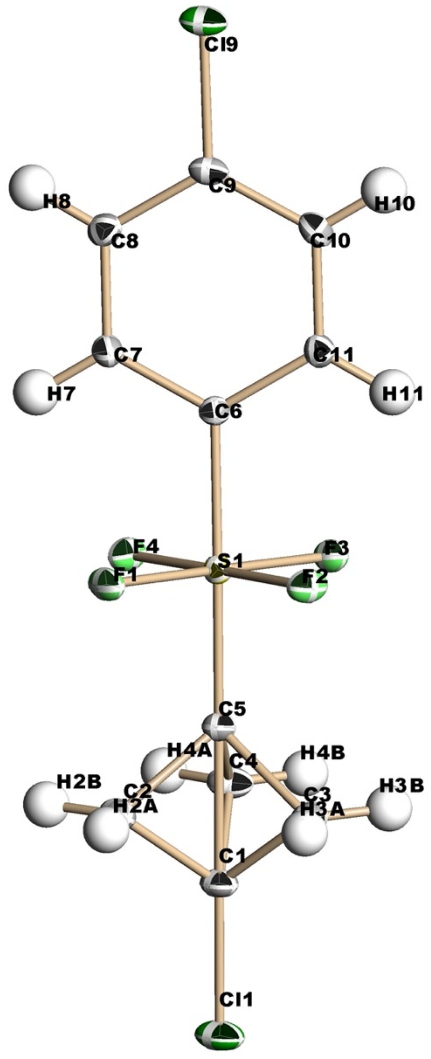

Figure S60. The molecular structure of compound **7**.

X-ray Crystal Structure Analysis of compound 7:

A colorless block with approximate orthogonal dimensions 0.171 x 0.217 x 0.374mm^3 was placed and optically centered on the Bruker[1] APEXII CCD system at –183°C(90K). Indexing of the unit cell used a random set of reflections collected from three series of 0.5° wide ω-scans, 10 seconds per frame, and 30 frames per series that were well distributed in reciprocal space. Eight ω-scan data frame series were collected [MoK$_{\alpha}$] with 0.3° wide scans, 10 seconds per frame and 606 frames collected per series with the detector set at 32.0° at varying φ angles (φ=0°, 90°, 180°, 270°) and the detector set at -32.0° at varying φ angles (φ=45°, 135, 225, 315°) due to a bad

quadrant on the detector. The crystal to detector distance was 5.00cm, thus providing a complete sphere of data to $2\theta_{max}$=61.44°.

Structural determination and Refinement:

The SHELXTL[11] program package was implemented to determine the probable space group and set up the initial files. System symmetry, systematic absences and intensity statistics indicated the standard centrosymmetric monoclinic space group $P2_1/c$ (no. 14). The structure was determined by direct methods with nearly all non-hydrogen atoms being located directly for the molecule using the program XT.[12] The structure was refined with XL.[13] The 31268 data collected were merged, based upon identical indices, to 15000 data, then truncated to $2\theta_{max}$=60.00° and 13462 data, and then merged for least squares refinement to 3665 unique data [R(int)=0.0189]. All non-hydrogen atoms were refined anisotropically. All hydrogen atoms were initially idealized and then allowed to refine freely during the final refinement stage. The final structure was refined to convergence with R(F)=2.85%, wR(F^2)=6.41%, GOF=1.126 for all 3665 unique reflections [R(F)=2.53, wR(F^2)=6.27% for those 3331 data with Fo > 4σ(Fo)]. The final difference-Fourier map was featureless indicating that the structure is both correct and complete. An empirical correction for extinction was also attempted but found to be less than one sigma and therefore not applied.

Table S13. Crystal data and structure refinement of compound **7**.

Identification code	JF3122FMI (YK-I-145)	
Empirical formula	C11 H10 Cl2 F4 S	
Formula weight	321.15	
Temperature	90(2) K	
Wavelength	0.71073 Å	
Crystal system	Monoclinic	
Space group	$P2_1/c$	
Unit cell dimensions	a = 5.8570(2) Å	α= 90°.
	b = 7.4279(2) Å	β= 94.5123(16)°.
	c = 28.8221(9) Å	γ = 90°.
Volume	1250.02(7) $Å^3$	
Z	4	
Density (calculated)	1.706 Mg/m^3	
Absorption coefficient	0.713 mm^{-1}	
F(000)	648	
Crystal size	0.374 x 0.217 x 0.171 mm^3	
Crystal color and habit	Colorless Block	
Diffractometer	Bruker APEX-II CCD	
Theta range for data collection	1.417 to 29.999°.	

Index ranges	-8<=h<=8, -10<=k<=10, -40<=l<=40
Reflections collected	13642
Independent reflections	3665 [R(int) = 0.0189]
Observed reflections (I > 2sigma(I))	3331
Completeness to theta = 25.242°	99.9 %
Absorption correction	Semi-empirical from equivalents
Max. and min. transmission	0.8763 and 0.7975
Solution method	SHELXT (Sheldrick, 2014)
Refinement method	SHELXL-2018/3 (Sheldrick, 2018) Full-matrix least-squares on F^2
Data / restraints / parameters	3665 / 0 / 203
Goodness-of-fit on F^2	1.126
Final R indices [I>2sigma(I)]	R1 = 0.0253, wR2 = 0.0627
R indices (all data)	R1 = 0.0285, wR2 = 0.0641
Largest diff. peak and hole	0.480 and -0.259 e.Å^{-3}

Table S14. Bond lengths [Å] and angles [°] of compound **7**.

S(1)-F(1)	1.6200(7)
S(1)-F(2)	1.6223(7)
S(1)-F(3)	1.6322(7)
S(1)-F(4)	1.6336(7)
S(1)-C(5)	1.8111(11)
S(1)-C(6)	1.8170(11)
C(1)-C(4)	1.5449(17)
C(1)-C(2)	1.5460(16)
C(1)-C(3)	1.5518(17)
C(1)-Cl(1)	1.7659(12)
C(1)-C(5)	1.8094(16)
C(2)-C(5)	1.5470(16)
C(2)-H(2A)	0.921(16)
C(2)-H(2B)	0.969(16)
C(3)-C(5)	1.5487(16)
C(3)-H(3A)	0.985(16)
C(3)-H(3B)	0.966(16)
C(4)-C(5)	1.5471(16)
C(4)-H(4A)	0.960(16)
C(4)-H(4B)	0.953(17)
C(6)-C(11)	1.3907(15)
C(6)-C(7)	1.3949(15)
C(7)-C(8)	1.3910(16)
C(7)-H(7)	0.955(14)
C(8)-C(9)	1.3878(16)
C(8)-H(8)	0.951(16)
C(9)-C(10)	1.3888(16)
C(9)-Cl(9)	1.7352(11)
C(10)-C(11)	1.3949(15)
C(10)-H(10)	0.914(16)
C(11)-H(11)	0.950(16)
F(1)-S(1)-F(2)	89.68(4)

F(1)-S(1)-F(3)	179.10(4)
F(2)-S(1)-F(3)	89.49(4)
F(1)-S(1)-F(4)	90.30(4)
F(2)-S(1)-F(4)	179.39(4)
F(3)-S(1)-F(4)	90.53(4)
F(1)-S(1)-C(5)	91.02(4)
F(2)-S(1)-C(5)	91.29(4)
F(3)-S(1)-C(5)	88.65(4)
F(4)-S(1)-C(5)	88.09(4)
F(1)-S(1)-C(6)	90.11(4)
F(2)-S(1)-C(6)	91.04(4)
F(3)-S(1)-C(6)	90.25(4)
F(4)-S(1)-C(6)	89.57(4)
C(5)-S(1)-C(6)	177.41(5)
C(4)-C(1)-C(2)	89.19(9)
C(4)-C(1)-C(3)	89.29(9)
C(2)-C(1)-C(3)	89.35(9)
C(4)-C(1)-Cl(1)	125.65(8)
C(2)-C(1)-Cl(1)	125.89(8)
C(3)-C(1)-Cl(1)	125.79(9)
C(4)-C(1)-C(5)	54.24(7)
C(2)-C(1)-C(5)	54.22(7)
C(3)-C(1)-C(5)	54.22(7)
Cl(1)-C(1)-C(5)	179.87(9)
C(1)-C(2)-C(5)	71.61(8)
C(1)-C(2)-H(2A)	116.3(10)
C(5)-C(2)-H(2A)	115.1(10)
C(1)-C(2)-H(2B)	116.2(10)
C(5)-C(2)-H(2B)	115.6(10)
H(2A)-C(2)-H(2B)	115.0(14)
C(5)-C(3)-C(1)	71.40(8)
C(5)-C(3)-H(3A)	115.0(9)
C(1)-C(3)-H(3A)	117.1(9)
C(5)-C(3)-H(3B)	115.3(10)
C(1)-C(3)-H(3B)	118.2(10)
H(3A)-C(3)-H(3B)	113.5(13)

C(1)-C(4)-C(5)	71.63(8)
C(1)-C(4)-H(4A)	116.7(10)
C(5)-C(4)-H(4A)	115.7(10)
C(1)-C(4)-H(4B)	116.2(10)
C(5)-C(4)-H(4B)	115.1(10)
H(4A)-C(4)-H(4B)	114.7(14)
C(2)-C(5)-C(4)	89.07(9)
C(2)-C(5)-C(3)	89.43(9)
C(4)-C(5)-C(3)	89.33(9)
C(2)-C(5)-C(1)	54.17(7)
C(4)-C(5)-C(1)	54.13(7)
C(3)-C(5)-C(1)	54.38(7)
C(2)-C(5)-S(1)	126.15(8)
C(4)-C(5)-S(1)	124.49(8)
C(3)-C(5)-S(1)	126.66(8)
C(1)-C(5)-S(1)	178.56(8)
C(11)-C(6)-C(7)	120.97(10)
C(11)-C(6)-S(1)	119.74(8)
C(7)-C(6)-S(1)	119.29(8)
C(8)-C(7)-C(6)	119.50(10)
C(8)-C(7)-H(7)	119.9(9)
C(6)-C(7)-H(7)	120.6(9)
C(9)-C(8)-C(7)	119.33(10)
C(9)-C(8)-H(8)	120.2(9)
C(7)-C(8)-H(8)	120.5(9)
C(8)-C(9)-C(10)	121.43(10)
C(8)-C(9)-Cl(9)	119.26(9)
C(10)-C(9)-Cl(9)	119.29(9)
C(9)-C(10)-C(11)	119.31(10)
C(9)-C(10)-H(10)	120.9(10)
C(11)-C(10)-H(10)	119.7(10)
C(6)-C(11)-C(10)	119.41(10)
C(6)-C(11)-H(11)	120.2(9)
C(10)-C(11)-H(11)	120.4(9)

Single crystal structure analysis of compound 9

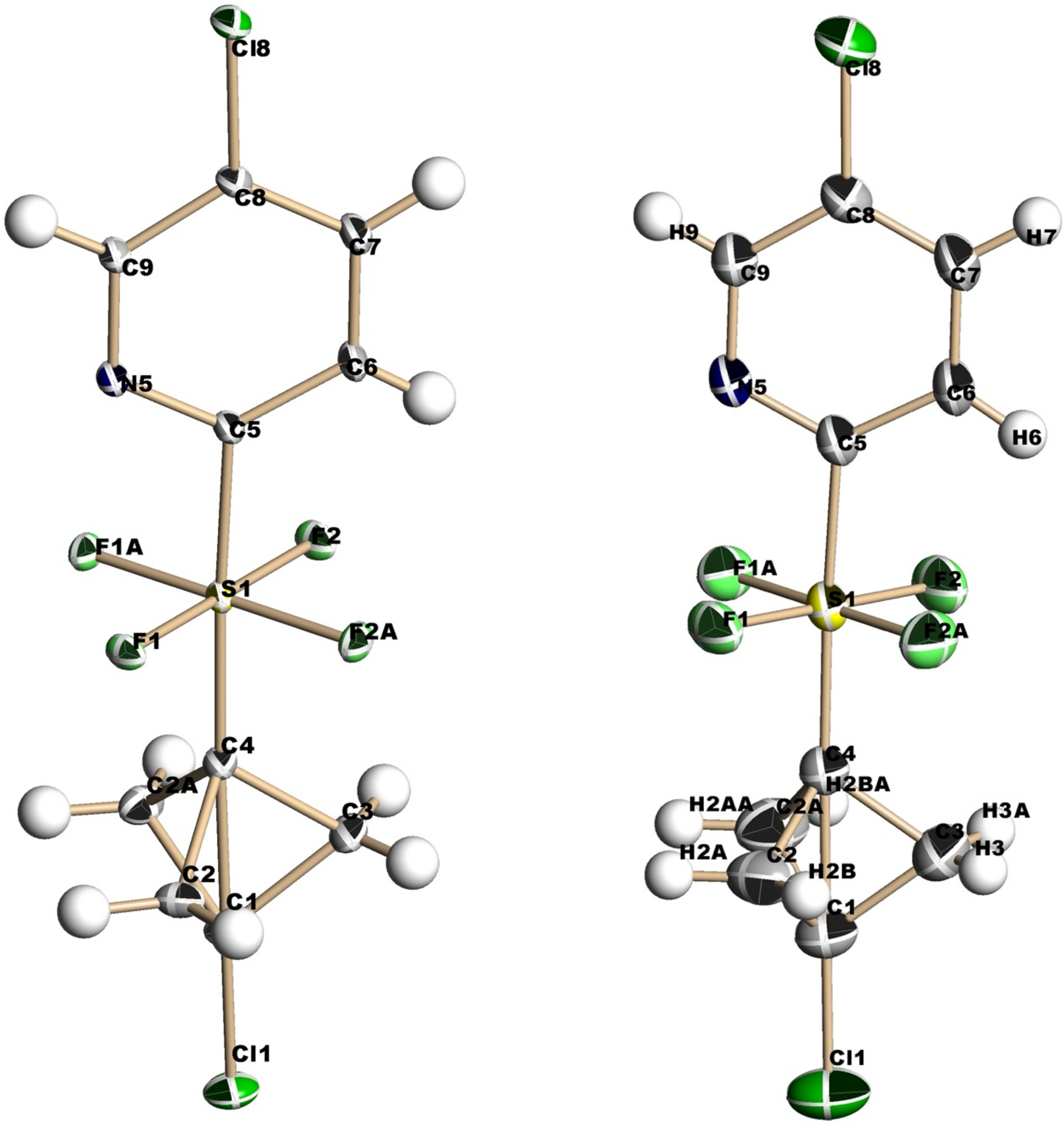

Figure S61. The molecular structure of compound **9** at 90 K (left) and 240 K (right).

A colorless block with approximate orthogonal dimensions 0.131 x 0.218 x 0.284mm^3 was placed and optically centered on the Bruker[Error! Bookmark not defined.] APEXII CCD system at –33°C (240K). Indexing of the unit cell used a random set of reflections collected from three series of 0.5° wide ω-scans, 10 seconds per frame, and 30 frames per series that were well distributed in reciprocal space. Eight ω-scan data frame series were collected [MoK$_\alpha$] with 0.3° wide scans, 10 seconds per frame and 606 frames collected per series with the detector set at 32.0° at varying φ angles (φ=0°, 90°, 180°, 270°) and the detector set at -32.0° at varying φ angles (φ=45°, 135°, 225°, 315°) due

to a bad quadrant on the detector. The crystal to detector distance was 5.00cm, thus providing a complete sphere of data to $2\theta_{max}$=61.58°.

Structural determination and Refinement:

The SHELXTL[11] program package was implemented to determine the probable space group and set up the initial files. System symmetry, systematic absences and intensity statistics indicated the non-centrosymmetric orthorhombic space group Pbcm (no. 57). The structure was determined by direct methods with the non-hydrogen atoms being located using the program XT[12]. The structure was refined with XL[13]. The 31926 data collected were merged based upon identical indices to 14282, then merged for least squares refinement to 2090 unique data [R(int)=0.0206]. All non-hydrogen atoms were refined anisotropically. Hydrogen atoms were located directly from a difference-Fourier map and allowed to refine freely throughout the final refinement stages. The final structure was refined to convergence with R(F)=3.37%, wR(F^2)=8.17%, GOF=1.028 for all 2090 unique reflections [R(F)=2.98, wR(F^2)=7.84 for those 1849 data with Fo > 4σ(Fo)]. The final difference-Fourier map was featureless indicating that the structure is both correct and complete. An empirical correction for extinction was also attempted and found to be less than three sigma and therefore not applied.

Table S15. Crystal data and structure refinement of compound **9**.

Identification code	JF3121_90K_FMI	(ANK-I-052)
Empirical formula	C10 H9 Cl2 F4 N S	
Formula weight	322.14	
Temperature	**90(2) K**	
Wavelength	0.71073 Å	
Crystal system	Orthorhombic	
Space group	Pbcm	
Unit cell dimensions	a = 6.1409(2) Å	α= 90°.
	b = 26.9844(8) Å	β= 90°.
	c = 7.3502(2) Å	γ = 90°.
Volume	1217.99(6) $Å^3$	
Z	4	
Density (calculated)	1.757 Mg/m^3	
Absorption coefficient	0.734 mm^{-1}	
F(000)	648	
Crystal size	0.284 x 0.218 x 0.131 mm^3	
Crystal color and habit	Colorless Block	
Diffractometer	Bruker APEX-II CCD	
Theta range for data collection	1.509 to 30.762°.	

Index ranges	-8<=h<=8, -38<=k<=38, -10<=l<=10
Reflections collected	13905
Independent reflections	2035 [R(int) = 0.0183]
Observed reflections (I > 2sigma(I))	1931
Completeness to theta = 25.242°	100.0 %
Absorption correction	Semi-empirical from equivalents
Max. and min. transmission	0.8932 and 0.8265
Solution method	SHELXT (Sheldrick, 2014)
Refinement method	SHELXL-2018/3 (Sheldrick, 2018) Full-matrix least-squares on F^2
Data / restraints / parameters	2035 / 0 / 121
Goodness-of-fit on F^2	1.039
Final R indices [I>2sigma(I)]	R1 = 0.0229, wR2 = 0.0631
R indices (all data)	R1 = 0.0240, wR2 = 0.0638
Largest diff. peak and hole	0.457 and -0.407 e.Å^{-3}

Table S16. Bond lengths [Å] and angles [°] of compound **9**.

S(1)-F(1)	1.6213(6)
S(1)-F(1)#1	1.6213(6)
S(1)-F(2)	1.6371(6)
S(1)-F(2)#1	1.6371(6)
S(1)-C(4)	1.8119(12)
S(1)-C(5)	1.8386(13)
Cl(1)-C(1)	1.7648(14)
C(1)-C(3)	1.545(2)
C(1)-C(2)#1	1.5476(13)
C(1)-C(2)	1.5476(13)
C(1)-C(4)	1.8123(18)
C(2)-C(4)	1.5480(13)
C(2)-H(2A)	0.962(15)
C(2)-H(2B)	0.976(14)
C(3)-C(4)	1.5494(18)
C(3)-H(3)	0.964(15)
C(3)-H(3)#1	0.964(15)
C(5)-N(5)	1.3194(16)
C(5)-C(6)	1.3853(17)
N(5)-C(9)	1.3435(16)
C(6)-C(7)	1.3880(19)
C(6)-H(6)	0.89(2)
C(7)-C(8)	1.3855(18)
C(7)-H(7)	0.957(19)
C(8)-C(9)	1.3892(17)
C(8)-Cl(8)	1.7296(13)
C(9)-H(9)	0.94(2)
F(1)-S(1)-F(1)#1	90.12(4)
F(1)-S(1)-F(2)	179.85(4)
F(1)#1-S(1)-F(2)	89.77(3)
F(1)-S(1)-F(2)#1	89.77(3)
F(1)#1-S(1)-F(2)#1	179.85(4)

F(2)-S(1)-F(2)#1	90.34(4)
F(1)-S(1)-C(4)	91.21(3)
F(1)#1-S(1)-C(4)	91.21(3)
F(2)-S(1)-C(4)	88.69(4)
F(2)#1-S(1)-C(4)	88.68(4)
F(1)-S(1)-C(5)	90.66(4)
F(1)#1-S(1)-C(5)	90.66(4)
F(2)-S(1)-C(5)	89.45(4)
F(2)#1-S(1)-C(5)	89.45(4)
C(4)-S(1)-C(5)	177.36(6)
C(3)-C(1)-C(2)#1	89.28(8)
C(3)-C(1)-C(2)	89.28(8)
C(2)#1-C(1)-C(2)	89.17(10)
C(3)-C(1)-Cl(1)	125.71(10)
C(2)#1-C(1)-Cl(1)	125.84(7)
C(2)-C(1)-Cl(1)	125.84(7)
C(3)-C(1)-C(4)	54.26(7)
C(2)#1-C(1)-C(4)	54.18(6)
C(2)-C(1)-C(4)	54.18(6)
Cl(1)-C(1)-C(4)	179.97(10)
C(1)-C(2)-C(4)	71.67(7)
C(1)-C(2)-H(2A)	116.4(9)
C(4)-C(2)-H(2A)	115.8(9)
C(1)-C(2)-H(2B)	117.3(8)
C(4)-C(2)-H(2B)	116.5(8)
H(2A)-C(2)-H(2B)	113.2(12)
C(1)-C(3)-C(4)	71.70(9)
C(1)-C(3)-H(3)	115.3(9)
C(4)-C(3)-H(3)	114.2(8)
C(1)-C(3)-H(3)#1	115.3(9)
C(4)-C(3)-H(3)#1	114.2(8)
H(3)-C(3)-H(3)#1	117.8(18)
C(2)-C(4)-C(2)#1	89.15(10)
C(2)-C(4)-C(3)	89.11(7)
C(2)#1-C(4)-C(3)	89.11(7)
C(2)-C(4)-S(1)	126.75(6)

C(2)#1-C(4)-S(1)	126.75(6)
C(3)-C(4)-S(1)	124.12(9)
C(2)-C(4)-C(1)	54.16(5)
C(2)#1-C(4)-C(1)	54.15(5)
C(3)-C(4)-C(1)	54.04(8)
S(1)-C(4)-C(1)	178.16(9)
N(5)-C(5)-C(6)	125.22(12)
N(5)-C(5)-S(1)	115.60(9)
C(6)-C(5)-S(1)	119.18(10)
C(5)-N(5)-C(9)	117.38(10)
C(5)-C(6)-C(7)	117.57(12)
C(5)-C(6)-H(6)	122.2(14)
C(7)-C(6)-H(6)	120.3(14)
C(8)-C(7)-C(6)	117.91(11)
C(8)-C(7)-H(7)	121.2(12)
C(6)-C(7)-H(7)	120.9(12)
C(7)-C(8)-C(9)	120.34(12)
C(7)-C(8)-Cl(8)	120.07(10)
C(9)-C(8)-Cl(8)	119.59(10)
N(5)-C(9)-C(8)	121.57(12)
N(5)-C(9)-H(9)	116.7(12)
C(8)-C(9)-H(9)	121.7(12)

Symmetry transformations used to generate equivalent atoms:

#1 x,y,-z+1/2

Single crystal structure analysis of compound 17

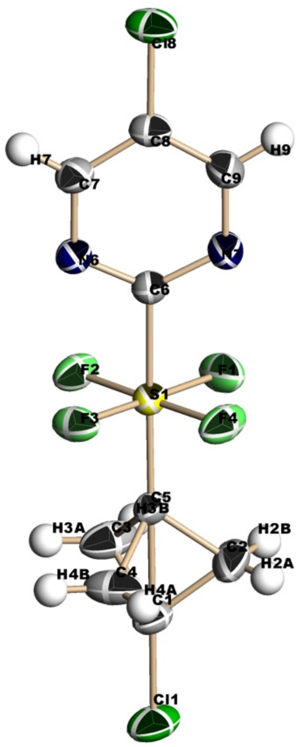

Figure S62. The molecular structure of compound **9** at 90 K (left) and 240 K (right).

Single crystal structure analysis of compound 17

A colorless block with approximate orthogonal dimensions 0.222 x 0.355 x 0.426mm^3 was placed and optically centered on the Bruker[Error! Bookmark not defined.] Venture Dual source Kappa diffractometer with Photon2 CMOS detector at –93°C(180K). Indexing of the unit cell used a random set of reflections collected from three series of 0.5° wide ω-scans, 5 seconds per frame, and 30 frames per series that were well distributed in reciprocal space. Data were collected [MoKα] with 0.3° wide scans, variable time per frame dependent upon detector 2θ angle and varying φ and omega angles such that nearly all unique reflections were collected at least once. The crystal to detector distance was 65.00mm, thus providing a complete sphere of data to $2\theta_{max}$=61.01°.

Structural determination and Refinement:

The SHELXTL[11] program package was implemented to determine the probable space group and set up the initial files. System symmetry, systematic absences and intensity statistics indicated the standard centrosymmetric monoclinic space group $P2_1/n$ (no. 14). The structure was determined by direct methods with nearly all non-hydrogen atoms being located directly for the molecule using

the program XT[12]. The structure was refined with XL[13]. The 41957 data collected were merged, based upon identical indices, to 13815 data, and then merged for least squares refinement to 3821 unique data [R(int)=0.0185]. A disorder for the C2, C3, C4 group was optimized to be 0.88:0.12. All non-hydrogen atoms were refined anisotropically. All hydrogen atoms were initially idealized and then those fully occupied had their thermal parameters refined during the final refinement stage. The final structure was refined to convergence with R(F)=3.83%, wR(F^2)=8.40%, GOF=1.109 for all 3751 unique reflections [R(F)=3.12, wR(F^2)=8.12% for those 3150 data with Fo > 4σ(Fo)]. The final difference-Fourier map was featureless indicating that the structure is both correct and complete. An empirical correction for extinction was also attempted but found to be negative and therefore not applied.

Table S17. Crystal data and structure refinement of compound **17**.

Identification code	JF3119FMI	
Empirical formula	C9 H8 Cl2 F4 N2 S	
Formula weight	323.13	
Temperature	**180(2) K**	
Wavelength	0.71073 Å	
Crystal system	Monoclinic	
Space group	$P2_1/n$	
Unit cell dimensions	a = 6.1176(3) Å	α= 90°.
	b = 23.9975(11) Å	β= 102.3627(15)°.
	c = 8.5600(4) Å	γ = 90°.
Volume	1227.53(10) $Å^3$	
Z	4	
Density (calculated)	1.748 Mg/m^3	
Absorption coefficient	0.730 mm^{-1}	
F(000)	648	
Crystal size	0.426 x 0.355 x 0.222 mm^3	
Crystal color and habit	Colorless Block	
Diffractometer	Bruker Photon2 CMOS	
Theta range for data collection	2.580 to 30.520°.	
Index ranges	-8<=h<=8, -34<=k<=34, -12<=l<=12	
Reflections collected	13815	
Independent reflections	3751 [R(int) = 0.0185]	
Observed reflections (I > 2sigma(I))	3150	
Completeness to theta = 25.242°	99.9 %	
Absorption correction	Semi-empirical from equivalents	

Max. and min. transmission	0.8374 and 0.7685
Solution method	SHELXT (Sheldrick, 2014)
Refinement method	SHELXL-2018/3 (Sheldrick, 2018) Full-matrix least-squares on F^2
Data / restraints / parameters	3751 / 15 / 177
Goodness-of-fit on F^2	1.109
Final R indices [I>2sigma(I)]	R1 = 0.0312, wR2 = 0.0812
R indices (all data)	R1 = 0.0383, wR2 = 0.0840
Largest diff. peak and hole	0.270 and -0.365 e.Å^{-3}

Table S18. Bond lengths [Å] and angles [°] of compound **17**.

S(1)-F(3)	1.6109(9)
S(1)-F(2)	1.6135(9)
S(1)-F(1)	1.6142(9)
S(1)-F(4)	1.6211(9)
S(1)-C(5)	1.8064(13)
S(1)-C(6)	1.8466(13)
Cl(1)-C(1)	1.7641(14)
Cl(8)-C(8)	1.7232(14)
C(1)-C(3B)	1.48(3)
C(1)-C(3)	1.532(2)
C(1)-C(4B)	1.54(3)
C(1)-C(2)	1.537(2)
C(1)-C(4)	1.538(2)
C(1)-C(2B)	1.54(2)
C(1)-C(5)	1.8021(18)
C(2)-C(5)	1.533(2)
C(2)-H(2A)	0.9900
C(2)-H(2B)	0.9900
C(3)-C(5)	1.535(2)
C(3)-H(3A)	0.9900
C(3)-H(3B)	0.9900
C(4)-C(5)	1.538(2)
C(4)-H(4A)	0.9900
C(4)-H(4B)	0.9900
C(2B)-C(5)	1.57(2)

C(2B)-H(2B1)	0.9900
C(2B)-H(2B2)	0.9900
C(3B)-C(5)	1.52(3)
C(3B)-H(3B1)	0.9900
C(3B)-H(3B2)	0.9900
C(4B)-C(5)	1.56(3)
C(4B)-H(4B1)	0.9900
C(4B)-H(4B2)	0.9900
C(6)-N(6)	1.3150(17)
C(6)-N(7)	1.3154(18)
N(6)-C(7)	1.3364(18)
C(7)-C(8)	1.378(2)
C(7)-H(7)	0.9500
N(7)-C(9)	1.342(2)
C(8)-C(9)	1.372(2)
C(9)-H(9)	0.9500
F(3)-S(1)-F(2)	89.73(5)
F(3)-S(1)-F(1)	179.10(6)
F(2)-S(1)-F(1)	89.42(5)
F(3)-S(1)-F(4)	89.68(6)
F(2)-S(1)-F(4)	178.65(5)
F(1)-S(1)-F(4)	91.16(6)
F(3)-S(1)-C(5)	90.72(5)
F(2)-S(1)-C(5)	91.49(5)
F(1)-S(1)-C(5)	89.62(5)
F(4)-S(1)-C(5)	89.74(5)
F(3)-S(1)-C(6)	89.66(5)
F(2)-S(1)-C(6)	89.45(5)
F(1)-S(1)-C(6)	90.02(6)
F(4)-S(1)-C(6)	89.33(5)
C(5)-S(1)-C(6)	178.99(6)
C(3B)-C(1)-C(4B)	90.9(13)
C(3)-C(1)-C(2)	89.35(13)
C(3)-C(1)-C(4)	88.72(12)
C(2)-C(1)-C(4)	89.08(13)

C(3B)-C(1)-C(2B)	90.8(13)
C(4B)-C(1)-C(2B)	88.8(11)
C(3B)-C(1)-Cl(1)	125.7(12)
C(3)-C(1)-Cl(1)	125.30(11)
C(4B)-C(1)-Cl(1)	125.4(11)
C(2)-C(1)-Cl(1)	126.31(11)
C(4)-C(1)-Cl(1)	126.19(11)
C(2B)-C(1)-Cl(1)	124.3(10)
C(3B)-C(1)-C(5)	54.1(12)
C(3)-C(1)-C(5)	54.10(9)
C(4B)-C(1)-C(5)	55.1(11)
C(2)-C(1)-C(5)	53.97(9)
C(4)-C(1)-C(5)	54.13(8)
C(2B)-C(1)-C(5)	55.4(10)
Cl(1)-C(1)-C(5)	179.40(11)
C(5)-C(2)-C(1)	71.89(11)
C(5)-C(2)-H(2A)	116.4
C(1)-C(2)-H(2A)	116.4
C(5)-C(2)-H(2B)	116.4
C(1)-C(2)-H(2B)	116.4
H(2A)-C(2)-H(2B)	113.4
C(1)-C(3)-C(5)	71.98(10)
C(1)-C(3)-H(3A)	116.4
C(5)-C(3)-H(3A)	116.4
C(1)-C(3)-H(3B)	116.4
C(5)-C(3)-H(3B)	116.4
H(3A)-C(3)-H(3B)	113.4
C(1)-C(4)-C(5)	71.74(10)
C(1)-C(4)-H(4A)	116.4
C(5)-C(4)-H(4A)	116.4
C(1)-C(4)-H(4B)	116.4
C(5)-C(4)-H(4B)	116.4
H(4A)-C(4)-H(4B)	113.4
C(1)-C(2B)-C(5)	70.9(9)
C(1)-C(2B)-H(2B1)	116.5
C(5)-C(2B)-H(2B1)	116.5

C(1)-C(2B)-H(2B2)	116.5
C(5)-C(2B)-H(2B2)	116.5
H(2B1)-C(2B)-H(2B2)	113.5
C(1)-C(3B)-C(5)	73.8(10)
C(1)-C(3B)-H(3B1)	116.1
C(5)-C(3B)-H(3B1)	116.1
C(1)-C(3B)-H(3B2)	116.1
C(5)-C(3B)-H(3B2)	116.1
H(3B1)-C(3B)-H(3B2)	113.1
C(1)-C(4B)-C(5)	71.1(9)
C(1)-C(4B)-H(4B1)	116.5
C(5)-C(4B)-H(4B1)	116.5
C(1)-C(4B)-H(4B2)	116.5
C(5)-C(4B)-H(4B2)	116.5
H(4B1)-C(4B)-H(4B2)	113.5
C(2)-C(5)-C(3)	89.35(13)
C(2)-C(5)-C(4)	89.21(13)
C(3)-C(5)-C(4)	88.59(12)
C(3B)-C(5)-C(4B)	88.4(12)
C(3B)-C(5)-C(2B)	88.2(12)
C(4B)-C(5)-C(2B)	86.7(11)
C(3B)-C(5)-C(1)	52.1(12)
C(2)-C(5)-C(1)	54.14(9)
C(3)-C(5)-C(1)	53.92(9)
C(4)-C(5)-C(1)	54.13(9)
C(4B)-C(5)-C(1)	53.7(11)
C(2B)-C(5)-C(1)	53.8(10)
C(3B)-C(5)-S(1)	128.0(12)
C(2)-C(5)-S(1)	125.77(11)
C(3)-C(5)-S(1)	126.14(10)
C(4)-C(5)-S(1)	125.89(10)
C(4B)-C(5)-S(1)	126.2(11)
C(2B)-C(5)-S(1)	126.2(10)
C(1)-C(5)-S(1)	179.91(10)
N(6)-C(6)-N(7)	128.89(13)
N(6)-C(6)-S(1)	115.71(10)

N(7)-C(6)-S(1)	115.40(10)
C(6)-N(6)-C(7)	115.38(12)
N(6)-C(7)-C(8)	121.03(13)
N(6)-C(7)-H(7)	119.5
C(8)-C(7)-H(7)	119.5
C(6)-N(7)-C(9)	115.23(13)
C(9)-C(8)-C(7)	118.39(13)
C(9)-C(8)-Cl(8)	120.24(11)
C(7)-C(8)-Cl(8)	121.38(11)
N(7)-C(9)-C(8)	121.07(14)
N(7)-C(9)-H(9)	119.5
C(8)-C(9)-H(9)	119.5

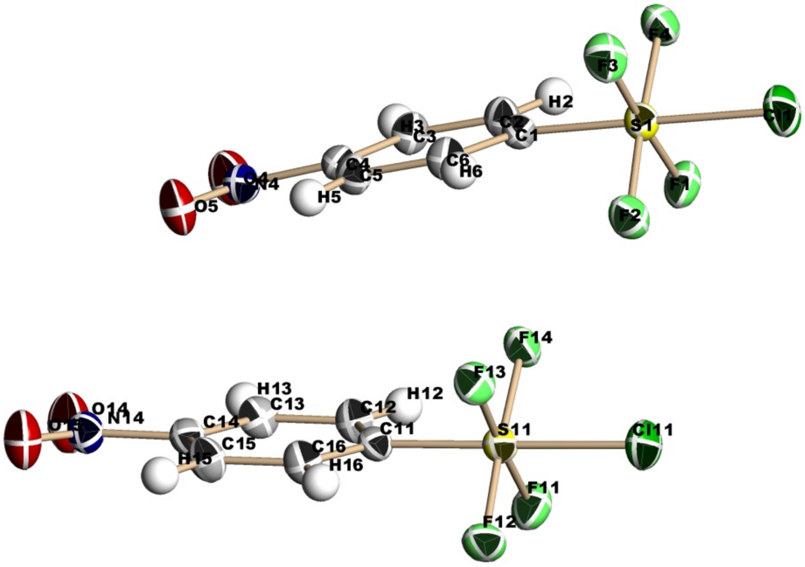

Figure S63. The molecular structure of compound **18**.

Single crystal structure analysis of compound 18

A colorless block with approximate orthogonal dimensions 0.258 x 0.403 x 0.434mm^3 was placed and optically centered on the Bruker Duo[Error! Bookmark not defined.] APEXII CCD system at –183°C(90K). Indexing of the unit cell used a random set of reflections collected from three series of 0.5° wide ω-scans, 10 seconds per frame, and 30 frames per series that were well distributed in reciprocal space. Five ω-scan data frame series were collected [MoK$_{\alpha}$] with 0.3° wide scans, 20 seconds per frame and 606 frames collected per series at varying φ angles (φ=0°, 72°, 144°, 216°,

288°). The crystal to detector distance was 5.15cm, thus providing a complete sphere of data to $2\theta_{max}$=61.63°.

Structural determination and Refinement:

The SHELXTL[11] program package was implemented to determine the probable space group and set up the initial files. System symmetry, lack of systematic absences and intensity statistics indicated the centrosymmetric triclinic space group P-1 (no. 2). The structure was determined by direct methods with the location of nearly all of the non-hydrogen atoms for the molecule using the program XT[12]. The structure was refined with XL[13]. The 18032 data collected were merged for identical indices to 11227 reflections, truncated to 55.00 and 8382 data, then merged for least squares refinement to 4217 unique data [R(int)=0.0204]. Non-hydrogen atoms were refined anisotropically. Hydrogen atoms were initially idealized and then allowed to refine freely during the final refinement. The final structure was refined to convergence with R(F)=4.48%, wR(F^2)=10.20%, GOF=1.038 for all 4217 unique reflections [R(F)=3.47, wR(F^2)=9.51% for those 3391 data with Fo > 4σ(Fo)]. The final difference-Fourier map was featureless indicating that the structure is both correct and complete.

Table S19. Crystal data and structure refinement of compound **18**.

Identification code	JF3077FMI	
Empirical formula	C6 H4 Cl F4 N O2 S	
Formula weight	265.61	
Temperature	190(2) K	
Wavelength	0.71073 Å	
Crystal system	Triclinic	
Space group	P-1	
Unit cell dimensions	a = 8.5404(13) Å	α= 77.879(2)°.
	b = 10.5584(16) Å	β= 70.328(2)°.
	c = 11.3105(17) Å	γ = 73.751(2)°.
Volume	914.4(2) $Å^3$	
Z, Z'	4, 2	
Density (calculated)	1.929 Mg/m^3	
Absorption coefficient	0.688 mm^{-1}	
F(000)	528	
Crystal size	0.434 x 0.403 x 0.258 mm^3	
Crystal color and habit	Colorless Block	
Diffractometer	Bruker APEX-II CCD	
Theta range for data collection	1.928 to 27.516°.	
Index ranges	-11<=h<=11, -13<=k<=13, -14<=l<=14	

Reflections collected	8382
Independent reflections	4217 [R(int) = 0.0204]
Observed reflections (I > 2sigma(I))	3391
Completeness to theta = 25.242°	100.0 %
Absorption correction	Semi-empirical from equivalents
Max. and min. transmission	0.8301 and 0.7241
Solution method	SHELXT (Sheldrick, 2014)
Refinement method	SHELXL-2018/3 (Sheldrick, 2018) Full-matrix least-squares on F^2
Data / restraints / parameters	4217 / 0 / 303
Goodness-of-fit on F^2	1.038
Final R indices [I>2sigma(I)]	R1 = 0.0347, wR2 = 0.0951
R indices (all data)	R1 = 0.0448, wR2 = 0.1020
Largest diff. peak and hole	0.523 and -0.290 e.$Å^{-3}$

Table S20. Bond lengths [Å] and angles [°] of compound **18**.

C(1)-C(2)	1.390(2)
C(1)-C(6)	1.390(2)
C(1)-S(1)	1.8158(17)
C(2)-C(3)	1.382(3)
C(2)-H(2)	0.96(2)
C(3)-C(4)	1.382(2)
C(3)-H(3)	0.87(2)
C(4)-C(5)	1.389(2)
C(4)-N(4)	1.481(2)
N(4)-O(5)	1.223(2)
N(4)-O(4)	1.223(2)
C(5)-C(6)	1.389(3)
C(5)-H(5)	0.90(2)
C(6)-H(6)	0.92(2)
S(1)-F(2)	1.5943(11)
S(1)-F(1)	1.5977(11)
S(1)-F(3)	1.5995(11)
S(1)-F(4)	1.6055(11)
S(1)-Cl(1)	2.0845(7)
C(11)-C(16)	1.387(2)
C(11)-C(12)	1.394(2)
C(11)-S(11)	1.8157(17)
C(12)-C(13)	1.391(3)
C(12)-H(12)	0.93(2)
C(13)-C(14)	1.385(2)
C(13)-H(13)	0.89(2)
C(14)-C(15)	1.381(2)
C(14)-N(14)	1.478(2)
N(14)-O(14)	1.222(2)
N(14)-O(15)	1.223(2)
C(15)-C(16)	1.392(3)
C(15)-H(15)	0.93(2)
C(16)-H(16)	0.94(2)
S(11)-F(13)	1.5972(11)

S(11)-F(11)	1.6013(12)
S(11)-F(12)	1.6035(11)
S(11)-F(14)	1.6042(11)
S(11)-Cl(11)	2.0807(7)
C(2)-C(1)-C(6)	121.68(16)
C(2)-C(1)-S(1)	119.24(13)
C(6)-C(1)-S(1)	119.00(13)
C(3)-C(2)-C(1)	119.32(16)
C(3)-C(2)-H(2)	121.9(14)
C(1)-C(2)-H(2)	118.8(14)
C(4)-C(3)-C(2)	118.55(16)
C(4)-C(3)-H(3)	119.4(15)
C(2)-C(3)-H(3)	122.0(15)
C(3)-C(4)-C(5)	123.04(17)
C(3)-C(4)-N(4)	118.74(15)
C(5)-C(4)-N(4)	118.22(15)
O(5)-N(4)-O(4)	123.76(16)
O(5)-N(4)-C(4)	118.25(14)
O(4)-N(4)-C(4)	117.98(14)
C(6)-C(5)-C(4)	118.04(16)
C(6)-C(5)-H(5)	117.6(14)
C(4)-C(5)-H(5)	124.1(14)
C(5)-C(6)-C(1)	119.31(16)
C(5)-C(6)-H(6)	120.3(14)
C(1)-C(6)-H(6)	120.2(14)
F(2)-S(1)-F(1)	90.14(6)
F(2)-S(1)-F(3)	90.02(7)
F(1)-S(1)-F(3)	177.18(6)
F(2)-S(1)-F(4)	176.70(6)
F(1)-S(1)-F(4)	89.66(6)
F(3)-S(1)-F(4)	90.03(6)
F(2)-S(1)-C(1)	91.49(7)
F(1)-S(1)-C(1)	91.34(7)
F(3)-S(1)-C(1)	91.47(7)
F(4)-S(1)-C(1)	91.80(7)

F(2)-S(1)-Cl(1)	88.25(5)
F(1)-S(1)-Cl(1)	88.30(5)
F(3)-S(1)-Cl(1)	88.90(5)
F(4)-S(1)-Cl(1)	88.46(5)
C(1)-S(1)-Cl(1)	179.55(5)
C(16)-C(11)-C(12)	121.87(16)
C(16)-C(11)-S(11)	119.13(13)
C(12)-C(11)-S(11)	118.98(13)
C(13)-C(12)-C(11)	119.15(16)
C(13)-C(12)-H(12)	119.7(14)
C(11)-C(12)-H(12)	121.1(14)
C(14)-C(13)-C(12)	118.21(16)
C(14)-C(13)-H(13)	121.6(13)
C(12)-C(13)-H(13)	120.2(13)
C(15)-C(14)-C(13)	123.24(17)
C(15)-C(14)-N(14)	118.24(15)
C(13)-C(14)-N(14)	118.52(15)
O(14)-N(14)-O(15)	123.94(16)
O(14)-N(14)-C(14)	117.93(15)
O(15)-N(14)-C(14)	118.12(15)
C(14)-C(15)-C(16)	118.44(17)
C(14)-C(15)-H(15)	124.3(15)
C(16)-C(15)-H(15)	117.2(15)
C(11)-C(16)-C(15)	119.09(17)
C(11)-C(16)-H(16)	118.6(15)
C(15)-C(16)-H(16)	122.1(15)
F(13)-S(11)-F(11)	177.05(6)
F(13)-S(11)-F(12)	89.80(6)
F(11)-S(11)-F(12)	89.95(6)
F(13)-S(11)-F(14)	90.01(6)
F(11)-S(11)-F(14)	90.07(6)
F(12)-S(11)-F(14)	176.87(6)
F(13)-S(11)-C(11)	91.71(7)
F(11)-S(11)-C(11)	91.23(7)
F(12)-S(11)-C(11)	91.72(7)
F(14)-S(11)-C(11)	91.41(6)

F(13)-S(11)-Cl(11)	88.39(5)
F(11)-S(11)-Cl(11)	88.66(5)
F(12)-S(11)-Cl(11)	88.85(5)
F(14)-S(11)-Cl(11)	88.02(4)
C(11)-S(11)-Cl(11)	179.42(6)

____

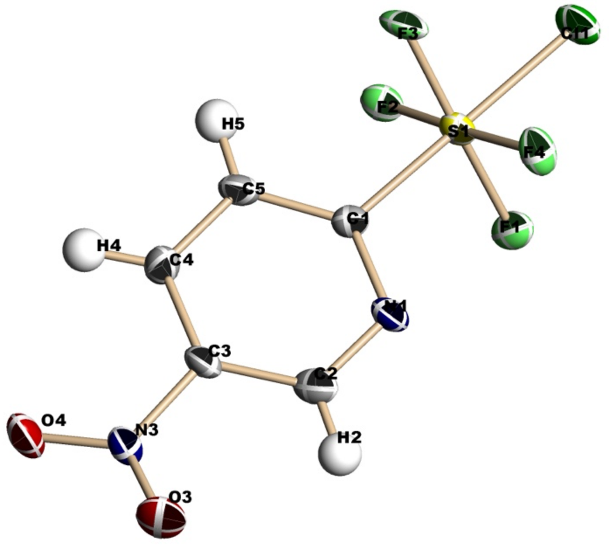

Figure S64. Molecular structure of compound **19**.

Single crystal structure analysis of compound 19

A colorless block with approximate orthogonal dimensions 0.095 x 0.104 x 0.188mm^3 was placed and optically centered on the Bruker[Error! Bookmark not defined.] Venture Dual Source Kappa Photon2 diffractometer at –173°C(100K). Indexing of the unit cell used a random set of reflections collected from three series of 0.5° wide ω-scans, 10 seconds per frame, and 30 frames per series that were well distributed in reciprocal space. Data were collected [MoKα] with 0.3° wide scans, variable time per frame dependent upon detector 2θ angle and varying φ and omega angles such that nearly all unique reflections were collected at least once. The crystal to detector distance was 65.00mm, thus providing a complete sphere of data to $2\theta_{max}$= 55.06°.

Structural determination and Refinement:

The SHELXTL[11] program package was implemented to determine the probable space group and set up the initial files. System symmetry, systematic absences and intensity statistics indicated the non-centrosymmetric monoclinic space group $P2_1$ (no. 4). The structure was determined by direct methods with the non-hydrogen atoms being located directly using the program XT[12]. The structure was refined with XL[13]. The 8698 data collected were merged, based upon identical

indices to 3736 data, then for least squares refinement to 2029 unique data [R(int)=0.0361]. All non-hydrogen atoms were refined anisotropically. Hydrogen atoms were idealized throughout the final refinement stages. The final structure was refined to convergence with R(F)=7.90%, wR(F^2)=16.91%, GOF=1200 for all 2029 unique reflections [R(F)=6.82, wR(F^2)=16.07% for those 1827 data with Fo > 4σ(Fo)]. The final difference-Fourier map was featureless indicating that the structure is both correct and complete. An empirical correction for extinction was also attempted and found to be negative and therefore not applied. The structure was refined as an inversion twin due to the absolute structure parameters varying far from zero. They were determined to be: Flack(x)[14], 0.1(3); Hooft(y)[8], 0.11(5) and Parsons(z)[15a,b], -0.01(5) indicating that the structure's absolute configuration has been determined reliably; these values would be close to 1.0 if the structure were inverted.

Table S21. Crystal data and structure refinement of compound **19**.

Identification code	JF3095FFMI	
Empirical formula	C5 H3 Cl F4 N2 O2 S	
Formula weight	266.60	
Temperature	100(2) K	
Wavelength	0.71073 Å	
Crystal system	Monoclinic	
Space group	$P2_1$	
Unit cell dimensions	a = 6.2717(10) Å	α= 90°.
	b = 9.6406(15) Å	β= 103.334(5)°.
	c = 7.5988(12) Å	γ = 90°.
Volume	447.06(12) $Å^3$	
Z	2	
Density (calculated)	1.981 Mg/m^3	
Absorption coefficient	0.707 mm^{-1}	
F(000)	264	
Crystal size	0.188 x 0.104 x 0.095 mm^3	
Crystal color and habit	Yellow Block	
Diffractometer	Bruker Photon2 CMOS	
Theta range for data collection	2.755 to 27.474°.	
Index ranges	-8<=h<=8, -12<=k<=12, -9<=l<=9	
Reflections collected	3736	
Independent reflections	2029 [R(int) = 0.0361]	
Observed reflections (I > 2sigma(I))	1827	
Completeness to theta = 25.242°	99.8 %	

Absorption correction	Semi-empirical from equivalents
Max. and min. transmission	0.7063 and 0.4482
Solution method	SHELXT (Sheldrick, 2014)
Refinement method	SHELXL-2018/3 (Sheldrick, 2018) Full-matrix least-squares on F^2
Data / restraints / parameters	2029 / 1 / 137
Goodness-of-fit on F^2	1.200
Final R indices [I>2sigma(I)]	R1 = 0.0682, wR2 = 0.1607
R indices (all data)	R1 = 0.0790, wR2 = 0.1691
Absolute structure parameter	0.1(3)
Largest diff. peak and hole	0.678 and -0.641 e.$Å^{-3}$

Table S22. Bond lengths [Å] and angles [°] of compound **19**.

S(1)-F(2)	1.589(6)
S(1)-F(4)	1.590(6)
S(1)-F(3)	1.597(6)
S(1)-F(1)	1.599(6)
S(1)-C(1)	1.831(10)
S(1)-C(l1)	2.066(3)
C(1)-N(1)	1.330(12)
C(1)-C(5)	1.385(14)
N(1)-C(2)	1.339(13)
C(2)-C(3)	1.378(15)
C(2)-H(2)	0.9500
C(3)-C(4)	1.374(14)
C(3)-N(3)	1.470(12)
N(3)-O(3)	1.223(11)
N(3)-O(4)	1.242(11)
C(4)-C(5)	1.394(13)
C(4)-H(4)	0.9500
C(5)-H(5)	0.9500
F(2)-S(1)-F(4)	177.3(4)
F(2)-S(1)-F(3)	89.8(3)

F(4)-S(1)-F(3)	90.3(4)
F(2)-S(1)-F(1)	89.2(3)
F(4)-S(1)-F(1)	90.6(3)
F(3)-S(1)-F(1)	178.2(4)
F(2)-S(1)-C(1)	90.9(4)
F(4)-S(1)-C(1)	91.9(4)
F(3)-S(1)-C(1)	90.8(4)
F(1)-S(1)-C(1)	90.8(4)
F(2)-S(1)-Cl(1)	88.5(2)
F(4)-S(1)-Cl(1)	88.8(3)
F(3)-S(1)-Cl(1)	89.4(2)
F(1)-S(1)-Cl(1)	89.0(2)
C(1)-S(1)-Cl(1)	179.3(4)
N(1)-C(1)-C(5)	126.4(9)
N(1)-C(1)-S(1)	114.1(7)
C(5)-C(1)-S(1)	119.5(7)
C(1)-N(1)-C(2)	116.9(9)
N(1)-C(2)-C(3)	120.3(10)
N(1)-C(2)-H(2)	119.8
C(3)-C(2)-H(2)	119.8
C(4)-C(3)-C(2)	122.9(9)
C(4)-C(3)-N(3)	119.5(8)
C(2)-C(3)-N(3)	117.6(9)
O(3)-N(3)-O(4)	124.3(8)
O(3)-N(3)-C(3)	118.7(8)
O(4)-N(3)-C(3)	117.0(8)
C(3)-C(4)-C(5)	117.1(10)
C(3)-C(4)-H(4)	121.4
C(5)-C(4)-H(4)	121.4
C(1)-C(5)-C(4)	116.4(9)
C(1)-C(5)-H(5)	121.8

C(8)-C(5)-H(5)	121.8

Computer used for crystallographic calculations:

All crystallographic calculations were performed on a Surface Pro7 with Intel i7-1065G7 at 1.30GHz with four cores, eight processors and 16GB of extended memory. Data collected were corrected for Lorentz and polarization effects with Saint[16] and absorption using Blessing's method and merged as incorporated with the program Sadabs.[17a,b]

References

[1] a) G. R. Fulmer, A. J. M. Miller, N. H. Sherden, H. E. Gottlieb, A. Nudelman, B. M. Stolz, J. E. Bercaw, K. I. Goldberg, *Organometallics* **2010**, *29*, 2176-2179; b) C. P. Rosenau, B. J. Jelier, A. D. Gossert, A. Togni, *Angew. Chem. Int. Ed.* **2018**, *57*, 9528-9533.

[2] a) C. R. Pitts, D. Bornemann, P. Liebing, N. Santschi, A. Togni, *Angew. Chem. Int. Ed.* **2018**, *58,* 1950-1954; b) B. Cui, M. Kosobokov, K. Matsuxaki, E. Tokunaga, N. Shibata, *Chem. Commun.* **2017**, *53*, 59997-6000; c) B. Zeynizadeh, *J. Chem. Res*. **2002**, *11,* 564-566; d) X. Zhang, N. Zhang, G. Chen, A. Turpoff, H. Ren, J. Takasugi, C. Morrill, J. Zhu, C. Li, W. Lennox, S. Paget, Y. Liu, N. Almstead, F. G. Njoroge, Z. Gu, T. Komatsu, V. Clausen, C. Espiritu, J. Graci, J. Colacino, F. Lahser, N. Risher, M. Weetall, A. Nomeir, G. M. Karp, *Bioorg. Med. Chem. Lett.* **2013**, *23*, 3947-3953.

[3] R. Gianatassio, J. M. Lopchuk, J. Wang, C.-M. Pan, L. R. Malins, L. Prieto, T. A. Brandt, M. R. Collins, G. M. Gallego, N. W. Sach, J. E. Spangler, H. Zhu, J. Zhu, P. S. Baran, *Science* **2016**, *351*, 241-246.

[4] a) C. R. Pitts, N. Santschi, A. Togni, WO2019229103, **2019**; b) J.-Y. Shou, X.-H. Xu, F.-L. Qing, *Angew. Chem. Int. Ed.* **2021**, *60*, 15271-15275.

[5] M. J. Frisch, G. W. Trucks, H. B. Schlegel, G. E. Scuseria, M. A. Robb, J. R. Cheeseman, G. Scalmani, V. Barone, G. A. Petersson, H. Nakatsuji, X. Li, M. Caricato, A. V. Marenich, J. Bloino, B. G. Janesko, R. Gomperts, B. Mennucci, H. P. Hratchian, J. V. Ortiz, A. F. Izmaylov, J. L. Sonnenberg, D. Williams-Young, F. Ding, F. Lipparini, F. Egidi, J. Goings, B. Peng, A. Petrone, T. Henderson, D. Ranasinghe, V. G. Zakrzewski, J. Gao, N. Rega, G. Zheng, W. Liang, M. Hada, M. Ehara, K. Toyota, R. Fukuda, J. Hasegawa, M. Ishida, T. Nakajima, Y. Honda, O. Kitao, H. Nakai, T. Vreven, K. Throssell, J. A. Montgomery, Jr., J. E. Peralta, F. Ogliaro, M. J. Bearpark, J. J. Heyd, E. N. Brothers, K. N. Kudin, V. N. Staroverov, T. A. Keith, R. Kobayashi, J. Normand, K. Raghavachari, A. P. Rendell, J. C. Burant, S. S. Iyengar, J. Tomasi, M. Cossi, J. M. Millam, M. Klene, C. Adamo, R. Cammi, J. W. Ochterski, R. L. Martin, K. Morokuma, O. Farkas, J. B. Foresman, and D. J. Fox, Gaussian, Inc., Wallingford CT, **2016**.

[6] a) A. D. McLean, G. S. Chandler, *J. Chem. Phys.* **1980**, *72*, 5639-5648; b) D. E. Woon, T. H. Dunning Jr., *J. Chem. Phys.* **1993**, *98*, 1358-1371.

[7] J.-D. Chai, M. Head-Gordon, *Phys. Chem. Chem. Phys.* **2008**, *10*, 6615-6620.

[8] IQmol. http://iqmol.org/ (accessed 08 August 2022).

[9] a) A. L. Spek, **2003**, *J. Appl. Cryst.,* 36, 7-11; b) V. Schomaker, K. N. Trueblood **1968**, *Acta Cryst.*, B24, 63-76; c) R. E. Rosenfield Jnr, K. N. Trueblood, J. D. Dunitz **1978**, *Acta Cryst.* A34, 828-829; d) XP - Interactive Molecular Graphics, Version 5.1, **1998** Bruker AXS; e) PLATON - A Multipurpose Crystallographic Tool, Version 110220, **1980-2020**, A. L. Spek

[10] a) R.W. Seidel, R. Goddard, N. Nöthling, C. W. Lehmann **2016,** *Acta Cryst.C*, 72(10), 753-757; b)R.W. Seidel, R. Goddard, N. Nöthling, C. W. Lehmann **2017,** *Acta Cryst.C*, 73(11), 937-940; c) R.W. Seidel, R. Goddard, N. Nöthling, C. W. Lehmann **2017,** *CrystEngComm*, 21(21), 3295-3303; d) M. Buchsteiner, L. Martinez-Rodriguez, P. Jerabek, M. Patzer, N. Nöthling, C. W. Lehmann, A. Fürstner **2020,** *Chem. Eur. J*, 26(11), 2509-2515; e) A. Bodach, N. Nöthling, M. Felderhoff **2021**, *Eur. J. Inorg. Chem*., 2021, 1240–1243; f) M. Patzer, N. Nöthling, R. Goddard, C. W. Lehmann **2021**, *Chemistry*, 3, 578–584.

[11] Sheldrick, G.M., (2002). SHELXTL. Version 6.1. Bruker AXS Inc., Madison, Wisconsin, USA.

[12] Sheldrick, G. M., (2014) SHELXT, Universität Göttingen: Göttingen, Germany. Structure determination program. Private communication

[13] Sheldrick, G. M., (2017). SHELXL2017/1. Universität Göttingen: Göttingen, Germany.

[14] On Enantiomorph-Polarity Estimation, Flack, H.D. (1983). Acta Cryst., A39, 876-881.

[15] a) Hooft, R.W.W, Straver, L.H. & Spek, A.L. **2008**, *J. Appl, Cryst. 41*, 96-103; b) Thompson,A.L. & Watkin, D.J. **2009**, *Tetrahedron*.

[16] Bruker (2019) APEX3 (Version 2019.0) and (2016) SAINT (Version 8.37a). Bruker AXS Inc., Madison, Wisconsin, USA.

[17] a) An Empirical Correction for Absorption Anisotropy, Blessing, R. H. (1995). Acta Cryst., A51, 33-38; b) Sheldrick, G.M., SADABS (2016) Version 2016/2, 'Siemens Area Detector Absorption Correction' Universität Göttingen: Göttingen, Germany
